# An Epigenetic Signature in Peripheral Blood Predicts Active Ovarian Cancer

**DOI:** 10.1371/journal.pone.0008274

**Published:** 2009-12-18

**Authors:** Andrew E. Teschendorff, Usha Menon, Aleksandra Gentry-Maharaj, Susan J. Ramus, Simon A. Gayther, Sophia Apostolidou, Allison Jones, Matthias Lechner, Stephan Beck, Ian J. Jacobs, Martin Widschwendter

**Affiliations:** 1 Medical Genomics Group, University College London Cancer Institute, University College London, London, United Kingdom; 2 Department of Gynecological Oncology, University College London Elizabeth Garrett Anderson Institute for Women's Health, University College London, London, United Kingdom; Texas A&M University, United States of America

## Abstract

**Background:**

Recent studies have shown that DNA methylation (DNAm) markers in peripheral blood may hold promise as diagnostic or early detection/risk markers for epithelial cancers. However, to date no study has evaluated the diagnostic and predictive potential of such markers in a large case control cohort and on a genome-wide basis.

**Principal Findings:**

By performing genome-wide DNAm profiling of a large ovarian cancer case control cohort, we here demonstrate that active ovarian cancer has a significant impact on the DNAm pattern in peripheral blood. Specifically, by measuring the methylation levels of over 27,000 CpGs in blood cells from 148 healthy individuals and 113 age-matched pre-treatment ovarian cancer cases, we derive a DNAm signature that can predict the presence of active ovarian cancer in blind test sets with an AUC of 0.8 (95% CI (0.74–0.87)). We further validate our findings in another independent set of 122 post-treatment cases (AUC = 0.76 (0.72–0.81)). In addition, we provide evidence for a significant number of candidate risk or early detection markers for ovarian cancer. Furthermore, by comparing the pattern of methylation with gene expression data from major blood cell types, we here demonstrate that age and cancer elicit common changes in the composition of peripheral blood, with a myeloid skewing that increases with age and which is further aggravated in the presence of ovarian cancer. Finally, we show that most cancer and age associated methylation variability is found at CpGs located outside of CpG islands.

**Significance:**

Our results underscore the potential of DNAm profiling in peripheral blood as a tool for detection or risk-prediction of epithelial cancers, and warrants further in-depth and higher CpG coverage studies to further elucidate this role.

## Introduction

The role of epigenetics in cancer and other common complex diseases is undisputed [1], [2]. A unique facet of epigenetic marks that distinguishes them from their genetic counterparts, is their sensitivity to undergo alterations in response to dietary and other environmental exposures [1], [3], [4]. Given the epidemiological link between environmental factors and cancer it is natural to hypothesize that epigenetic mutations acquired during an individual's life, predispose the individual to the disease [1], [5]–[8]. Such a model is further supported by monozygotic twin studies that point to age and environmentally related epigenetic divergence as the cause of discordant disease status [9], [10]. Thus, it seems plausible that epigenetic changes associated with the environment and aging may themselves be related to cancer [11], and specifically that a number of these epigenetic mutations may constitute cancer predisposition markers.

More recently, DNA methylation (DNAm) of specific genes (*SEPT9, RASSF1A, APC*) in serum DNA have been proposed as diagnostic and prognostic biomarkers for colorectal cancer [12], [13] and breast cancer [14], respectively. DNA methylation (DNAm) changes associated with cancer and aging have also been observed in peripheral blood samples from postmenopausal women [15], [16]. Specifically, it has been suggested that known epidemiological risk factors (e.g high hormone levels) may leave DNAm imprints in peripheral blood DNA and that early detection of these marks could be used to predict the future risk of an individual developing cancer [15]. However, whether DNAm markers derived from peripheral blood may serve as diagnostic or risk-prediction tools remains controversial [17] and no study has yet evaluated their clinical potential on a genome-wide basis.

We performed genome-wide DNAm profiling of peripheral blood samples from a large ovarian cancer case-control cohort to help address the following questions. First, what effect does the presence of cancer have on the DNAm pattern in peripheral blood, a tissue that is unrelated to the cell of origin of an epithelial cancer, and more specifically, can the presence of cancer be predicted from the DNAm profile in blood? Second, can we identify methylation markers in blood that may serve as early detection or predisposition markers for ovarian cancer? Identification of reliable diagnostic or early detection biomarkers derived from blood is of great clinical and biological significance, specially for ovarian cancer where early detection is difficult [18]. Finally, following recent reports that most methylation variable positions are located outside of CpG-islands [19], we explored the genomic pattern of methylation variable positions in relation to CpG density.

## Results

### Age and Cancer Related DNA Methylation Patterns

All 540 peripheral blood samples were hybridised to 27 k Human Methylation Infinium beadchip arrays [20] (Materials and Methods, Table S1). A stringent quality control and inter-array normalisation procedure was used to remove confounding variation due to experimental factors, resulting in a normalised data matrix of methylation scores (β-values, 0<β<1) across 383 samples (148 healthy, 113 pre-treatment (preT) ovarian cancer cases, 122 post-treatment (posT) ovarian cancer cases) and 25,642 CpG sites (Materials and Methods, Figure S1). Singular value decomposition (SVD) of the normalised data demonstrated at least 10 significant components of variation with the largest components associated with phenotypic factors, notably presence of cancer and age (Figure S2, Materials and Methods).

### A DNA Methylation Signature Associated with Ovarian Cancer

We adopted a supervised approach to derive a cancer specific DNA methylation signature and to ask whether such a signature could be used to predict the disease status of blind test samples. Specifically, we argued that tumor presence may have a large enough impact on DNA methylation patterns that it ought to be detectable from peripheral blood samples in patients prior to undergoing treatment.

**Figure 1:**
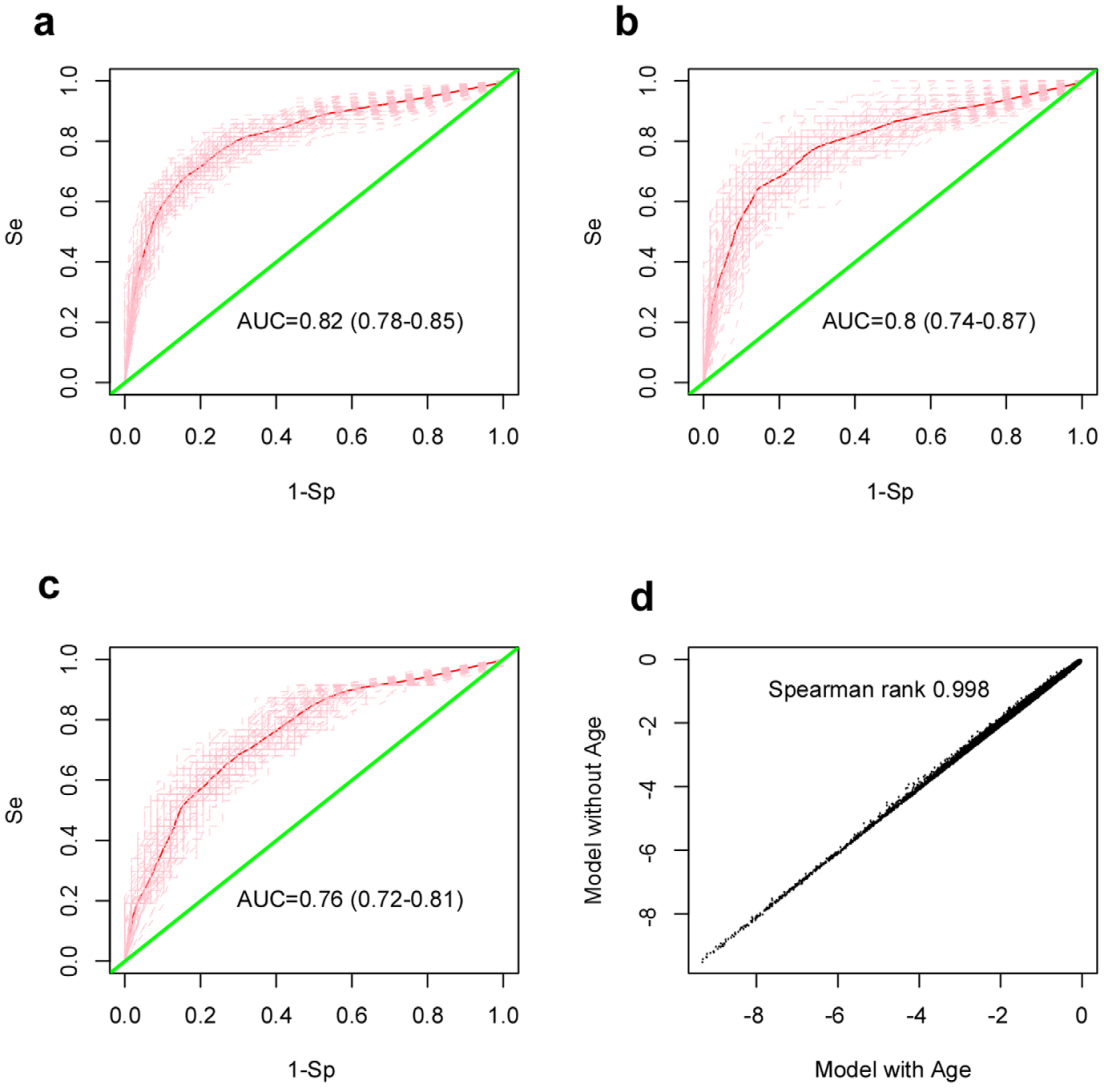
Prediction of tumor presence by a DNAm signature in blood. **a–b**) Classification performance of DNA methylation classifiers in **a**) training sets and **b**) blind test sets. Training and test sets consisted of blood samples from pre-treatment cases and healthy individuals (Materials and Methods). Average ROC curve over 100 different training/test set partitions with 95% CI envelope in blind test sets. Mean AUC and 95% CI over 100 different partitions are given. **c**) Classification performance in test sets consisting of healthy controls and post-treatment samples with evidence of active disease. **d**) Correlation between the ranking of top CpGs discriminating pre-treatment cases from healthy controls in regression models that included age (x-axis) and without age (y-axis) as a co-factor. Plotted are the log10(p-values) for the 25,642 CpG sites, as evaluated from multiple logistic regressions of case/control status against the CpG methylation profile with age as a co-factor (x-axis) and without age as a co-factor (y-axis). Spearman correlation between the two rankings is given.

To ensure that results were not biased to a specific choice of training/test set partition, we performed a total of 100 runs, each run using a different training/test set partition (Materials and Methods). For each choice of training set (90 controls and 70 preT samples), we used a multivariate logistic regression model (MVLR), with the CpG specific methylation profile as a predictor and including BSC (bi-sulphite conversion) efficiency, DNA input and batch effect as potentially confounding factors, to derive a p-value of association with case control status for each of the 25,642 CpG sites (Materials and Methods). Next, CpG sites were ranked according to their p-values and a shrunken centroid classifier trained on the top 1000 CpG sites (FDR<0.05, Materials and Methods). We observed that for the great majority of 100 runs, optimal (or close to optimal) classifier performance in internal cross-validations was obtained by selecting the top 100 CpG sites. Finally, in each run, the resulting top 100 CpG classifier was evaluated in a blind test set consisting of 58 healthy controls and 43 preT cases. Classifier performance on the training and test sets was evaluated by means of ROC curves and associated AUC (Figure 1a–b). Over the 100 runs, the mean AUC and 95%CI in the training and test sets was 0.82 (0.78–0.85) and 0.80 (0.74–0.87), respectively, indicating that the derived classifiers retained strong predictive power in the blind test sets (Figure 1a–b).

Next, we investigated whether the derived classifiers could predict cancer status of post-treatment samples with evidence of active disease (determined by CA125 serum levels >30) at the time of sample draw (47 samples). Averaged over the 100 runs, we obtained an AUC of 0.76 (0.72–0.81, 95%CI) in blind test sets consisting of 58 healthy controls and the (fixed) 47 postreatment samples (Figure 1c). Significant power to discriminate post-treatment samples with active disease from those without was also attained (AUC = 0.74, *P*<0.001). These results therefore confirmed the ability of the derived classifiers to predict the presence of tumors in post-treatment samples. In contrast, the classifiers did not predict the cancer status of post-treatment samples without evidence of active disease (70 samples) relative to healthy controls (AUC = 0.52 (0.48–0.55, 95%CI)).

Next, we asked whether the classification performance could be affected by age. To address this, we compared the ranking of the CpG sites in the supervised MVLR analysis with the corresponding ranking in a MVLR model that included age as a co-factor. This showed that p-values of association were largely unchanged and that both rankings were highly correlated (Spearman rank correlation = 0.998, Figure 1d), showing that the CpG sites on which our classification was based were predictive of cancer status independently of age.

### Cancer Diagnostic CpGs (CA-CpGs)

Having established that a DNA methylation signature from peripheral blood could be used to predict the presence of ovarian cancer, we next used all the healthy controls and pre-treatment samples to derive a final list of such “cancer diagnostic” CpGs (CA-CpGs).

**Figure 2:**
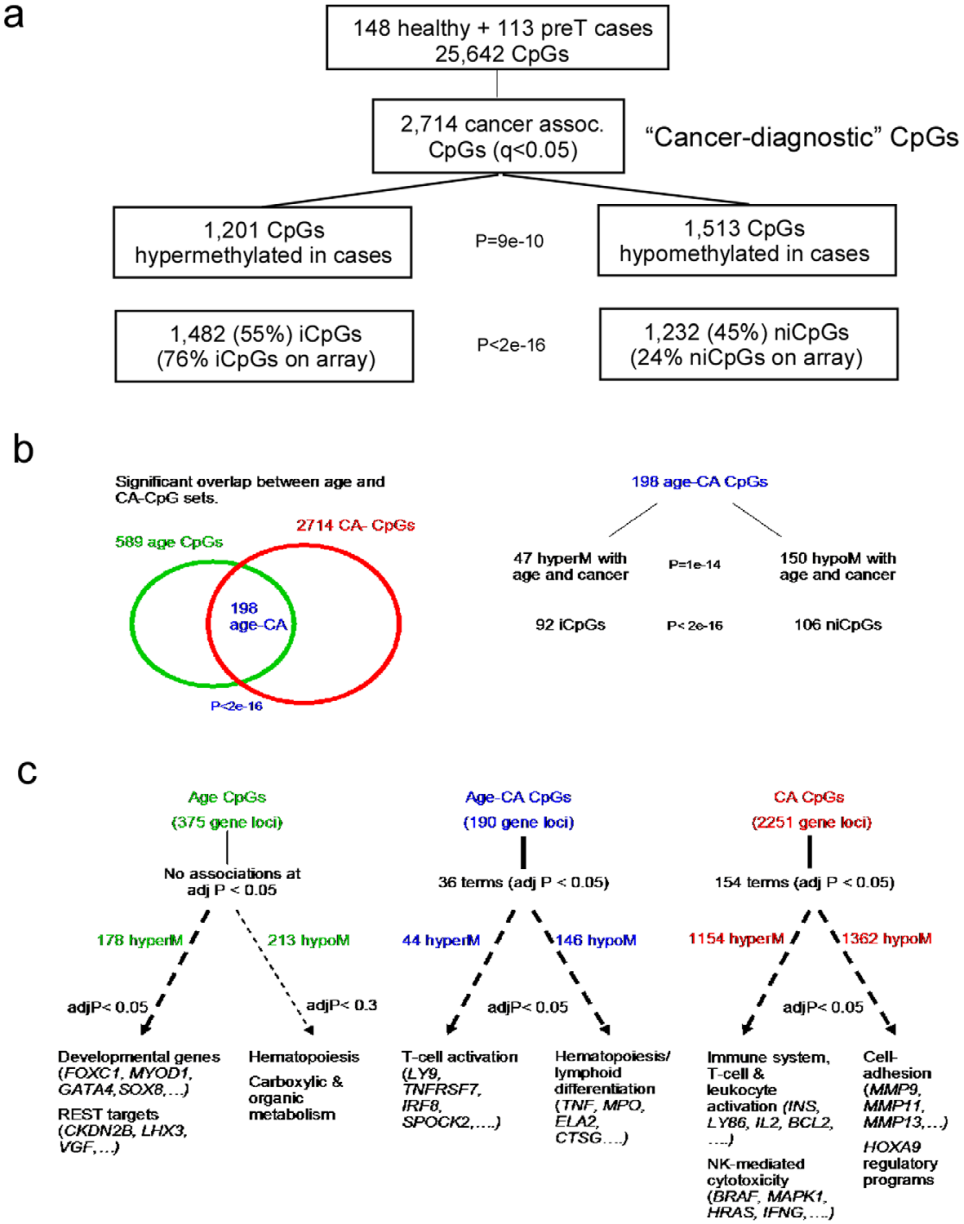
Cancer and age CpGs, and GSEA. **a**) Distribution of 2,714 CA-CpGs (FDR<0.05) in terms of hyper-and-hypomethylation (Binomial test P-value given), as well in relation to CpG localisation (Fisher's exact test). **b**) Overlap of age-CpGs with CA-CpGs (Fisher-test P-value of overlap given) and distribution of the 198 common age and CA-CpGs in terms of hypermethylated and hypomethylated patterns and iCpGs/niCpGs (Binomial and Fisher-test P-values are given, respectively). Out of the 198 CpGs, 47 exbited hypermethylation with age and cancer, 150 hypomethylation with age and cancer, 1 hyperM with age and hypoM with cancer and 0 showed hypoM with age and hypeM in cancer. **c**) Gene Set Enrichment Analysis for the common age CA-CpGs, age-specific CpGs (i.e age CpGs minus CA-CpGs) and CA-specific CpGs (i.e CA-CpGs minus age-CpGs) stratified according to hyper/hypometylation. Benjamini-Hochberg adjusted P-values are given. Most significantly enriched biological terms are given.

We identified a total of 2714 CA-CpGs passing an FDR threshold of 0.05 (Figure 2a, Table S2, Figure S3). We observed a skew towards hypomethylation with 1513 (56%) CA-CpGs showing lower levels of methylation in cases (Figure 2a, Binomial test *P* = 9×10^−10^). Even more strikingly, for the top 50 CpGs (47 unique gene loci), 41 (87%) were hypomethylated (Binomial test *P* = 10^−8^, Table S2). Of the 2714 CA-CpGs, 1482 (55%) and 1232 (45%) were located within (iCpGs) and outside (niCpGs) CpG islands [21], respectively. Given the overrepresentation of iCpGs on the array (76% iCpGs vs 24% niCpGs), the number of niCpGs associated with cancer was much higher than that expected by chance (Figure 2a, Fisher test *P*<2*e* ^−16^). The overrepresentation of niCpGs among CA-CpGs was also evident from inspection of the top 47 CA-CpG gene loci with 32 (68%) localised to niCpGs (Fisher test *P* = 6×10^−12^).

To further validate the diagnostic nature of the 2714 CpGs, we evaluated their overlap with the 520 CpGs discriminating post-treatment samples with and without active ovarian cancer (FDR<0.05, data not shown). This yielded an overlap of 355 CpGs (Fisher test *P*<2×10^−16^), confirming that effectively the same cancer diagnostic DNA methylation signature could have been derived in the post-treatment setting using CA125 levels as markers of tumour presence.

### Biological Significance of DNAm Signatures

To investigate the potential functional significance of the CA-CpG set we asked if there was specific enrichment of biological terms and pathways, including a large database of functional gene expression signatures [22]. Recently, we showed that aging also has a significant impact on the DNAm pattern of peripheral blood and identified 589 CpGs significantly associated with age (FDR<0.05) [16]. Thus, in order to dissect the roles of age and cancer we performed Gene Set Enrichment Analysis (GSEA) [22] on CpGs uniquely associated with age and cancer, as well as on the 198 CpGs (190 unique gene loci) shared by the age and CA-CpG lists (Figure 2b, Table S2). We note that this overlap was highly significant (Figure 2b, Fisher test *P*<2×10^−16^), suggesting that age and tumor presence elicit common changes in the DNAm pattern of peripheral blood. GSEA revealed functional associations (adjusted *P*<0.05) of four main categories of genes (Figure 2c, Table S3): (i) *REST*-targets and developmental genes [23], [24] with hypermethylated age-CpGs, (ii) genes involved in hematopoiesis and lymphoid-myeloid differentiation with hypomethylated age and age-CA CpGs, (iii) genes involved in T-cell activation and natural-killer (NK) mediated cytotoxicity with hypermethylated cancer-specific CpGs, and (iv) genes involved in cell-adhesion and *HOXA9* regulatory programs with hypomethylated cancer-specific CpGs.

To understand these functional associations we hypothesized that some of these may reflect variations in blood cell type composition, as this is known to vary with both age and tumor presence [25]–[29]. To investigate this further, we asked if genes known to be differentially expressed between main blood cell types [30] were overrepresented in the age and CA-CpG lists. Several significant associations were found, which were more pronounced for the cancer than the age-associated signatures (Table 1). Specifically, among CpGs hypomethylated in cancer cases, there was enrichment of genes known to be upregulated in granulocytes (*P* = 2×10^−5^, Table 1), while CpGs hypermethylated in cancer were enriched for genes known to be upregulated in T-cell lymphocytes (CD4+ *P* = 0.002, CD8+ *P* = 0.01, Table 1, Table S4), consistent with reports of a higher granulocyte/lymphocyte ratio in the blood of cancer cases compared to healthy controls [26], [29]. To test this further we compared methylation levels of those CpGs mapping to genes upregulated in granulocytes and lmphocytes between healthy controls and post-treatment cases with and without active disease (as determined by CA125 levels) (Figure S4). As expected, genes upregulated in granulocytes were significantly hypomethylated in post-treatment cases with positive CA125 levels relative to controls, while this was not so for post-treatment cases without active disease (Figure S4). Similarly, genes upregulated in lmphocytes were significantly hypermethylated in post-treatment cases with positive CA125 levels relative to controls, while there was no difference when comparing post-treatmant cases without active disease to controls (Figure S4).

### Age-Dependent DNAm Signature Predicts Tumor Presence

**Figure 3:**
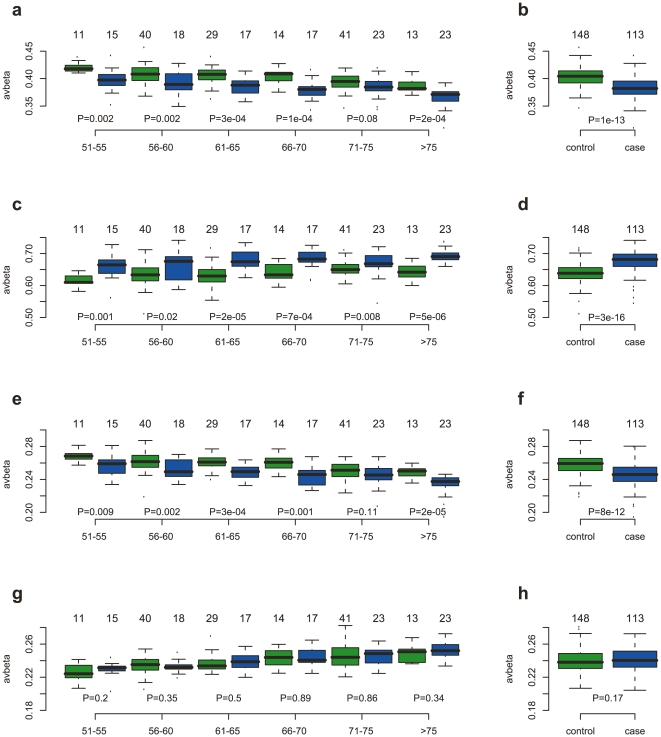
Age-dependent methylation patterns are associated with ovarian cancer. Average methylation patterns of age-associated CpGs selected through supervised analysis. (**a–b**) age hypomethylated niCpGs. (**c–d**) age hypermethylated niCpGs. (**e–f**) age hypomethylated iCpGs. (**g–h**) age hypermethylated iCpGs. (**a,c,e,g**) Average methylation (y-axis) of controls (green) and cases (blue) for each of the six age groups (x-axis) (50–55,55–60,60–65,65–70,70–75,>75). (**b,d,f,h**) Average methylation versus disease status (all age groups combined). All P-values are from a two-tailed Wilcoxon rank sum test. In all panels, we give the numbers of samples in each group above the corresponding boxplot. Cases are pre-treatment samples.

The strong overlap between the age and cancer associated CpGs and the functional enrichment of genes involved in myeloid-lymphoid differentiation indicated to us that age and cancer cause the same changes in DNAm patterns by independently eliciting the same underlying changes in blood cell type composition. We therefore hypothesized that age-specific DNAm changes may be aggravated in patients with ovarian cancer. To test this, we first computed average methylation levels over CpGs undergoing specific hyper or hypomethylation with age. These patterns showed the expected correlations with age in healthy controls and pretreatment cancer cases (Figures 3a,c,e,g & Figure S5). However, we also observed that the average methylation values were significantly different between pretreatment cases and controls, with lower average methylation in cases versus controls for age hypomethylated niCpGs (Figure 3b, Wilcox test *P* = 1×10^−13^) and iCpGs (Figure 3f, *P* = 1×10^−11^), and correspondingly higher average methylation levels in cases compared to controls for niCpGs hypermethylated with age (Figure 3d, *P* = 3×10^−16^). Importantly, these associations with disease status were independent of age group for the hypomethylated niCpGs and iCpGs (Figures 3a,e). For the hypermethylated age CpGs, we observed a corresponding pattern of hypermethylation in cancer in all age groups for niCpGs (Figure 3c), but not so for iCpGs (Figures 3g,h).

**Figure 4:**
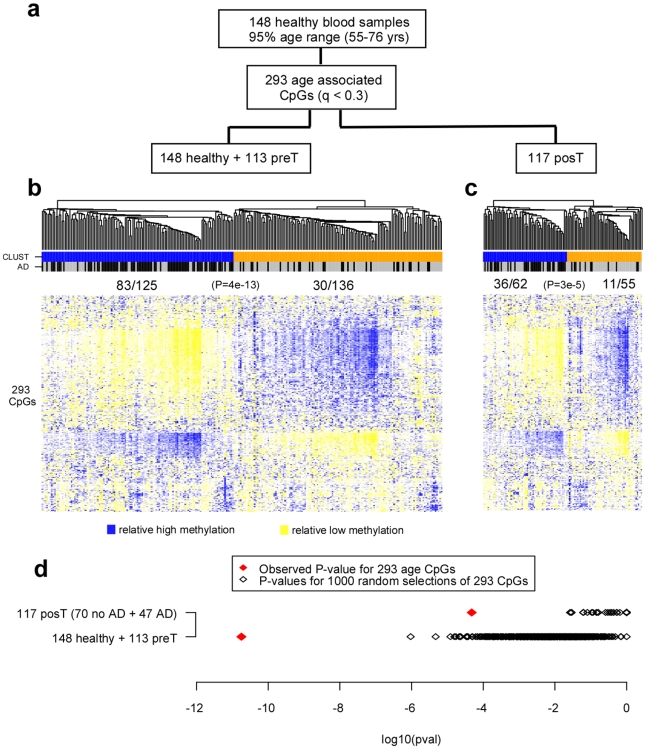
Clustering of samples over age-associated CpGs. **a**) Multivariate linear regression of age in 148 healthy blood samples against CpG methylation profiles adjusting for BSC efficiency, batch and DNA input effects, identified 293 CpGs at q(FDR)<0.3. **b**) Hierarchical clustering of the 148 healthy controls and 113 pre-treatment (preT) cases over the 293 CpGs. The two main clusters (CLUST) predicted by the algorithm are labelled as blue and orange. Case control status is indicated as active disease (AD): case = black, control = grey. **c**) Hierarchical clustering of 117 post-treatment cases over same 293 CpGs. Of the 117 post-treatment cases, 47 and 70 had recurrent (black) and no recurrent (grey) active disease (AD) at sample draw, respectively. The two main clusters (CLUST) predicted by the algorithm are labelled as blue and orange. In the heatmaps, CpG specific methylation β-values were standardised to zero mean and unit variance for sake of clarity (blue: high relative methylation, yellow = low relative methylation). In panels b) and c) we give the number of samples with active disease at sample draw in each cluster, and give the corresponding Fisher's exact test P-value. **d**) Comparison of observed P-values with those obtained by 1000 random selections of 293 CpGs. P-values were computed from Fisher's exact test for the two clusters inferred from applying a Gaussian mixture model [43].

To further demonstrate that age-related DNAm patterns were independently associated with tumor presence, we asked if the age associated CpGs derived from the 148 healthy controls (293 CpGs with FDR<0.3) [16] were able to discriminate samples according to disease status (Figure 4a). Unsupervised hierarchical clustering over the 293 CpGs segregated cases from controls (Figure 4b, Fisher test *P* = 4×10^−13^). The same age-CpGs were also able to discriminate cases with recurrent active disease from those without (as measured by serum CA125 levels at sample draw) in an independent set of blood samples from 122 post-treatment cases (Figure 4c, Fisher test *P* = 3×10^−5^). To further establish the significance of these findings, in none of 1000 random selections of 293 CpGs did we observe P-values as extreme as these (Figure 4d).

### Cancer-Predisposition CpGs

It is plausible that a small number of the 2714 CA-CpGs are bona-fide cancer-predisposition markers. We hypothesized that some of these risk markers might be detectable from the 70 post-treatment peripheral blood samples of patients who did not have recurrent disease at the time of sample draw but who could eventually develop recurrent disease, since such samples might mimick the pre-clinical predisposition stage. To determine rigorously whether such a risk CpG signature exists, we applied a state-of-the-art Surrogate Variable Analysis (SVA) [31], [32] framework, which models hidden and potentially confounding factors to obtain a more accurate estimate for the FDR (Materials and Methods). Using SVA we obtained 84 CpGs passing a FDR threshold of 0.4, suggesting that on average about 50 CpGs may be discriminatory between post-treatment samples without active disease (CA125 serum levels <30) and healthy controls (Figure S6a, Table S5). Since it is possible that some of these reflect methylation changes due to treatment and since a risk CpG should also be diagnostic, we deconvoluted the effect of treatment by finding the overlap between the 84 CpGs and the 2714 CA-CpGs (unaffected by treatment), which yielded an overlap of 18 “cancer-predisposition” risk CpGs (Fisher test P = 0.003, Figure S6b, Table S5). Of interest, this list included *TSG101*, a gene with putative tumour suppressor roles and a candidate breast cancer predisposition gene [33].

## Discussion

Here we have performed the first large scale (over 300 samples) genome-wide study of DNAm profiles using a state-of-the art platform that measures the methylation state of over 27,000 CpGs. We have shown that ovarian cancer has a significant impact on the DNAm pattern of peripheral blood cells. While the epigenetic signature we have presented still lacks the high specificity necessary for an immediate diagnostic application, the fact that active ovarian cancer could be predicted with a relative high accuracy (AUC = 0.8) from a DNAm profile in blood clearly demonstrates the future potential of epigenetic profiling as a diagnostic tool.

In addition, we provided evidence for the existence of DNAm markers that may serve as early detection or predisposition markers for ovarian cancer. Of the 18 candidate risk markers, 11 and 7 showed hyper and hypomethylation in cancer, respectively, with *TSG101* and the pre-mRNA splicing factor *SFRS6* both undergoing hypermethylation in cancer. Further confirmation that the markers identified here may serve as early detection or predisposition markers for ovarian cancer will require a large prospective study, which is currently ongoing [34].

The observed DNAm patterns can be summarised in terms of two biologically distinct signatures. First, the observation that a DNAm signature for aging, characterised by differential methylation of genes with hematopoietic cell lineage and immune system functions is aggravated in the presence of ovarian cancer, suggests that an epithelial tumour and aging elicit common changes in the cellular composition of peripheral blood. This interpretation is further supported by the fact that the same biological terms were strongly enriched among genes differentially expressed between blood cell types [30] (Table S6), and that genes commonly upregulated in granulocytes and lymphocytes showed a differential methylation pattern (Table 1, Figure S4) consistent with an increased granulocyte to lymphocyte ratio in response to aging or cancer. It is significant that there is independent evidence that both aging and cancer presence lead to a myeloid-skewing in the myeloid/lymphoid differentiation program, with a corresponding higher granulocyte/lymphocyte ratio [25]–[29], a pattern consistent with our observation that granulocyte and T-lymphocyte specific genes were enriched among CpGs hypo-and hypermethylated in cancer, respectively. Since this effect was inferred from DNAm profiles, it would appear that the expression of a substantial number of genes characterising bood cell types is under direct epigenetic regulation.

Another related question is whether the diagnostic epigenetic signature is specific to ovarian cancer. If different cancers elicit similar immune responses and thus similar changes in blood cell type composition, we could expect a proportion of the identified diagnostic signature to be non-cancer specific. However, to conclusively determine that this is the case and that age and cancer are not mediating immuno-compromising effects via epigenetic modification of particular cell-types, requires additional data not provided by our study. Similarly, whether parts of the observed cancer signatures may be causally involved in the disease must await further investigations.

A second DNAm signature was characterised by CpGs undergoing hypermethylation with age and was highly enriched for developmental genes and *REST*-targets. Given that *REST* (*NRSF*) is involved in suppression of genes that are required for differentiation of embryonic and adult stem cells [24], the age-induced hypermethylation of *REST*-targets, if confirmed in a stem-cell population, may represent a generic mechanism for age-associated loss of stem-cell function and increased predisposition to cancer [5].

Finally, our finding that most of the methylation variability is associated with niCpGs further supports the view that most of the phenotypically relevant DNAm variation is to be found in regions other than CpG islands [19]. Confirming this further, the observed association between gene expression of different blood cell-types and the pattern of DNA hypo and hypermethylation was much stronger for niCpGs than iCpGs (data not shown).

In summary, this work demonstrates that DNAm profiling in blood holds significant promise as a future diagnostic or risk-prediction tool and warrants further higher CpG-coverage studies to fully elucidate this role.

## Materials and Methods

### Description of UKOPS Sample Collection

A total of 540 whole blood samples were drawn from the UK Ovarian Cancer Population Study (UKOPS) for inclusion in this study. Cases (n = 266) consisted of postmenopausal women diagnosed with primary epithelial ovarian cancer. Half of the cases (pre-treatment cases; n = 131) gave their blood at the time of their diagnosis prior to treatment and the other half (post-treatment cases; n = 135) gave their blood at some stage during their follow up visits after primary treatment (2.4±2.7 years between diagnosis and blood sample taken). Controls (n = 274) were apparently healthy postmenopausal women recruited from the UK Collaborative Trial of Ovarian Cancer Screening (UKCTOCS) [34] for which annual serum samples are available. Recruitment took place at 8 participating hospitals within the UK. Women with a previous history of bilateral oophorectomy and/or cancer were excluded from the study. All cases and healthy controls were postmenopausal and age-matched and detailed clinical characteristics are given in Table S1.

### Sample Processing

Blood samples were collected by the study nurse at the regional centres in tubes (9 mL K3 EDTA Vacuette tubes, manufactured by Greiner Bio One) and were frozen within 3 hours of collection. The samples were stored at −80°*C* at the regional centre and were shipped to the central laboratory at UCL on a quarterly basis. Once received in the central laboratory, the samples were logged and transferred to −80°*C* freezer until the DNA was extracted. Serum CA125 concentrations were determined by electrochemiluminescence sandwich immunoassay on an Elecsys 2010 (Roche Diagnostics, Burgess Hill, UK) using two monoclonal antibodies (OC125 and M11; Fujirebio Diagnostics AB, Göteborg, Sweden), and values >30 were taken as a marker of active disease [35]. Over 95% of pre-treatment cases had CA125>30, while among post-treatment cases about 40% had active recurrent disease (CA125>30).

### DNA Extraction and Bisulphite Modification

The DNA was extracted at Tepnel, using a chloroform based extraction method and 800 ng (2×400 for each sample). Average DNA concentration was 33.0±17.4 ng/µL. DNA from each sample was bisulphite modified using the EZ DNA Methylation Kit D5008 (Zymo Research, Orange, CA, USA) according to the manufacturer's instructions.

### Illumina Infinium Assay

Methylation analysis was performed using the Illumina Infinium Human Methylation27 BeadChip. Briefly, bisulphite converted DNA was amplified, fragmented and hybridised to the BeadChip arrays (each chip accommodates 12 samples as designated by Sentrix positions A–L). A single base extension was then performed using labelled DNP- and biotin labelled dNTPs. The arrays were imaged using a BeadArray™ Reader. Image processing and intensity data extraction were performed according to Illumina's instructions. Each interrogated locus is represented by specific oligomers linked to two bead types: one representing the sequence for methylated DNA (*M*) and the other for unmethylated DNA (*U*). The methylation status of a specifc CpG site is calculated from the intensity of the *M* and *U* alleles, as the ratio of fluorescent signals β = *Max*(*M*,0)/[*Max*(*M*,0)+*Max*(*U*,0)+100]. DNA methylation β values are continuous variables between 0 (absent methylation) and 1 (completely methylated) representing the ratio of combined locus intensity.

### Experimental Design

A total of 540 samples (274 healthy, 131 pre-treatment, 135 post-treatment) and 12 controls (methylation control consisted of a single pool of fully methylated Sss1 treated genomic DNA to monitor for batch to batch variation) were hybridised to the Illumina Infinium platform, distributed across 11 batches of 48 samples each (4 sentrix chips of 12 samples per batch) and 1 batch of 24 (2 sentrix chips). Pre-treatment and post-treatment cases and controls were randomised across batches and within each batch across beadchips, and included an average of 29 cases and 17 healthy samples for batches 1–9. Due to logistic reasons there was an over representation of controls in batches 10–12. Due to batch effects, these were later excluded from statistical analysis to avoid any bias.

### Quality Control

Background corrected *U* & *M* values, β values (as generated from the Beadstudio software) and built-in controls were used to evaluate the quality of individual arrays. Samples with low bisulphite (BS) conversion efficiency (BS control intensity values <4000) were excluded, as well as other outliers that we detected using boxplots of total intensity *I* = *U*+*M* values and histograms of β-values. After this first QC step, 502 samples remained. Next, samples were filtered further according to CpG coverage, using the Beadstudio p-values of detection of signal above background. Specifically, we computed the global (inter-sample) CpG coverage for different minimum levels of CpG coverage per sample: demanding at least 95% coverage per sample gave 93% global coverage (25,642 CpGs) across 495 samples, resulting in a β-valued data matrix of dimension 25,642×495.

### Diagnostic SVD Analysis

Given the β-valued data matrix over 495 samples and 25,642 CpG sites, we performed a singular value decomposition (SVD) to determine the nature of the largest components of variation. SVD has been successfully applied to methylation β-valued data before [36]. We focused on the top 20 principal components and correlated these to experimental factors, including batch, well position, sentrix chip, BS conversion efficiency (as assessed using the built-in BS conversion efficiency controls) and DNA input, as well as phenotypic factors, including case control status. This analysis showed that most of the variation was associated with potentially confounding batch, DNA input and BS conversion (BSC) efficiency effects, thus requiring careful inter-array normalisation procedures. Because three batches consisted overwhelmingly of healthy control samples and thus were entirely confounded, these were removed, yielding a β-matrix over 25,642 CpGs and 383 samples (148 healthy controls, 113 pretreatment samples, 122 postreatment samples) for further analysis.

To perform the SVD analyses, imputation of missing β-valued data was necessary and was accomplished using the k-nearest neighbours procedure [37]. The missing β-values were caused by probes with only a few good quality bead-level replicates, for which therefore no β-values were reported. We verified however that imputation gave almost identical values to the alternative procedure of recalculating β-values from the bead-replicate averaged *U* and *M* values, thus validating both imputation approaches.

### Inter-Array Normalisation

Normalisation across arrays was performed initially using a variety of strategies: (a) do-nothing (b) separate quantile normalisation of the U and M channels and recomputation of β-values, (c) quantile normalisation of β-values, (d) quantile normalisation followed by adjustment for batch, DNA input and BSC efficiency effects, and (e) adjustment for batch, DNA input and BSC efficiency effects. The various strategies were evaluated in two ways. First, we asked if the median β-values per sample were strongly correlated with any of the unwanted factors such as BSC efficiency. A more stringent evaluation was provided by a SVD to check whether components of variation were correlated with unwanted factors. The median-based analysis (and SVD) indicated that methods (a) (Figure S1a) and (b) (not shown) were inadequate. SVD also showed that quantile normalisation (method (c)) did not remove all unwanted variation (Figure S1b). However, adjusting the quantile normalised data for BSC efficiency, DNA input and batch effects (method (d)), by appropriate inclusion of co-factors in a multivariate regression model, we succeeded in peeling away almost all of the unwanted variability (Figure S1c). Method (e) also performed optimally and results did not vary appreciably between methods (d) and (e) (not shown). After inter-array normalisation using either method (d) or (e), the largest components of variation were associated with phenotypic factors such as age and cancer (Figure S1c).

### Significance of Singular Values

The statistical significance of the components of variation inferred using SVD was evaluated against the null distribution obtained by considering random matrices. The normalised adjusted data was randomised by permuting the CpGs, using a distinct permutation for each sample. Subsequently, SVD was performed on the randomised data matrix and the fraction of variation of the inferred singular values compared to the fractions of variation of the unpermuted data (Figure S2). We verified using multiple randomised matrices that the null distribution of singular values is very tight (Figure S2), allowing significance to be estimated from as little as 5 permutations.

### Supervised Analysis

Associations between CpG β-valued methylation profiles and phenotypes of interest were carried out using robust linear regressions for ordinal/continuous phenotypes (age) or logistic regressions for binary phenotypes (case/control status). Multivariate regressions were performed for each CpG separately and included factors for batch, DNA input and BSC efficiciency effects. To correct for multiple testing we estimated the false discovery rate (FDR) using the q-value framework [38]. Since CpG sites within the same CpG-island may exhibit similar methylation profiles, and given that 7,528 CpG islands contained more than one CpG site, we also estimated the FDR using a permutation approach that would take the correlations of CpG sites into account. Specifically, sample labels were permuted (same permutation over all CpGs) and supervised analyses carried out on the resampled data set, using a total of 100 permutations to obtain reasonable estimates of the FDR. We found however that FDR estimates using the permutation approach were very similar to those estimated using the q-value framework at the significance levels of relevance (Figure S3). The q-value method was thus adopted for computational convenience. FDR estimates were further confirmed with Surrogate Variable Analysis (SVA) [31], [32]. SVA allows more accurate FDR estimates to be obtained by including potentially hidden (i.e unknown) in addition to known confounding factors in the multivariate regression [31], [32]. SVA models the known and hidden confounding factors using a SVD on the residual variation matrix that remains after regression of the β-matrix to the phenotype of interest, and generally yields a more accurate FDR estimate [31], [32].

### Classification Analysis

The following strategy was used to determine whether a DNA methylation signature from peripheral blood could be used to predict the presence of the tumour. (1) A training set of 90 healthy controls and 70 pretreatment (preT) cases was selected at random. (2) Using the training set, a multivariate logistic regression of case control status against the β profile of a CpG site was performed for each of the 25,642 CpG sites and adjusting for BSC efficiency, DNA input and batch effects. (3) CpG sites were ranked according to the magnitude of the regression coefficient and associated p-values of significance. (4) p-values were transformed into q-values [38] to provide an estimate of the false discovery rate (FDR). (5) Using the top 1000 CpGs (these all passed a 0.05 FDR threshold) we then applied a nearest shrunken centroid classifier [39] to obtain methylation centroids for cases and controls. Optimal classifier performance as a function of the degree of shrinkage (number of CpG sites) was monitored and an optimal (or near optimal) classifier selected based on the top 100 CpG sites. (6) Next, using this centroid classifier we computed the posterior probability for each sample in the blind test set (58 healthy & 43 preT) to be a case. Thus, the probability of cancer status can be viewed as a continuous predictor and performance evaluated using ROC and AUC measures. (7) This analysis was repeated for 100 different training test set partitions and the average AUC and 95% confidence interval envelopes in the training and test sets was recorded.

### Clustering of Methylation Profiles

Age-associated CpGs were derived from the 148 healthy control samples using the previously described supervised analysis (293 CpGs passed an FDR threshold of <0.3). The sample set of 148 healthy controls and 113 preT cases were then clustered over these 293 CpGs using a hierarchical clustering algorithm with Pearson correlation metric and average linkage. Prior to clustering, CpG β-profiles were standardised to mean zero and unit variance across samples. The same clustering procedure was applied to the 122 post-treatment samples, of which 117 had CA125 serum level data at the time of sample draw. Dendrograms were cut to yield two main clusters to highlight the clustering of samples with active disease (i.e preT or posT with CA125>30) and without active disease (i.e healthy samples or post with CA125<30). Non-random associations of disease status with the two main clusters were tested using two-tailed Fisher's exact test. To establish the significance of the clustering in relation to all CpGs found on the array, 1000 random selections of 293 CpGs were performed and samples reclustered. A P-value of significance was then obtained by estimating the fraction of times (out of 1000) that the Fisher-test P-value was as extreme as the observed one.

### Gene Set Enrichment Analysis

Given a list of CpGs, these were mapped to promoters and unique gene loci and then tested for enrichment of biological terms and pathways using the Gene Set Enrichment Analysis (GSEA) and the Molecular Signatures Database (MSigDB) tool [22]. Significant associations were confirmed with an independent method, EASE [40].

### Software

All computations and statistical analyses were performed using *R 2.8.1 (* http://www.r-project.org *)* [41] and *Bioconductor 2.3 (* http://www.bioconductor.org *)* [42].

## Supporting Information

Figure S1 Diagnostic SVD analysis: Heatmap of p-values of association between the top 20 singular vectors (principal components) from the singular value decomposition (SVD) of the beta-valued data matrix and phenotypic as well as experimental factors. Phenotypic factors included case control status (coded as 0,1), stage of cancer (0 = stage1 or 2, 1 = stage3 or 4), grade (1,2,3), histological subtype (clear cell, endometriod, serous, other) and age at sample draw coded as (1 = 50–55, 2 = 55–60, 3 = 60–65, 4 = 65–70, 5 = 70–75, 6 = 75+). Experimental factors included bisulphite conversion efficiency controls (BSC1 & BSC2), DNA input and batch number. P-values coded as follows: P<10e-10 (darkred), 10e-10<P<10e-5 (red), 10e-5<P<0.01 (orange), 0.01<P<0.05 (pink), P>0.05 (white). a) Before inter-array quantile normalisation, b) After inter-array quantile normalisation. c) After adjustment for BSC, DNA input and batch effects.
(0.03 MB PDF)

Figure S2 Significance analysis of singular values: Statistical significance analysis of singular values inferred from an SVD decomposition of the normalised adjusted data. x-axis denotes singular values ranked according to magnitude of variation. y-axis denotes the fraction of variation in the data explained by that singular value. Red points show the observed fractions, black points denote the fractions of variation under a random reshuffling of the data [Leek et al. 2008]. There are approximately 11 significant components of variation explaining about 24% of the variation in the data.
(0.02 MB PDF)

Figure S3 FDR estimation using permutation of sample labels: Top diagram plots the sorted log10(pvalues) (y-axis) of association with cancer (from logistic regression) against CpG index (x-axis). Black denotes observed p-values, green denotes corresponding values obtained after permutation of sample labels. Lower diagram compares the estimated mean number of false positives (y-axis) against the number of positives (x-axis) (i.e., the number of tests passing a given significance threshold). In blue, we show the estimate from the permutation approach; in red, the analytical estimate from the q-value. At an FDR∼0.05 both methods predict a similar number of significant CpGs.
(3.86 MB PDF)

Figure S4 DNA methylation levels of granulocyte and lmphocyte markers: Average methylation levels (y-axis) of CpGs mapping to genes upregulated in granulocytes and lymphocytes against different disease states: H (healthy control samples, n = 148), CA125- (post-treatment cases with CA125<30, n = 70), CA125+ (post-treatment cases with CA125>30, n = 47). P-values from two-tailed Wilcoxon-tests between H and CA125- and between H and CA125+ are shown.
(0.01 MB PDF)

Figure S5 Age-dependent methylation patterns are associated with ovarian cancer: a–b) Average methylation patterns of age anti-correlated niCpGs selected through supervised analysis.(a) Average methylation versus age group for controls and cases. (b) Average methylation versus disease status. c–d) Average methylation patterns of age correlated niCpGs selected through supervised analysis.(c) Average methylation versus age group for controls and cases. (d) Average methylation versus disease status. e–f) Average methylation patterns of age anti-correlated iCpGs selected through supervised analysis.(e) Average methylation versus age group for controls and cases. (f) Average methylation versus disease status. g–h) Average methylation patterns of age correlated iCpGs selected through supervised analysis.(g) Average methylation versus age group for controls and cases. (h) Average methylation versus disease status. In panels b,d,f,h, p-values are from a two-tailed Wilcoxon rank sum test (0 = controls, 1 = case). In panels a,c,e,g, we give the numbers of samples in each age group and P-values reflect strength of the linear regression. Age groups are coded as (1 = 50 to 55, 2 = 55–60, 3 = 60–65, 4 = 65–70, 5 = 70–75, 6 = over75). Cases are pretreatment samples.
(0.05 MB PDF)

Figure S6 Derivation of cancer predisposition/risk CpGs: a) Histogram of p-values from multivariate logistic regression models (MVLR) comparing cancer status of postreatment patients without active disease at sample draw (70 samples) with age-matched healthy controls (148 samples). Logistic regression models included cancer status as a binary response and the CpG methylation profile as a predictor with batch, bisulphite conversion and DNA input as co-factors. Histogram distribution is relatively flat indicating the absence of discriminatory CpGs. Using Surrogate Variable Analysis (SVA+MVLR) to model all confounding known and hidden factors, p-value distribution exhibits a skew towards significant p-values, suggesting the existence of discriminatory CpGs. b) To deconvolute the effects of tumor-presence and treatment, cancer predisposition or risk CpGs should be given by the overlap of cancer-diagnostic CpGs with the 84 CpGs (FDR (q)<0.4) that discriminate postreatment cases without active disease (AD) from healthy controls. This yielded 18 candidate ovarian cancer risk CpGs.
(0.08 MB PDF)

Table S1 Clinical characteristics of samples.
(0.06 MB PDF)

Table S2 List of cancer diagnostic CpGs (CA-CpGs).
(0.58 MB PDF)

Table S3 Summary of Gene Set Enrichment Analysis results on CpGs in blood undergoing significant hyper- and hypomethylation with age and with presence of ovarian cancer.
(0.06 MB XLS)

Table S4 Enrichment analysis of genes undergoing age and cancer specific DNAm changes and genes upregulated in major blood cell-types.
(0.01 MB XLS)

Table S5 Discriminatory CpGs between postreatment samples without active disease and healthy controls.
(0.04 MB PDF)

Table S6 Gene Set Enrichment Analysis of genes differentially expressed between major blood cell types.
(0.17 MB XLS)
