# Supplementary figures and images for: An Epigenetic Signature in Peripheral Blood Predicts Active Ovarian Cancer
Source: PLoS One. 2009 Dec 18;4(12):e8274. doi: 10.1371/journal.pone.0008274 (PMC2793425; doi:10.1371/journal.pone.0008274)

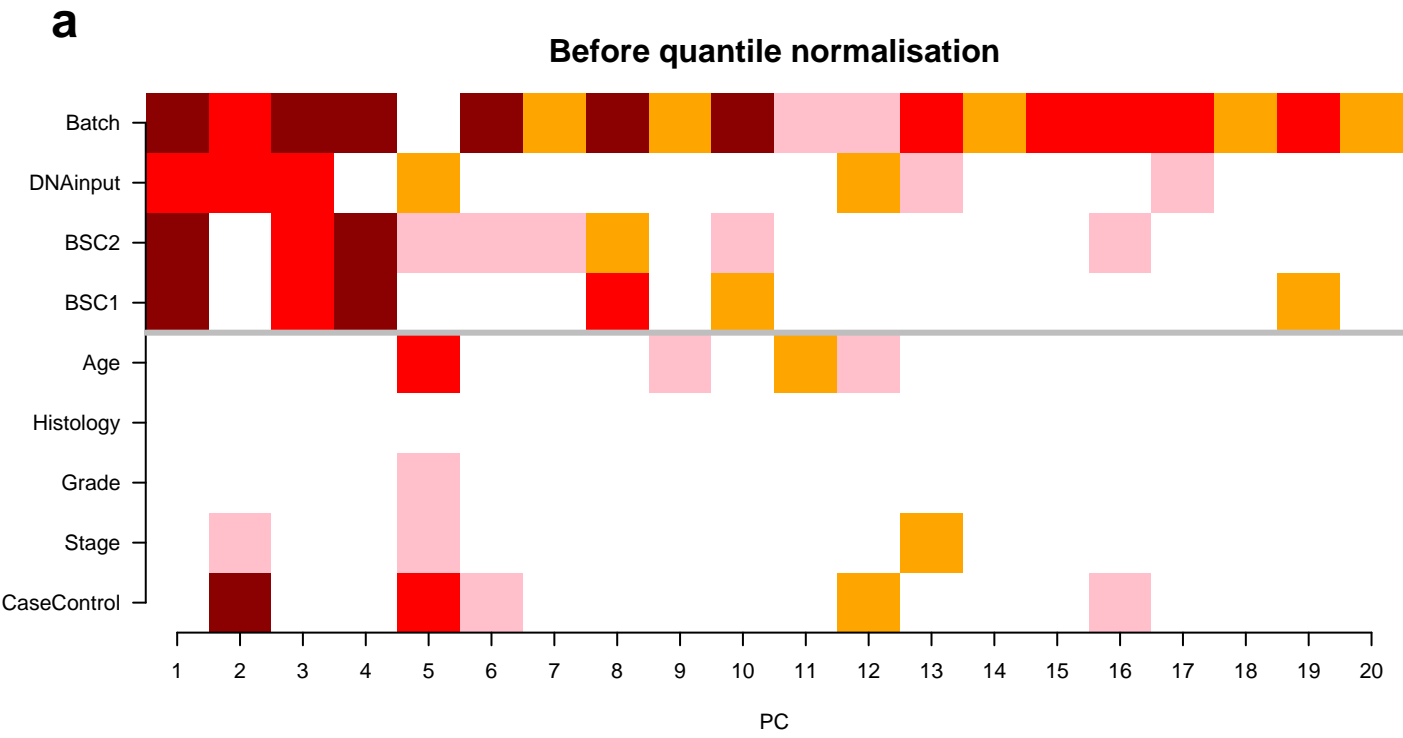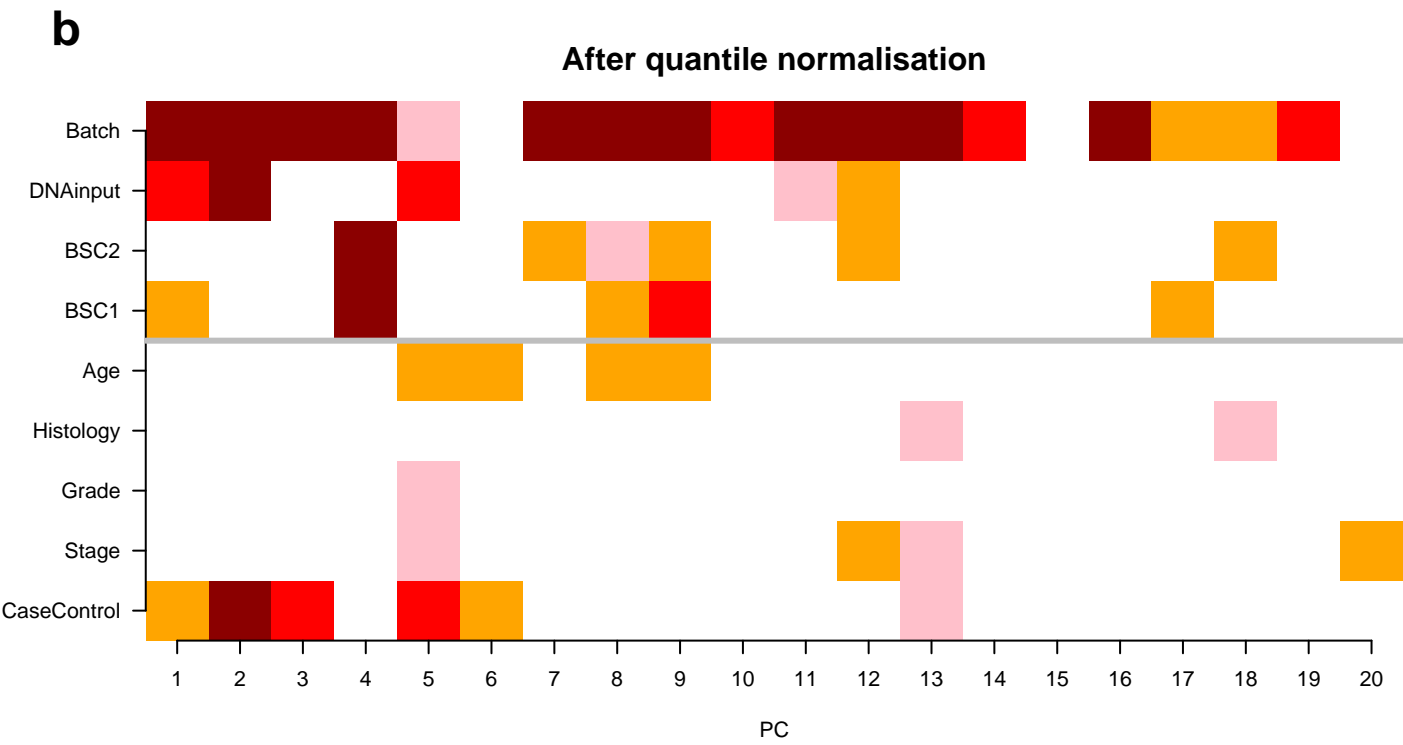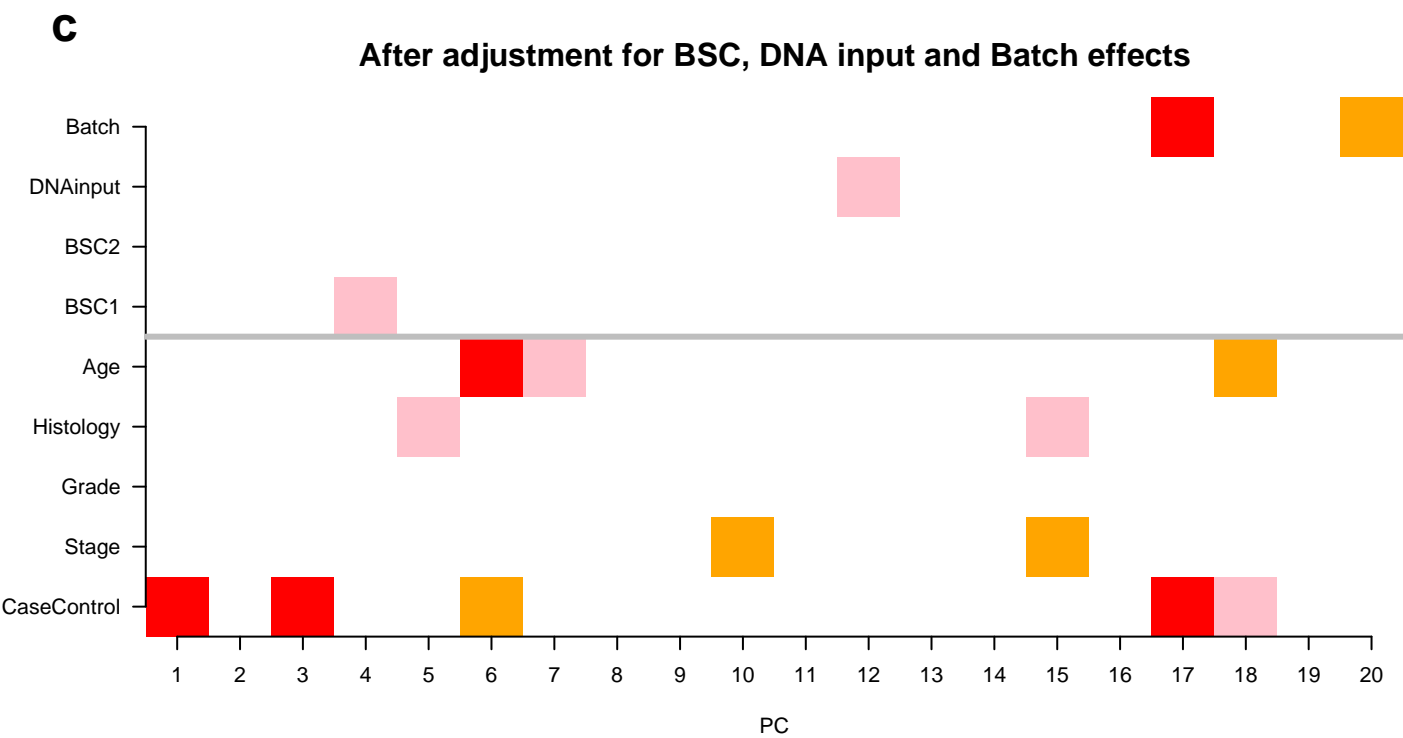

Supplement: Figure S1 — Diagnostic SVD analysis: Heatmap of p-values of association between the top 20 singular vectors (principal components) from the singular value decomposition (SVD) of the beta-valued data matrix and phenotypic as well as experimental factors. Phenotypic factors included case control status (coded as 0,1), stage of cancer (0 = stage1 or 2, 1 = stage3 or 4), grade (1,2,3), histological subtype (clear cell, endometriod, serous, other) and age at sample draw coded as (1 = 50–55, 2 = 55–60, 3 = 60–65, 4 = 65–70, 5 = 70–75, 6 = 75+). Experimental factors included bisulphite conversion efficiency controls (BSC1 & BSC2), DNA input and batch number. P-values coded as follows: P<10e-10 (darkred), 10e-10<P<10e-5 (red), 10e-5<P<0.01 (orange), 0.01<P<0.05 (pink), P>0.05 (white). a) Before inter-array quantile normalisation, b) After inter-array quantile normalisation. c) After adjustment for BSC, DNA input and batch effects. (0.03 MB PDF) [file pone.0008274.s001.pdf]

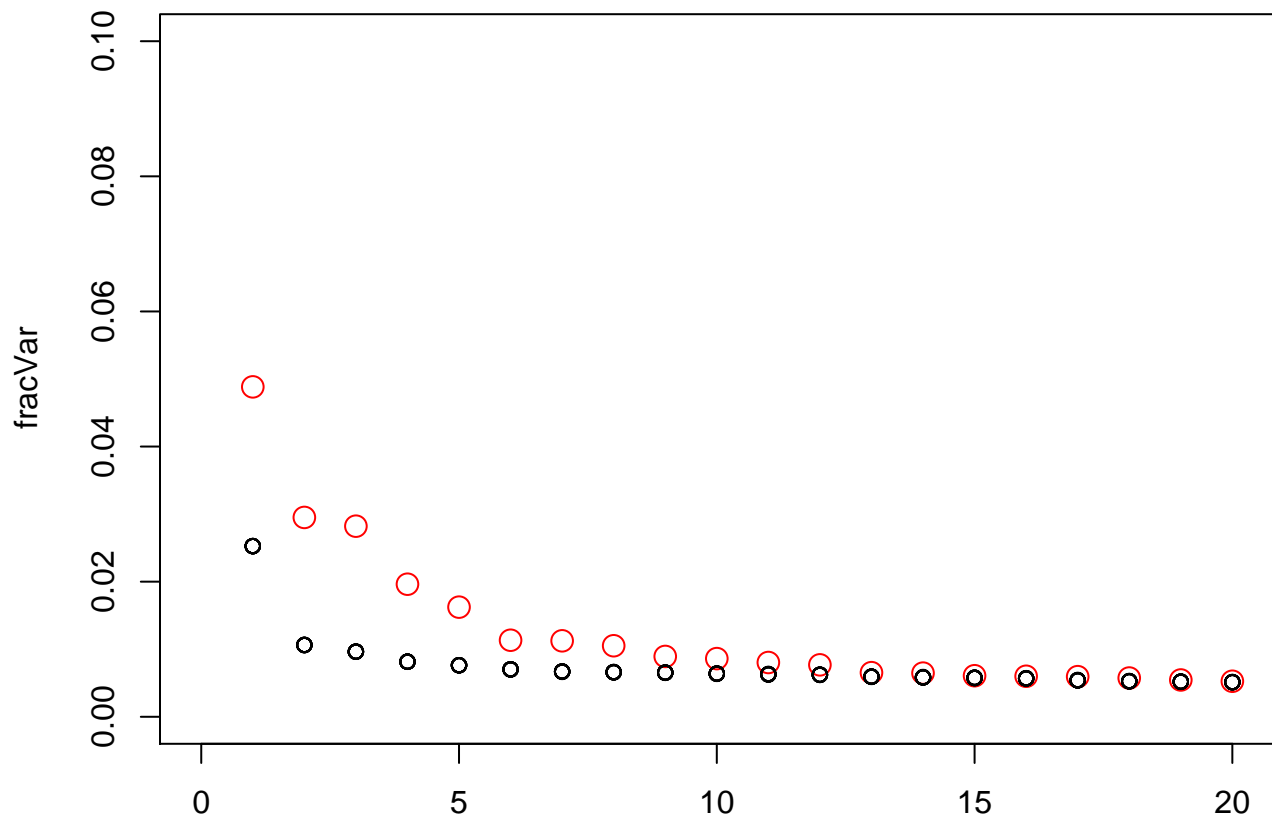

Supplement: Figure S2 — Significance analysis of singular values: Statistical significance analysis of singular values inferred from an SVD decomposition of the normalised adjusted data. x-axis denotes singular values ranked according to magnitude of variation. y-axis denotes the fraction of variation in the data explained by that singular value. Red points show the observed fractions, black points denote the fractions of variation under a random reshuffling of the data [Leek et al. 2008]. There are approximately 11 significant components of variation explaining about 24% of the variation in the data. (0.02 MB PDF) [file pone.0008274.s002.pdf]

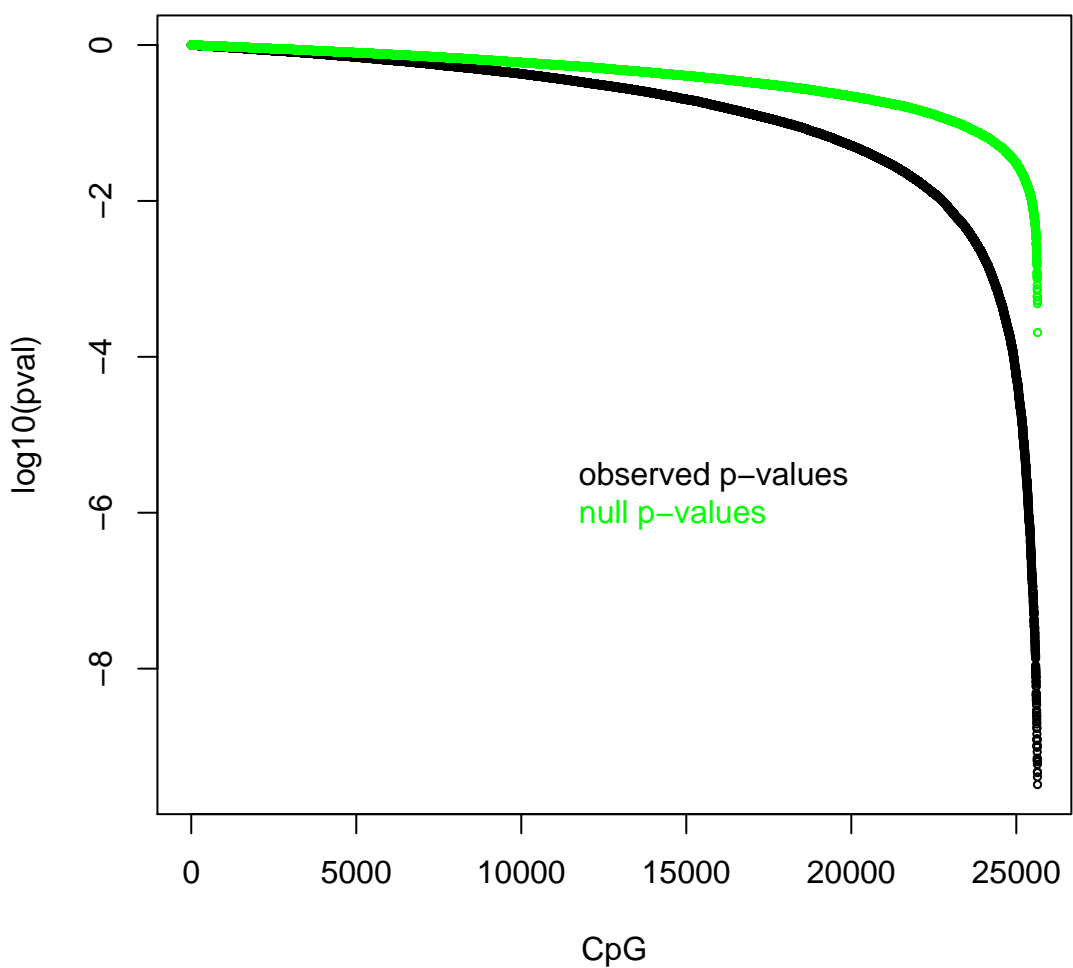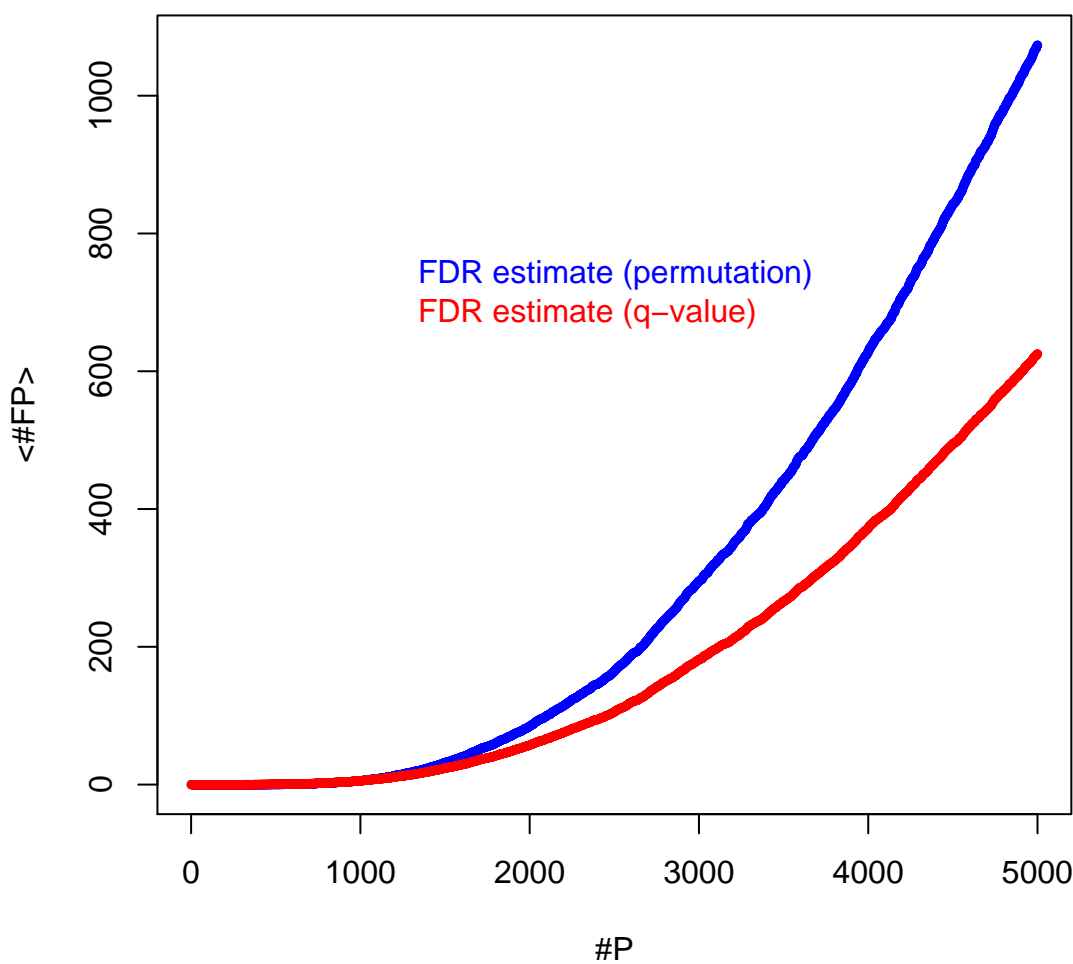

Supplement: Figure S3 — FDR estimation using permutation of sample labels: Top diagram plots the sorted log10(pvalues) (y-axis) of association with cancer (from logistic regression) against CpG index (x-axis). Black denotes observed p-values, green denotes corresponding values obtained after permutation of sample labels. Lower diagram compares the estimated mean number of false positives (y-axis) against the number of positives (x-axis) (i.e., the number of tests passing a given significance threshold). In blue, we show the estimate from the permutation approach; in red, the analytical estimate from the q-value. At an FDR∼0.05 both methods predict a similar number of significant CpGs. (3.86 MB PDF) [file pone.0008274.s003.pdf]

## Granulocyte markers

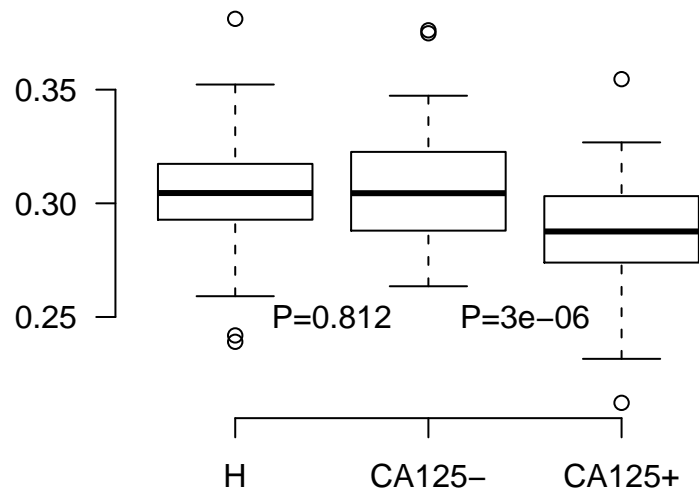

## Lymphocyte markers

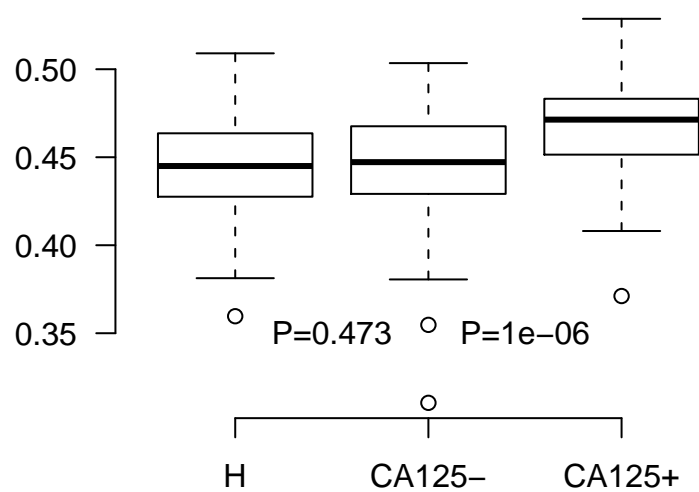

Supplement: Figure S4 — DNA methylation levels of granulocyte and lmphocyte markers: Average methylation levels (y-axis) of CpGs mapping to genes upregulated in granulocytes and lymphocytes against different disease states: H (healthy control samples, n = 148), CA125- (post-treatment cases with CA125<30, n = 70), CA125+ (post-treatment cases with CA125>30, n = 47). P-values from two-tailed Wilcoxon-tests between H and CA125- and between H and CA125+ are shown. (0.01 MB PDF) [file pone.0008274.s004.pdf]

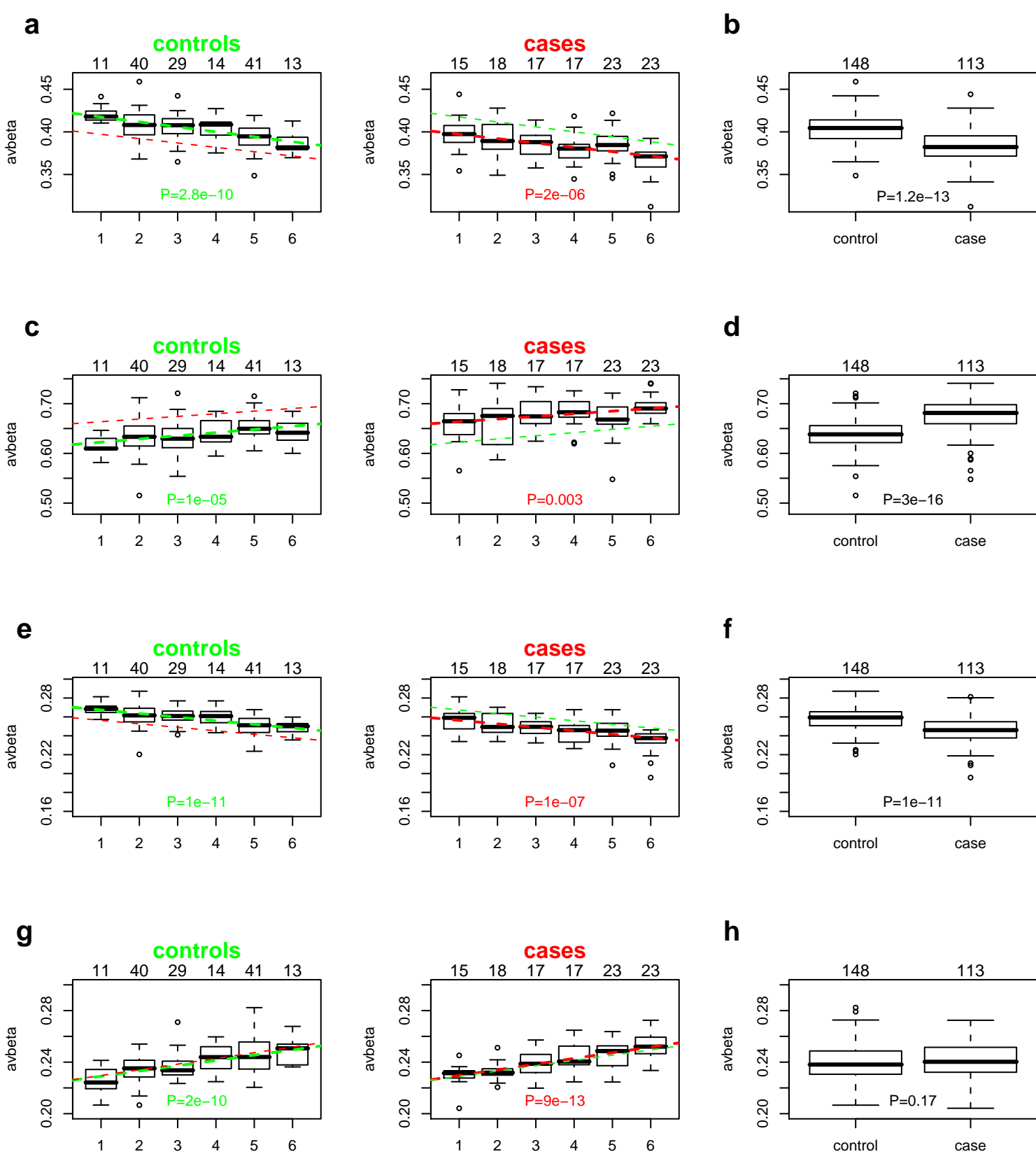

Supplement: Figure S5 — Age-dependent methylation patterns are associated with ovarian cancer: a–b) Average methylation patterns of age anti-correlated niCpGs selected through supervised analysis.(a) Average methylation versus age group for controls and cases. (b) Average methylation versus disease status. c–d) Average methylation patterns of age correlated niCpGs selected through supervised analysis.(c) Average methylation versus age group for controls and cases. (d) Average methylation versus disease status. e–f) Average methylation patterns of age anti-correlated iCpGs selected through supervised analysis.(e) Average methylation versus age group for controls and cases. (f) Average methylation versus disease status. g–h) Average methylation patterns of age correlated iCpGs selected through supervised analysis.(g) Average methylation versus age group for controls and cases. (h) Average methylation versus disease status. In panels b,d,f,h, p-values are from a two-tailed Wilcoxon rank sum test (0 = controls, 1 = case). In panels a,c,e,g, we give the numbers of samples in each age group and P-values reflect strength of the linear regression. Age groups are coded as (1 = 50 to 55, 2 = 55–60, 3 = 60–65, 4 = 65–70, 5 = 70–75, 6 = over75). Cases are pretreatment samples. (0.05 MB PDF) [file pone.0008274.s005.pdf]

a

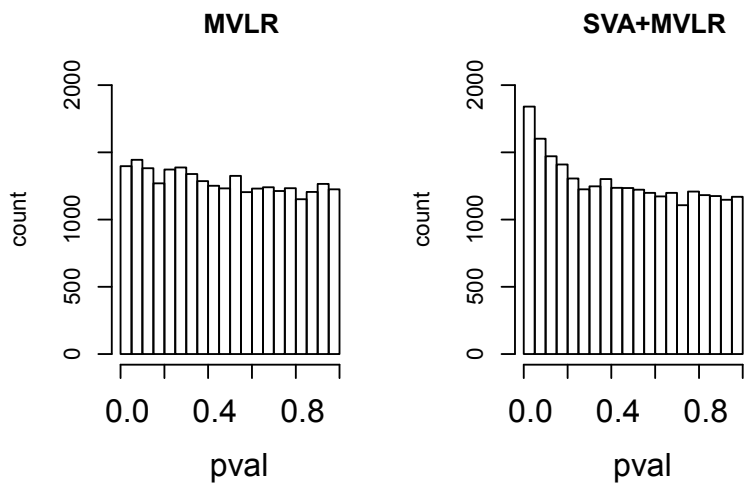

b

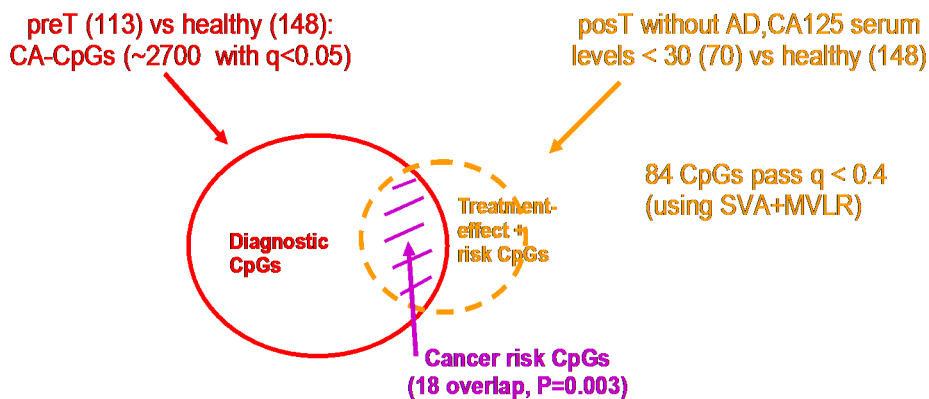

Supplement: Figure S6 — Derivation of cancer predisposition/risk CpGs: a) Histogram of p-values from multivariate logistic regression models (MVLR) comparing cancer status of postreatment patients without active disease at sample draw (70 samples) with age-matched healthy controls (148 samples). Logistic regression models included cancer status as a binary response and the CpG methylation profile as a predictor with batch, bisulphite conversion and DNA input as co-factors. Histogram distribution is relatively flat indicating the absence of discriminatory CpGs. Using Surrogate Variable Analysis (SVA+MVLR) to model all confounding known and hidden factors, p-value distribution exhibits a skew towards significant p-values, suggesting the existence of discriminatory CpGs. b) To deconvolute the effects of tumor-presence and treatment, cancer predisposition or risk CpGs should be given by the overlap of cancer-diagnostic CpGs with the 84 CpGs (FDR (q)<0.4) that discriminate postreatment cases without active disease (AD) from healthy controls. This yielded 18 candidate ovarian cancer risk CpGs. (0.08 MB PDF) [file pone.0008274.s006.pdf]
