# Supplementary material for: An Epigenetic Signature in Peripheral Blood Predicts Active Ovarian Cancer
Source: PLoS One. 2009 Dec 18;4(12):e8274. doi: 10.1371/journal.pone.0008274 (PMC2793425; doi:10.1371/journal.pone.0008274)
Supplement: Table S1 — Clinical characteristics of samples. (0.06 MB PDF) [file pone.0008274.s007.pdf]

Suppl Table 1

Characteristics		Controls		Pre-treatment Cases		versus controls		Post-treatment Cases		versus controls	
		n	%	*n*	%	OR (95%CI)	p-value	*n*	%	OR (95%CI)	p-value
**Age**	<65	149	54	55	42			68	50		
	>65	125	46	76	58	1.6 (1.1-2.5)	0.02	67	50	1.2 (0.8-1.8)	0.44
**Ethnicity**	White	247	90	105	80			116	86		
	Other	6	2	4	3	0.4-5.7	0.49	3	2	1.1 (0.3-4.3)	0.93
	Unknown	21	8	22	17			16	12		
**Age at menarche***	<13	156	57	36	27			67	50		
	>13	97	35	36	27	1.6 (0.9-2.7)	0.1	29	21	0.7 (0.4-1.2)	0.16
	Unknown	21	8	59	45			39	29		
**Ever pregnant**	No	5	2	18	14			18	13		
	Yes	230	84	53	40	0.1 (0.0-0.2)	<0.001	79	59	0.1 (0.0-0.3)	<0.001
	Unknown	39	14	60	46			38	28		
**Ever Oral Contraceptives used**	No	24	9	37	28			60	44		
	Yes	151	55	34	26	0.1 (0.1-0.3)	<0.001	36	27	0.1 (0.1-0.2)	<0.001
	Unknown	99	36	60	46			39	29		
**Ever Fertility Problems**	No	54	20	58	44			79	79		
	Yes	11	4	7	5	0.6 (0.2-1.6)	0.31	16	16	1.0 (0.4-2.3)	0.98
	Unknown	209	76	66	50			40	40		

**TUMOR CHARACTERISTICS**		**Pre-treatment**		**Post-treatment**		**P- value**
		n	**%***	***n***	**%***	
**Histology**	Serous	72	55	80	59	n.s.
	Mucinous	15	11	15	11	
	Endometric	18	14	19	14	
	Clear cell	14	11	14	10	
	Other	12	9	7	5	
**Stage**	I	49	37	44	33	n.s.
	II	13	10	20	15	
	III	58	44	62	46	
	IV	11	8	9	7	
**Grading**	1	17	13	10	7	n.s.
	2	32	24	32	24	
	3	70	53	71	53	
	Unknown	12	9	22	16	

**Age at recruitment (Mean +/- SD)**	67.2 (+/- 9.1)	65.6 (+/- 9.6)	n.s.
**Age at diagnosis (Mean +/- SD)**	67.2 (+/- 9.1)	63.2 (+/- 9.1)	0.001
