# Supplementary material for: An Epigenetic Signature in Peripheral Blood Predicts Active Ovarian Cancer
Source: PLoS One. 2009 Dec 18;4(12):e8274. doi: 10.1371/journal.pone.0008274 (PMC2793425; doi:10.1371/journal.pone.0008274)
Supplement: Table S2 — List of cancer diagnostic CpGs (CA-CpGs). (0.58 MB PDF) [file pone.0008274.s008.pdf]

IlmnID	Symbol	Entrez_ID	CPG_ISLAND	OR (95% CI)	P-val	Q-val	Correlation with Age
cg08044694	BRD4	23476	TRUE	0.26(0.17-0.39)	3E−10	1E−06	0
cg09134726	PRTN3	5657	FALSE	0.26(0.17-0.4)	4E−10	1E−06	0
cg20070090	S100A8	6279	FALSE	0.27(0.18-0.41)	5E−10	1E−06	0
cg00974864	FCGR3B	2215	FALSE	0.24(0.15-0.38)	5E−10	1E−06	0
cg01980222	TREM2	54209	FALSE	0.27(0.18-0.41)	6E−10	1E−06	−1
cg20748065	POR	5447	TRUE	0.23(0.14-0.37)	6E−10	1E−06	0
cg24211388	AIF1	199	FALSE	0.27(0.18-0.42)	7E−10	1E−06	0
cg02679745	FUT7	2529	FALSE	0.27(0.18-0.41)	7E−10	1E−06	0
cg27461196	FXYD1	5348	FALSE	0.26(0.17-0.4)	7E−10	1E−06	0
cg27606341	FYB	2533	FALSE	0.28(0.19-0.42)	9E−10	1E−06	0
cg18638581	HK2	3099	FALSE	0.24(0.15-0.38)	1E−09	1E−06	0
cg20792833	PTPRCAP	5790	FALSE	3.26(2.23-4.76)	1E−09	1E−06	0
cg25832796	AP1G2	8906	FALSE	0.29(0.19-0.43)	1E−09	1E−06	−1
cg24777950	CTSG	1511	FALSE	0.27(0.18-0.42)	1E−09	1E−06	−1
cg25634666	FOLR3	2352	FALSE	0.27(0.17-0.41)	1E−09	1E−06	0
cg03801286	KCNE1	3753	FALSE	0.28(0.18-0.42)	1E−09	1E−06	0
cg24898863	S100A8	6279	FALSE	0.28(0.19-0.43)	1E−09	1E−06	0
cg14654385	URP2	83706	TRUE	0.28(0.19-0.43)	1E−09	1E−06	0
cg02240622	PLCB2	5330	FALSE	0.26(0.17-0.41)	1E−09	1E−06	0
cg18390025	ELOVL3	83401	TRUE	0.27(0.18-0.42)	1E−09	1E−06	0
cg18920397	LY9	4063	FALSE	3.73(2.43-5.73)	2E−09	1E−06	1
cg09964921	KCNE1	3753	FALSE	0.29(0.2-0.44)	2E−09	1E−06	0
cg10287137	P2RY2	5029	TRUE	0.3(0.2-0.45)	2E−09	1E−06	0
cg15464148	GPR92	57121	FALSE	3.53(2.33-5.36)	2E−09	1E−06	0
cg11283860	SLC45A1	50651	TRUE	0.27(0.18-0.42)	2E−09	1E−06	0
cg17078393	LCK	3932	TRUE	3.32(2.23-4.94)	2E−09	2E−06	0

cg09303642	NFE2	4778	FALSE	0.28(0.18-0.43)	3E−09	2E−06	0
cg03330678	SEPT9	10801	TRUE	0.3(0.2-0.45)	3E−09	2E−06	0
cg12125117	GPR97	222487	FALSE	0.28(0.19-0.43)	3E−09	2E−06	−1
cg18084554	ARID3A	1820	FALSE	0.29(0.19-0.44)	3E−09	2E−06	0
cg25600606	HIPK3	10114	FALSE	0.32(0.22-0.47)	4E−09	2E−06	0
cg07376232	AMICA1	120425	FALSE	0.3(0.2-0.45)	4E−09	2E−06	0
cg00645579	IRF7	3665	TRUE	0.3(0.2-0.45)	4E−09	2E−06	0
cg02863947	NR1I2	8856	FALSE	0.31(0.21-0.46)	4E−09	2E−06	0
cg11822932	LMO2	4005	FALSE	0.3(0.2-0.45)	4E−09	2E−06	0
cg07285167	CSF3R	1441	FALSE	0.29(0.2-0.44)	4E−09	2E−06	−1
cg04353769	MS4A6A	64231	FALSE	0.29(0.19-0.44)	5E−09	2E−06	0
cg22820108	NCOR2	9612	TRUE	0.28(0.18-0.43)	5E−09	2E−06	0
cg23090046	KNS2	3831	TRUE	0.32(0.21-0.47)	5E−09	2E−06	0
cg10161121	FASLG	356	FALSE	3.11(2.12-4.56)	5E−09	2E−06	0
cg27485921	ATP6V1E2	90423	FALSE	0.29(0.19-0.45)	6E−09	2E−06	0
cg15910079	RNASE3	6037	FALSE	0.31(0.21-0.46)	6E−09	2E−06	−1
cg12640109	ASRGL1	80150	TRUE	0.34(0.24-0.49)	6E−09	2E−06	0
cg24427660	PNPLA2	57104	TRUE	0.29(0.19-0.45)	7E−09	2E−06	−1
cg08368934	GPR97	222487	FALSE	0.3(0.2-0.45)	7E−09	2E−06	0
cg24949488	DNTT	1791	TRUE	3.25(2.18-4.86)	8E−09	3E−06	1
cg17823175	AZU1	566	TRUE	0.31(0.21-0.47)	8E−09	3E−06	0
cg21991396	CIAS1	114548	FALSE	0.33(0.22-0.48)	8E−09	3E−06	0
cg11024597	ECRG4	84417	FALSE	0.31(0.21-0.47)	8E−09	3E−06	0
cg15784615	LTBR	4055	FALSE	0.32(0.21-0.47)	8E−09	3E−06	−1
cg17813891	EVL	51466	FALSE	3.25(2.17-4.86)	9E−09	3E−06	0
cg08458487	SFTPD	6441	FALSE	0.33(0.22-0.48)	9E−09	3E−06	0
cg08539991	ZBTB32	27033	FALSE	3.17(2.13-4.71)	9E−09	3E−06	0

cg22467534	UNC13D	201294	TRUE	0.32(0.21-0.47)	1E−08	3E−06	0
cg02374486	PRF1	5551	FALSE	3.04(2.08-4.46)	1E−08	3E−06	0
cg07730301	ALDH3B1	221	FALSE	0.28(0.18-0.43)	1E−08	3E−06	0
cg16361890	LTC4S	4056	FALSE	0.31(0.21-0.47)	1E−08	3E−06	0
cg13765961	MS4A1	931	FALSE	3.03(2.07-4.44)	1E−08	3E−06	0
cg14511156	OSCAR	126014	FALSE	0.31(0.21-0.47)	1E−08	4E−06	0
cg00071250	FASLG	356	FALSE	2.97(2.03-4.34)	1E−08	4E−06	0
cg06812844	TRPM2	7226	FALSE	0.32(0.21-0.47)	1E−08	4E−06	0
cg17356733	IFNGR2	3460	TRUE	0.32(0.22-0.48)	2E−08	4E−06	0
cg25050026	MFSD1	64747	TRUE	0.33(0.22-0.48)	2E−08	4E−06	0
cg21126943	CEACAM6	4680	FALSE	0.34(0.23-0.5)	2E−08	4E−06	0
cg19906550	SLC22A18	5002	FALSE	0.31(0.21-0.47)	2E−08	4E−06	0
cg08176694	PITPNM2	57605	TRUE	0.32(0.22-0.48)	2E−08	4E−06	0
cg05546038	NOL3	8996	FALSE	0.34(0.23-0.5)	2E−08	4E−06	0
cg25587233	PPP2R4	5524	FALSE	0.33(0.22-0.48)	2E−08	4E−06	0
cg17709873	LTA	4049	FALSE	3.26(2.16-4.94)	2E−08	4E−06	0
cg00333528	GABRR1	2569	FALSE	0.33(0.22-0.49)	2E−08	4E−06	0
cg22242539	SERPINF1	5176	TRUE	0.33(0.23-0.49)	2E−08	4E−06	0
cg05564251	SP140	11262	FALSE	3.12(2.09-4.66)	2E−08	4E−06	0
cg09358725	LMO2	4005	FALSE	0.31(0.21-0.47)	2E−08	4E−06	0
cg04988978	MPO	4353	FALSE	0.33(0.22-0.49)	2E−08	4E−06	−1
cg13053608	LGP1	84514	FALSE	0.34(0.23-0.49)	2E−08	5E−06	−1
cg06759890	LHFPL2	10184	FALSE	0.34(0.24-0.5)	2E−08	5E−06	−1
cg26701826	MGC26963	166929	FALSE	0.32(0.21-0.48)	2E−08	5E−06	0
cg02266731	CPM	1368	FALSE	0.33(0.23-0.49)	2E−08	5E−06	0
cg09868035	C20orf135	140701	FALSE	0.32(0.21-0.48)	2E−08	5E−06	0
cg25839227	ABI3	51225	FALSE	3.1(2.08-4.62)	3E−08	5E−06	0

cg18463686	CLEC5A	23601	FALSE	0.34(0.23-0.5)	3E−08	5E−06	0
cg01402255	GATAD2B	57459	FALSE	0.34(0.23-0.49)	3E−08	5E−06	−1
cg20320468	LAIR1	3903	FALSE	0.35(0.24-0.51)	3E−08	5E−06	0
cg02600394	TXK	7294	FALSE	2.95(2.01-4.31)	3E−08	5E−06	1
cg10257049	C5orf4	10826	FALSE	0.32(0.21-0.48)	3E−08	6E−06	0
cg09914304	PRF1	5551	FALSE	3(2.03-4.43)	3E−08	6E−06	1
cg13765621	CD1D	912	FALSE	0.35(0.24-0.51)	3E−08	6E−06	−1
cg01441777	CSNK1E	1454	TRUE	0.31(0.21-0.47)	3E−08	6E−06	−1
cg17173423	MS4A3	932	FALSE	0.32(0.21-0.48)	3E−08	6E−06	0
cg00134539	UBASH3A	53347	FALSE	2.6(1.85-3.65)	3E−08	6E−06	0
cg26154999	FLJ22746	79843	TRUE	0.3(0.19-0.46)	3E−08	6E−06	−1
cg25087423	BLR1	643	TRUE	3.07(2.06-4.59)	3E−08	6E−06	1
cg16967583	AGXT	189	FALSE	0.35(0.24-0.51)	3E−08	6E−06	0
cg05501357	HIPK3	10114	FALSE	0.31(0.2-0.47)	3E−08	6E−06	−1
cg21969640	GPR84	53831	FALSE	0.33(0.22-0.49)	3E−08	6E−06	−1
cg15374234	CD300LF	146722	FALSE	0.36(0.25-0.52)	4E−08	6E−06	0
cg17496921	TSPAN16	26526	FALSE	0.32(0.21-0.48)	4E−08	6E−06	−1
cg01774645	ARHGAP30	257106	FALSE	2.86(1.96-4.15)	4E−08	6E−06	0
cg11939496	CD244	51744	FALSE	0.37(0.26-0.53)	4E−08	6E−06	0
cg04759756	SLA2	84174	FALSE	2.88(1.97-4.22)	4E−08	6E−06	0
cg21932814	CSTA	1475	FALSE	0.36(0.25-0.52)	4E−08	6E−06	0
cg04756629	LOC400696	400696	FALSE	2.79(1.93-4.04)	4E−08	6E−06	0
cg01623438	CTSZ	1522	FALSE	0.33(0.22-0.49)	4E−08	6E−06	0
cg01636591	CCL8	6355	FALSE	0.37(0.26-0.53)	4E−08	6E−06	0
cg12022621	LAX1	54900	FALSE	2.88(1.97-4.21)	4E−08	6E−06	0
cg09305224	FUT7	2529	FALSE	0.37(0.26-0.53)	4E−08	7E−06	0
cg01040850	MR1	3140	FALSE	2.79(1.93-4.05)	4E−08	7E−06	0

cg06172871	HP	3240	FALSE	0.34(0.23-0.51)	5E−08	7E−06	0
cg07728874	CD3D	915	FALSE	2.96(2-4.38)	5E−08	7E−06	1
cg17839611	GNGT2	2793	FALSE	3.07(2.05-4.62)	5E−08	8E−06	0
cg15518883	SIT1	27240	FALSE	2.91(1.98-4.28)	5E−08	8E−06	1
cg07408456	PGLYRP2	114770	FALSE	0.35(0.24-0.51)	5E−08	8E−06	−1
cg12089698	SPATC1	375686	TRUE	0.36(0.25-0.52)	5E−08	8E−06	0
cg04451770	ENTPD1	953	FALSE	0.32(0.21-0.48)	5E−08	8E−06	0
cg06791867	TSPAN18	90139	FALSE	0.34(0.23-0.5)	5E−08	8E−06	0
cg17749456	HSPBP1	23640	FALSE	0.36(0.24-0.52)	6E−08	8E−06	−1
cg03574571	CD22	933	FALSE	0.36(0.25-0.52)	6E−08	8E−06	0
cg23547429	SLC43A3	29015	FALSE	0.35(0.24-0.52)	6E−08	8E−06	−1
cg09088576	CSF3R	1441	TRUE	0.34(0.23-0.51)	6E−08	8E−06	0
cg08399444	GSG1	83445	FALSE	0.36(0.25-0.52)	6E−08	8E−06	0
cg24612198	CD3E	916	FALSE	2.84(1.94-4.15)	6E−08	8E−06	0
cg16139316	S100A9	6280	FALSE	0.34(0.23-0.51)	6E−08	9E−06	−1
cg16872071	RALGDS	5900	FALSE	0.37(0.26-0.53)	7E−08	9E−06	0
cg15958424	ACPP	55	FALSE	0.35(0.24-0.52)	7E−08	9E−06	0
cg07239938	ELA2	1991	TRUE	0.33(0.22-0.49)	7E−08	9E−06	−1
cg02635407	SH3TC1	54436	TRUE	0.34(0.23-0.51)	7E−08	9E−06	0
cg09208010	MMP14	4323	TRUE	0.34(0.23-0.5)	7E−08	9E−06	0
cg22319147	CDH5	1003	FALSE	0.37(0.26-0.53)	7E−08	9E−06	0
cg07218880	UPF3A	65110	TRUE	2.71(1.88-3.91)	8E−08	9E−06	0
cg24474182	P2RY13	53829	FALSE	0.36(0.25-0.53)	8E−08	1E−05	0
cg12380764	IL19	29949	FALSE	0.34(0.23-0.51)	8E−08	1E−05	0
cg25226014	CXCR6	10663	FALSE	2.71(1.88-3.91)	8E−08	1E−05	1
cg10275770	ICAM2	3384	FALSE	0.34(0.23-0.5)	9E−08	1E−05	0
cg08700306	LRP3	4037	FALSE	0.36(0.25-0.53)	9E−08	1E−05	0

cg11898695	PTCRA	171558	FALSE	3.08(2.04-4.65)	9E−08	1E−05	0
cg01525376	LCK	3932	FALSE	2.63(1.84-3.75)	9E−08	1E−05	0
cg01526089	P2RX1	5023	TRUE	0.34(0.23-0.51)	9E−08	1E−05	0
cg00328227	C1orf59	113802	TRUE	2.71(1.87-3.91)	9E−08	1E−05	0
cg20340242	IL1R2	7850	TRUE	0.32(0.21-0.49)	9E−08	1E−05	0
cg01367992	LY9	4063	FALSE	2.76(1.9-4.01)	9E−08	1E−05	1
cg22016649	PNPLA2	57104	TRUE	0.36(0.25-0.53)	1E−07	1E−05	−1
cg26206598	PREX1	57580	FALSE	0.37(0.25-0.53)	1E−07	1E−05	0
cg04653308	PPP2R4	5524	FALSE	0.34(0.23-0.51)	1E−07	1E−05	0
cg24489015	LPO	4025	FALSE	0.34(0.23-0.51)	1E−07	1E−05	0
cg14088811	SPI1	6688	TRUE	0.34(0.23-0.51)	1E−07	1E−05	0
cg22854223	CD82	3732	TRUE	0.37(0.25-0.53)	1E−07	1E−05	0
cg11098259	AQP9	366	FALSE	0.36(0.25-0.53)	1E−07	1E−05	0
cg15528736	FCGRT	2217	TRUE	0.38(0.27-0.55)	1E−07	1E−05	0
cg02595219	KCNE3	10008	TRUE	0.38(0.26-0.54)	1E−07	1E−05	0
cg06625767	F12	2161	FALSE	0.37(0.26-0.54)	1E−07	1E−05	0
cg19812619	ITGB7	3695	FALSE	2.72(1.88-3.95)	1E−07	1E−05	1
cg06653796	LIME1	54923	TRUE	2.7(1.87-3.92)	1E−07	1E−05	0
cg09624565	NCF4	4689	FALSE	0.35(0.24-0.52)	1E−07	1E−05	0
cg10057295	STK24	8428	TRUE	0.37(0.25-0.53)	1E−07	1E−05	−1
cg22088368	MGC35206	339669	FALSE	0.37(0.26-0.54)	1E−07	1E−05	0
cg01617750	CMTM8	152189	TRUE	0.39(0.28-0.56)	1E−07	1E−05	0
cg22799850	FBXL13	222235	TRUE	0.38(0.26-0.54)	1E−07	1E−05	−1
cg25341726	IL27	246778	FALSE	0.38(0.27-0.55)	1E−07	1E−05	0
cg00899659	ZNF22	7570	FALSE	0.35(0.23-0.52)	1E−07	1E−05	0
cg21019522	SLC22A18	5002	FALSE	0.35(0.24-0.52)	1E−07	1E−05	0
cg24453664	CD59	966	FALSE	0.37(0.25-0.54)	1E−07	1E−05	0

cg06688396	TMEM55A	55529	FALSE	0.36(0.25-0.53)	1E−07	1E−05	0
cg24091474	TYROBP	7305	FALSE	0.36(0.24-0.52)	1E−07	1E−05	0
cg22045288	C10orf91	170393	FALSE	0.38(0.26-0.54)	1E−07	1E−05	0
cg09037813	LRRFIP1	9208	TRUE	0.39(0.27-0.56)	1E−07	1E−05	0
cg10061138	STAB1	23166	FALSE	0.34(0.23-0.51)	1E−07	1E−05	0
cg02473123	CD7	924	TRUE	2.63(1.83-3.78)	2E−07	1E−05	0
cg21917349	APBA2	321	FALSE	2.73(1.87-3.98)	2E−07	1E−05	0
cg06196379	TREM1	54210	FALSE	0.36(0.25-0.53)	2E−07	1E−05	−1
cg20713492	AQP10	89872	FALSE	0.37(0.26-0.54)	2E−07	1E−05	0
cg12044210	APBA2	321	FALSE	2.61(1.82-3.74)	2E−07	2E−05	0
cg19464944	FCGR1A	2209	FALSE	0.36(0.25-0.53)	2E−07	2E−05	0
cg02181506	SERPINA1	5265	FALSE	0.35(0.24-0.52)	2E−07	2E−05	0
cg17296078	UBTD1	80019	TRUE	0.37(0.25-0.54)	2E−07	2E−05	0
cg09626634	EBI2	1880	FALSE	2.6(1.81-3.74)	2E−07	2E−05	0
cg03548857	FFAR2	2867	FALSE	0.39(0.27-0.55)	2E−07	2E−05	0
cg05055150	MAG	4099	FALSE	2.81(1.9-4.15)	2E−07	2E−05	0
cg06849477	PRR5	55615	FALSE	0.37(0.25-0.54)	2E−07	2E−05	0
cg06270401	DYRK4	8798	FALSE	0.33(0.21-0.5)	2E−07	2E−05	0
cg05795313	ZNF641	121274	TRUE	0.38(0.27-0.55)	2E−07	2E−05	0
cg27285056	NAPSA	9476	FALSE	2.78(1.89-4.09)	2E−07	2E−05	0
cg17186163	C10orf10	11067	FALSE	0.37(0.26-0.54)	2E−07	2E−05	0
cg15337006	ITGAM	3684	FALSE	0.37(0.26-0.54)	2E−07	2E−05	0
cg24841244	CD3D	915	FALSE	2.66(1.83-3.84)	2E−07	2E−05	1
cg02516189	CARD9	64170	FALSE	0.36(0.25-0.53)	2E−07	2E−05	−1
cg06806711	MS4A1	931	FALSE	2.52(1.77-3.58)	2E−07	2E−05	0
cg15512851	FGD2	221472	FALSE	0.36(0.25-0.53)	2E−07	2E−05	0
cg15880738	CD3G	917	FALSE	2.61(1.81-3.76)	3E−07	2E−05	1

cg03311899	GPR109A	338442	TRUE	0.4(0.29-0.57)	3E−07	2E−05	0
cg15125472	HSPB6	126393	TRUE	0.37(0.25-0.54)	3E−07	2E−05	0
cg04784315	GPR21	2844	FALSE	0.37(0.25-0.54)	3E−07	2E−05	0
cg26757673	IL2RB	3560	FALSE	2.53(1.77-3.62)	3E−07	2E−05	0
cg13634319	DGKA	1606	FALSE	2.55(1.78-3.65)	3E−07	2E−05	0
cg04164824	SIGLEC9	27180	FALSE	0.36(0.25-0.54)	3E−07	2E−05	0
cg20764656	GPX2	2877	FALSE	0.39(0.27-0.56)	3E−07	2E−05	0
cg17001035	PCGF5	84333	TRUE	0.39(0.27-0.56)	3E−07	2E−05	−1
cg07759587	TLR8	51311	FALSE	0.39(0.28-0.56)	3E−07	2E−05	0
cg18741908	GPR160	26996	TRUE	0.37(0.25-0.54)	3E−07	2E−05	0
cg10126923	NKG7	4818	FALSE	0.4(0.28-0.57)	3E−07	2E−05	−1
cg13301014	SPINT2	10653	TRUE	0.39(0.27-0.56)	3E−07	2E−05	0
cg12971694	CD72	971	FALSE	0.38(0.26-0.55)	3E−07	2E−05	0
cg15227982	C10orf26	54838	FALSE	0.37(0.25-0.54)	3E−07	3E−05	0
cg23140706	NFE2	4778	FALSE	0.4(0.28-0.57)	3E−07	3E−05	0
cg11384427	TNFRSF7	939	FALSE	2.96(1.95-4.48)	3E−07	3E−05	1
cg21578541	TLR9	54106	FALSE	0.41(0.29-0.57)	3E−07	3E−05	0
cg10129493	CD33	945	FALSE	0.38(0.27-0.56)	3E−07	3E−05	0
cg13578652	UBASH3A	53347	FALSE	2.41(1.71-3.38)	3E−07	3E−05	0
cg23221013	MGC2463	79037	FALSE	2.48(1.74-3.52)	4E−07	3E−05	0
cg21400896	ABI3	51225	FALSE	2.58(1.79-3.72)	4E−07	3E−05	0
cg22681784	SPINK2	6691	TRUE	0.38(0.26-0.55)	4E−07	3E−05	0
cg15352315	CD302	9936	FALSE	0.4(0.28-0.57)	4E−07	3E−05	0
cg22442090	GIMAP5	55340	FALSE	2.57(1.78-3.72)	4E−07	3E−05	0
cg26112639	CIAS1	114548	FALSE	0.4(0.28-0.57)	4E−07	3E−05	0
cg22381196	DHODH	1723	TRUE	0.4(0.28-0.57)	4E−07	3E−05	0
cg19491035	MANSC1	54682	TRUE	0.4(0.28-0.57)	4E−07	3E−05	0

cg08223235	BCL2	596	TRUE	0.4(0.28-0.57)	4E−07	3E−05	0
cg19700658	UCP3	7352	FALSE	2.75(1.85-4.08)	4E−07	3E−05	0
cg26215727	SCNN1A	6337	FALSE	0.39(0.27-0.57)	4E−07	3E−05	0
cg19154438	CKM	1158	FALSE	2.61(1.79-3.8)	5E−07	4E−05	1
cg03886110	PECAM1	5175	TRUE	0.39(0.27-0.56)	5E−07	4E−05	−1
cg19399532	FLJ35530	400798	TRUE	0.41(0.29-0.58)	5E−07	4E−05	0
cg24643262	BMX	660	TRUE	0.38(0.26-0.55)	5E−07	4E−05	0
cg22680812	EPB42	2038	FALSE	2.75(1.85-4.09)	5E−07	4E−05	0
cg13471990	ENTPD1	953	FALSE	0.39(0.27-0.57)	5E−07	4E−05	0
cg19531130	ANGPTL5	253935	FALSE	0.41(0.29-0.59)	5E−07	4E−05	0
cg10266490	ACOT11	26027	FALSE	0.39(0.27-0.57)	6E−07	4E−05	−1
cg23265096	CTSZ	1522	TRUE	0.39(0.27-0.56)	6E−07	4E−05	0
cg00718513		28905	FALSE	2.48(1.73-3.56)	6E−07	4E−05	0
cg15248035	CCIN	881	TRUE	0.4(0.28-0.58)	6E−07	4E−05	0
cg08766149	GZMB	3002	FALSE	2.57(1.78-3.73)	6E−07	4E−05	0
cg02225847	FAM65A	79567	TRUE	0.4(0.28-0.57)	6E−07	4E−05	0
cg01578324	CLIC1	1192	FALSE	0.39(0.27-0.57)	6E−07	4E−05	0
cg18752880	C1QTNF3	114899	FALSE	0.41(0.28-0.58)	6E−07	4E−05	−1
cg09025324	SART2	29940	TRUE	0.41(0.29-0.58)	6E−07	4E−05	0
cg02039171	CEBPE	1053	TRUE	0.4(0.28-0.57)	6E−07	4E−05	0
cg20654468	LPXN	9404	FALSE	0.37(0.25-0.55)	6E−07	4E−05	0
cg16028753	PODN	127435	TRUE	0.41(0.29-0.58)	7E−07	4E−05	0
cg09830866	C16orf24	65990	TRUE	2.48(1.73-3.55)	7E−07	5E−05	0
cg22224704	GSTP1	2950	TRUE	0.42(0.3-0.59)	7E−07	5E−05	−1
cg00415993	F2RL2	2151	FALSE	0.4(0.28-0.58)	7E−07	5E−05	0
cg06147863	SPI1	6688	FALSE	0.39(0.27-0.56)	7E−07	5E−05	0
cg24323726	ZBED2	79413	FALSE	2.6(1.78-3.8)	7E−07	5E−05	0

cg07924575	HPS4	89781	FALSE	0.4(0.28-0.57)	7E−07	5E−05	0
cg19843036	FLJ11155	55314	TRUE	0.35(0.23-0.53)	7E−07	5E−05	0
cg22266967	S100P	6286	FALSE	0.41(0.28-0.58)	7E−07	5E−05	0
cg07086380	TNFAIP8	25816	TRUE	2.38(1.68-3.36)	7E−07	5E−05	0
cg17740645	GRB7	2886	FALSE	0.41(0.29-0.59)	7E−07	5E−05	0
cg15146752	EPHA2	1969	TRUE	0.42(0.3-0.6)	8E−07	5E−05	0
cg16465939	KCNQ1	3784	TRUE	0.41(0.29-0.59)	8E−07	5E−05	0
cg09001777	FUT3	2525	FALSE	0.42(0.29-0.59)	8E−07	5E−05	0
cg00795812	PDCD1	5133	TRUE	2.6(1.77-3.8)	8E−07	5E−05	0
cg04790874	CD79A	973	FALSE	0.41(0.29-0.59)	8E−07	5E−05	0
cg09499849	ACVR1	90	FALSE	0.42(0.3-0.59)	8E−07	5E−05	0
cg16692277	GUCY1B2	2974	TRUE	0.4(0.28-0.58)	8E−07	5E−05	−1
cg10548978	C10orf27	219793	FALSE	0.39(0.27-0.57)	8E−07	5E−05	0
cg03856723	PRKACA	5566	FALSE	0.37(0.25-0.55)	8E−07	5E−05	0
cg20777437	CDCP2	200008	FALSE	0.41(0.29-0.59)	8E−07	5E−05	0
cg08260891	PPGB	5476	TRUE	0.41(0.29-0.59)	8E−07	5E−05	0
cg17105014	GYPC	2995	FALSE	0.4(0.27-0.57)	8E−07	5E−05	−1
cg25957124	DNAH3	55567	TRUE	0.41(0.29-0.59)	8E−07	5E−05	0
cg03608974	ZAK	51776	TRUE	0.41(0.29-0.59)	9E−07	5E−05	0
cg14324675	LST1	7940	FALSE	0.39(0.27-0.57)	9E−07	5E−05	0
cg19963522	PIP3-E	26034	FALSE	0.42(0.3-0.6)	9E−07	5E−05	0
cg26191951	RNASE2	6036	FALSE	0.4(0.28-0.58)	9E−07	5E−05	0
cg20981615	TXK	7294	FALSE	2.37(1.67-3.35)	9E−07	5E−05	1
cg02656594	IL21R	50615	FALSE	0.41(0.28-0.59)	9E−07	5E−05	0
cg10148841	ROBO4	54538	FALSE	0.42(0.3-0.59)	9E−07	6E−05	0
cg08203715	ST3GAL4	6484	TRUE	0.39(0.27-0.57)	9E−07	6E−05	0
cg12535715	HTRA4	203100	TRUE	0.37(0.25-0.56)	9E−07	6E−05	0

cg20720686	POR	5447	FALSE	0.4(0.28-0.58)	1E−06	6E−05	0
cg20651453	MAP3K6	9064	TRUE	0.41(0.29-0.59)	1E−06	6E−05	0
cg25432696	CXCL6	6372	TRUE	0.41(0.29-0.59)	1E−06	6E−05	0
cg24292612	DEFB1	1672	FALSE	0.44(0.32-0.61)	1E−06	6E−05	0
cg07525077	RNASE3	6037	FALSE	0.41(0.29-0.59)	1E−06	7E−05	0
cg21846903	VTN	7448	FALSE	0.42(0.29-0.59)	1E−06	7E−05	0
cg17122311	IL27	246778	FALSE	0.43(0.3-0.6)	1E−06	7E−05	0
cg05106502	SCAP1	8631	FALSE	2.31(1.64-3.24)	1E−06	7E−05	0
cg00546897	LOC284837	284837	FALSE	0.41(0.28-0.59)	1E−06	7E−05	−1
cg11657615	ZDHHC1	29800	TRUE	0.37(0.24-0.55)	1E−06	7E−05	0
cg11827101	LOC339789	339789	FALSE	0.42(0.29-0.59)	1E−06	7E−05	0
cg12836863	BRCA2	675	TRUE	0.42(0.29-0.59)	1E−06	7E−05	−1
cg12045829	TNFSF12	8742	TRUE	2.26(1.62-3.15)	1E−06	7E−05	0
cg22566906	GRASP	160622	FALSE	0.41(0.28-0.59)	1E−06	7E−05	0
cg26660631	FLJ32011	148930	FALSE	2.38(1.67-3.4)	1E−06	8E−05	0
cg24256211	SAMD4A	23034	TRUE	0.41(0.29-0.59)	1E−06	8E−05	0
cg20674521	KCNJ4	3761	FALSE	2.41(1.69-3.43)	1E−06	8E−05	0
cg04502814	SEPP1	6414	FALSE	0.41(0.29-0.59)	1E−06	8E−05	0
cg05044994	FLJ42393	401105	FALSE	2.39(1.68-3.42)	1E−06	8E−05	0
cg26823505	CLPS	1208	FALSE	2.35(1.66-3.33)	1E−06	8E−05	0
cg17714799	CASP6	839	FALSE	0.42(0.3-0.6)	1E−06	8E−05	0
cg02332073	TSGA13	114960	FALSE	0.43(0.3-0.61)	2E−06	8E−05	0
cg16097772	LYZ	4069	FALSE	0.41(0.29-0.59)	2E−06	8E−05	0
cg24926276	LRG1	116844	FALSE	0.4(0.28-0.58)	2E−06	9E−05	0
cg15536230	H2BFS	54145	TRUE	0.44(0.31-0.62)	2E−06	9E−05	0
cg01718139	UNQ3033	284415	TRUE	0.39(0.27-0.57)	2E−06	9E−05	0
cg05037688	EGFL7	51162	FALSE	0.4(0.27-0.58)	2E−06	9E−05	−1

cg02275294	SOAT1	6646	TRUE	0.42(0.3-0.6)	2E−06	9E−05	−1
cg01861509	SPOCK2	9806	FALSE	2.32(1.64-3.28)	2E−06	9E−05	1
cg09418321	DYRK4	8798	FALSE	0.42(0.29-0.6)	2E−06	9E−05	0
cg15743985	CD22	933	TRUE	0.43(0.3-0.61)	2E−06	9E−05	0
cg02540157	MGC2463	79037	FALSE	2.42(1.68-3.48)	2E−06	0.0001	0
cg05461841	LOC124220	124220	FALSE	0.44(0.31-0.62)	2E−06	0.0001	0
cg13703437	FYB	2533	FALSE	0.43(0.3-0.61)	2E−06	0.0001	0
cg02212836	LY86	9450	FALSE	2.43(1.68-3.5)	2E−06	0.0001	0
cg10117369	LAX1	54900	FALSE	2.42(1.68-3.48)	2E−06	0.0001	0
cg00234961	ZBED4	9889	TRUE	0.43(0.3-0.61)	2E−06	0.0001	0
cg15121304		3535	FALSE	2.73(1.8-4.14)	2E−06	0.0001	1
cg26728422	C16orf28	65259	TRUE	0.42(0.29-0.6)	2E−06	0.0001	0
cg15674432	SLC26A8	116369	TRUE	0.41(0.28-0.59)	2E−06	0.0001	0
cg21480743	PTEN	5728	TRUE	0.43(0.3-0.61)	2E−06	0.0001	0
cg21902327	FGF6	2251	TRUE	2.24(1.6-3.12)	2E−06	0.0001	0
cg11494699	RAG1	5896	FALSE	3.02(1.91-4.79)	2E−06	0.0001	0
cg18096388	PDCD1	5133	FALSE	2.31(1.63-3.28)	2E−06	0.0001	0
cg18959422	MYBPH	4608	FALSE	2.27(1.62-3.19)	2E−06	0.0001	0
cg25028542	ANLN	54443	TRUE	0.43(0.31-0.62)	2E−06	0.0001	−1
cg06154570	HEYL	26508	TRUE	0.44(0.31-0.62)	2E−06	0.0001	0
cg00269932	LAIR2	3904	FALSE	0.44(0.31-0.62)	2E−06	0.0001	0
cg15055101	SH2D3A	10045	TRUE	2.35(1.64-3.36)	2E−06	0.0001	1
cg08861115	IL1F9	56300	FALSE	2.35(1.64-3.36)	2E−06	0.0001	0
cg23228178	PADI4	23569	FALSE	2.28(1.62-3.23)	2E−06	0.0001	0
cg08351331	LBP	3929	FALSE	2.29(1.62-3.24)	2E−06	0.0001	0
cg26861460	PARVG	64098	FALSE	0.43(0.3-0.61)	2E−06	0.0001	0
cg13650156	PILRA	29992	FALSE	0.43(0.3-0.61)	3E−06	0.0001	0

cg21237418	RAB34	83871	TRUE	0.43(0.3-0.61)	3E−06	0.0001	0
cg18333690	PADI4	23569	FALSE	0.44(0.31-0.62)	3E−06	0.0001	0
cg02082571	CLEC4A	50856	FALSE	0.39(0.26-0.58)	3E−06	0.0001	0
cg04616963	XCL1	6375	FALSE	2.31(1.63-3.29)	3E−06	0.0001	1
cg05868799	NCOA2	10499	TRUE	0.41(0.28-0.6)	3E−06	0.0001	0
cg10236239	SULT1C2	27233	FALSE	0.44(0.31-0.62)	3E−06	0.0001	−1
cg23668631	CAMKK1	84254	FALSE	0.42(0.29-0.61)	3E−06	0.0001	−1
cg10517312	RNF36	140691	FALSE	2.35(1.64-3.36)	3E−06	0.0001	0
cg16003913	MPG	4350	FALSE	0.46(0.33-0.64)	3E−06	0.0001	0
cg16660041	IQCF1	132141	FALSE	2.29(1.62-3.24)	3E−06	0.0001	0
cg17676574	UROC1	131669	FALSE	2.26(1.6-3.17)	3E−06	0.0001	0
cg17141902	NINJ1	4814	FALSE	2.47(1.69-3.62)	3E−06	0.0001	0
cg21120249	FLJ36268	401563	FALSE	0.44(0.31-0.62)	3E−06	0.0001	−1
cg09210315	SLCO4A1	28231	FALSE	0.45(0.32-0.63)	3E−06	0.0001	0
cg12376406	FAM12B	64184	FALSE	2.25(1.6-3.18)	3E−06	0.0001	0
cg22467071	PECAM1	5175	TRUE	0.42(0.29-0.61)	3E−06	0.0001	0
cg27019278	EMCN	51705	FALSE	0.46(0.33-0.64)	3E−06	0.0001	0
cg26980692	SLC15A3	51296	TRUE	0.45(0.32-0.63)	3E−06	0.0001	0
cg27258399	HTRA4	203100	TRUE	0.43(0.3-0.61)	3E−06	0.0001	0
cg12907644	SAA2	6289	FALSE	0.46(0.33-0.64)	3E−06	0.0001	0
cg05989054	GAMT	2593	TRUE	0.43(0.3-0.62)	3E−06	0.0002	0
cg25659818	CCL4	6351	FALSE	2.31(1.62-3.28)	3E−06	0.0002	0
cg18338021	GZMM	3004	TRUE	2.26(1.61-3.18)	3E−06	0.0002	0
cg26806924	PROCR	10544	FALSE	0.43(0.3-0.62)	3E−06	0.0002	0
cg10896774	C7orf34	135927	FALSE	2.15(1.56-2.97)	3E−06	0.0002	0
cg19067730	PPGB	5476	TRUE	0.44(0.31-0.62)	3E−06	0.0002	0
cg00344372	HGFAC	3083	FALSE	0.43(0.3-0.61)	4E−06	0.0002	0

cg17527798	LTF	4057	TRUE	0.39(0.26-0.58)	4E−06	0.0002	0
cg18854666	SLC11A1	6556	FALSE	0.47(0.34-0.65)	4E−06	0.0002	0
cg25066857	GNLY	10578	FALSE	2.26(1.59-3.2)	4E−06	0.0002	0
cg15407570	STAB1	23166	FALSE	0.43(0.3-0.61)	4E−06	0.0002	0
cg02593766	EPN3	55040	FALSE	0.45(0.32-0.63)	4E−06	0.0002	0
cg13745346	CBFA2T3	863	TRUE	0.43(0.3-0.62)	4E−06	0.0002	−1
cg01968530	C18orf45	85019	TRUE	2.46(1.68-3.61)	4E−06	0.0002	0
cg25061755	STON1	11037	FALSE	2.47(1.68-3.64)	4E−06	0.0002	0
cg02490034	MEST	4232	TRUE	0.43(0.3-0.62)	4E−06	0.0002	0
cg09971811	CST7	8530	FALSE	0.43(0.3-0.61)	4E−06	0.0002	−1
cg04398282	BRDG1	26228	FALSE	2.3(1.61-3.29)	4E−06	0.0002	1
cg21030400	MKNK2	2872	TRUE	2.16(1.56-3.01)	4E−06	0.0002	0
cg13652336	DEPDC2	80243	TRUE	0.45(0.32-0.64)	4E−06	0.0002	0
cg01697865	IL10RA	3587	TRUE	2.4(1.65-3.49)	4E−06	0.0002	1
cg15551881	TRAF1	7185	FALSE	2.79(1.79-4.34)	4E−06	0.0002	0
cg26954174	CARD15	64127	FALSE	0.44(0.3-0.62)	4E−06	0.0002	−1
cg22670733	CHRNA3	1136	TRUE	2.33(1.62-3.36)	5E−06	0.0002	1
cg05790038	TRPV3	162514	FALSE	2.2(1.57-3.09)	5E−06	0.0002	0
cg15746187	FBXO44	93611	FALSE	0.45(0.32-0.64)	5E−06	0.0002	0
cg02227605	ROPN1L	83853	TRUE	0.42(0.29-0.61)	5E−06	0.0002	0
cg26647600	CCR8	1237	FALSE	2.32(1.61-3.33)	5E−06	0.0002	0
cg07258507	IL17E	64806	FALSE	2.29(1.61-3.28)	5E−06	0.0002	0
cg14870461	AER61	285203	TRUE	0.44(0.31-0.63)	5E−06	0.0002	0
cg18350391	IL32	9235	FALSE	2.18(1.56-3.06)	5E−06	0.0002	0
cg12613344	NR1H3	10062	FALSE	0.46(0.33-0.64)	5E−06	0.0002	0
cg24457403	KRT16	3868	FALSE	2.22(1.57-3.14)	5E−06	0.0002	0
cg24183958	GRB10	2887	TRUE	0.43(0.3-0.62)	5E−06	0.0002	0

cg24673765	HSPB6	126393	TRUE	0.44(0.31-0.62)	5E−06	0.0002	0
cg23506842	PTPN7	5778	FALSE	0.42(0.29-0.61)	5E−06	0.0002	0
cg01182697	TMEM59	9528	FALSE	0.46(0.33-0.65)	5E−06	0.0002	0
cg10318258	RIPK3	11035	FALSE	2.12(1.53-2.94)	5E−06	0.0002	0
cg07028533	CNTNAP2	26047	TRUE	0.28(0.16-0.48)	5E−06	0.0002	0
cg23889010	SLPI	6590	FALSE	0.43(0.3-0.62)	5E−06	0.0002	0
cg19539004	LIF	3976	FALSE	0.44(0.31-0.63)	5E−06	0.0002	0
cg08459368	SCGB2A1	4246	FALSE	5.01(2.49-10.1)	5E−06	0.0002	0
cg24262469	TIPARP	25976	TRUE	2.11(1.53-2.91)	5E−06	0.0002	0
cg25623459	TNNI2	7136	FALSE	0.44(0.31-0.63)	6E−06	0.0002	0
cg26928972	CSTA	1475	FALSE	0.45(0.32-0.64)	6E−06	0.0002	0
cg24826867	IRF8	3394	TRUE	5.54(2.64-11.65)	6E−06	0.0002	1
cg04430204	RNF25	64320	FALSE	0.45(0.32-0.64)	6E−06	0.0002	0
cg08519905	CD9	928	TRUE	0.45(0.32-0.64)	6E−06	0.0002	0
cg04108240	TRA2A	29896	TRUE	0.36(0.23-0.57)	6E−06	0.0003	0
cg01618660	TRIM14	9830	TRUE	0.41(0.28-0.61)	6E−06	0.0003	0
cg02927346	RASL10B	91608	TRUE	0.45(0.32-0.64)	6E−06	0.0003	0
cg04180953	DSC1	1823	FALSE	2.21(1.56-3.12)	6E−06	0.0003	0
cg04404982	ITGAL	3683	FALSE	2.14(1.53-2.98)	6E−06	0.0003	0
cg06229674	ARP10	164668	FALSE	2.4(1.64-3.52)	7E−06	0.0003	0
cg22689690	ADCY6	112	TRUE	0.44(0.3-0.63)	7E−06	0.0003	0
cg11721194	SLAMF7	57823	FALSE	2.27(1.59-3.24)	7E−06	0.0003	0
cg18441959	VPREB1	7441	FALSE	2.18(1.55-3.06)	7E−06	0.0003	0
cg03533811	HISPPD2A	9677	TRUE	0.44(0.31-0.63)	7E−06	0.0003	0
cg23959705	TNFRSF9	3604	TRUE	0.47(0.33-0.65)	7E−06	0.0003	0
cg02357714	DOK3	79930	FALSE	0.47(0.34-0.66)	7E−06	0.0003	0
cg16177830	TNFRSF17	608	FALSE	2.23(1.57-3.18)	7E−06	0.0003	0

cg20790540	PTCRA	171558	FALSE	2.24(1.57-3.2)	7E−06	0.0003	0
cg10981541	FAM57A	79850	TRUE	0.44(0.31-0.63)	7E−06	0.0003	0
cg11998307	SCARF1	8578	FALSE	0.46(0.32-0.65)	7E−06	0.0003	0
cg17952262	MFSD7	84179	FALSE	0.46(0.33-0.65)	7E−06	0.0003	0
cg05615150	ARPP-21	10777	FALSE	2.29(1.59-3.31)	7E−06	0.0003	0
cg05859264	MAPK13	5603	FALSE	2.14(1.53-2.99)	7E−06	0.0003	0
cg05508558	CR2	1380	TRUE	2.1(1.52-2.9)	8E−06	0.0003	0
cg19280776	PAG1	55824	TRUE	2.29(1.6-3.26)	8E−06	0.0003	0
cg06810647	CRAMP1L	57585	TRUE	0.47(0.33-0.65)	8E−06	0.0003	0
cg10758292	DEFA1	1667	FALSE	0.46(0.33-0.65)	8E−06	0.0003	0
cg23760945	ELOF1	84337	FALSE	2.26(1.58-3.25)	8E−06	0.0003	1
cg25119415	MNDA	4332	FALSE	2.14(1.53-3.01)	8E−06	0.0003	0
cg25259754	FCRL3	115352	FALSE	2.13(1.52-2.97)	8E−06	0.0003	0
cg00620024	PPP6C	5537	TRUE	2.17(1.54-3.07)	8E−06	0.0003	1
cg21428681	NKX3-1	4824	FALSE	0.47(0.34-0.66)	8E−06	0.0003	0
cg07125166	FBXW12	285231	FALSE	2.23(1.57-3.16)	8E−06	0.0003	0
cg14764661	MRC2	9902	FALSE	0.45(0.32-0.64)	8E−06	0.0003	0
cg10177528	TRAF5	7188	TRUE	2.23(1.56-3.17)	8E−06	0.0003	0
cg16745604	CASP10	843	FALSE	0.46(0.32-0.65)	8E−06	0.0003	0
cg07156669	CPD	1362	TRUE	2.11(1.52-2.94)	8E−06	0.0003	0
cg20395892	IRAK3	11213	TRUE	0.44(0.3-0.63)	9E−06	0.0003	0
cg22628873	GGT6	124975	TRUE	0.45(0.32-0.64)	9E−06	0.0003	0
cg00323915	GIMAP4	55303	FALSE	2.21(1.55-3.14)	9E−06	0.0003	0
cg27345946	TADA3L	10474	FALSE	2.11(1.51-2.95)	9E−06	0.0003	0
cg11397854	IQSEC1	9922	TRUE	0.46(0.32-0.65)	9E−06	0.0003	0
cg05253159	APS	10603	FALSE	0.45(0.32-0.64)	9E−06	0.0003	0
cg01031251	RPS6KA1	6195	TRUE	0.46(0.33-0.65)	9E−06	0.0003	0

cg12554857	PGDS	27306	FALSE	0.47(0.33-0.66)	9E−06	0.0003	0
cg08529852	EGFL7	51162	FALSE	0.49(0.35-0.67)	9E−06	0.0004	0
cg01274660	TRIP6	7205	FALSE	0.46(0.32-0.65)	9E−06	0.0004	0
cg14726637	CR1	1378	TRUE	0.46(0.33-0.65)	9E−06	0.0004	0
cg12832565	CD160	11126	FALSE	2.1(1.51-2.92)	1E−05	0.0004	0
cg05824215	CCR6	1235	FALSE	2.14(1.52-3.01)	1E−05	0.0004	0
cg11630392	GPR171	29909	FALSE	2.17(1.54-3.08)	1E−05	0.0004	0
cg02989940	ERAF	51327	FALSE	2.2(1.55-3.13)	1E−05	0.0004	0
cg12100791	PYCARD	29108	TRUE	0.47(0.34-0.66)	1E−05	0.0004	0
cg26616347	IRF5	3663	TRUE	0.46(0.32-0.65)	1E−05	0.0004	0
cg09276451	SLITL2	114990	TRUE	0.44(0.31-0.64)	1E−05	0.0004	0
cg24715735	HPN	3249	TRUE	0.45(0.32-0.64)	1E−05	0.0004	0
cg04797323	SOCS2	8835	TRUE	0.43(0.29-0.63)	1E−05	0.0004	0
cg00226923	FGD2	221472	FALSE	2.63(1.71-4.06)	1E−05	0.0004	0
cg12091331	PLAT	5327	FALSE	0.48(0.35-0.67)	1E−05	0.0004	−1
cg09076123	NCF2	4688	FALSE	0.48(0.34-0.67)	1E−05	0.0004	−1
cg20289911	C20orf55	83541	FALSE	2.11(1.51-2.95)	1E−05	0.0004	0
cg01500140	LIM2	3982	FALSE	2.11(1.51-2.93)	1E−05	0.0004	0
cg21232015	CHFR	55743	TRUE	2.28(1.58-3.29)	1E−05	0.0004	1
cg04925864	FAM65A	79567	TRUE	0.46(0.32-0.65)	1E−05	0.0004	0
cg04113075	RAB32	10981	TRUE	0.46(0.32-0.65)	1E−05	0.0005	−1
cg04757243	C3orf36	80111	FALSE	2.18(1.53-3.1)	1E−05	0.0005	0
cg19614321	RASSF2	9770	FALSE	0.45(0.31-0.65)	1E−05	0.0005	−1
cg17461214	CHML	1122	FALSE	2.29(1.57-3.32)	1E−05	0.0005	0
cg00615377	RBM9	23543	TRUE	0.46(0.33-0.65)	1E−05	0.0005	0
cg22286764	C3orf35	339883	FALSE	0.5(0.36-0.68)	1E−05	0.0005	0
cg05556202	TM4SF19	116211	FALSE	2.39(1.62-3.54)	1E−05	0.0005	0

cg11394785	LTC4S	4056	FALSE	0.46(0.33-0.66)	1E−05	0.0005	0
cg20366831	APBA3	9546	FALSE	2.08(1.49-2.91)	1E−05	0.0005	0
cg04655481	GPR21	2844	FALSE	0.47(0.34-0.66)	1E−05	0.0005	0
cg16509045	TRPM6	140803	FALSE	0.45(0.31-0.65)	1E−05	0.0005	0
cg16698623	MGMT	4255	TRUE	2.24(1.55-3.23)	1E−05	0.0005	1
cg13553498	CLEC2D	29121	FALSE	2.18(1.53-3.1)	1E−05	0.0005	0
cg22534509	GPR81	27198	TRUE	2.14(1.51-3.03)	1E−05	0.0005	0
cg24674703	CD5	921	FALSE	2.06(1.48-2.87)	1E−05	0.0005	0
cg10516886	CD53	963	FALSE	2.12(1.5-2.98)	1E−05	0.0005	0
cg27491887	KCNQ1	3784	TRUE	0.45(0.32-0.65)	1E−05	0.0005	0
cg08145177	ABHD8	79575	TRUE	0.47(0.33-0.66)	1E−05	0.0005	0
cg25298754	ZBED2	79413	FALSE	2.12(1.5-2.98)	1E−05	0.0005	0
cg27187881	NAGA	4668	TRUE	0.45(0.31-0.64)	1E−05	0.0005	−1
cg17166812	NDUFS2	4720	FALSE	0.49(0.35-0.68)	2E−05	0.0005	0
cg06521852	HRIHFB2122	11078	TRUE	0.48(0.34-0.67)	2E−05	0.0005	0
cg04600618	C6orf206	221421	TRUE	0.47(0.33-0.66)	2E−05	0.0005	0
cg22438810	LCN2	3934	FALSE	0.49(0.36-0.68)	2E−05	0.0005	0
cg05246522	KSR1	8844	FALSE	2.16(1.52-3.06)	2E−05	0.0005	0
cg15691199	CEBPE	1053	FALSE	0.48(0.34-0.67)	2E−05	0.0005	0
cg10368842	C10orf81	79949	FALSE	2.05(1.48-2.85)	2E−05	0.0005	0
cg07057831	EPIM	2054	TRUE	2.04(1.48-2.81)	2E−05	0.0005	0
cg17271365	MARCH1	55016	FALSE	0.5(0.36-0.68)	2E−05	0.0006	0
cg00031162	TNFSF12-TNFSF13	407977	FALSE	2.17(1.52-3.11)	2E−05	0.0006	0
cg26163537	GRB10	2887	TRUE	0.47(0.33-0.66)	2E−05	0.0006	0
cg01129847	C19orf35	374872	FALSE	0.47(0.34-0.67)	2E−05	0.0006	−1
cg17386185	GLYCTK	132158	TRUE	0.47(0.34-0.67)	2E−05	0.0006	−1
cg12417466	ARPP-21	10777	FALSE	2.17(1.52-3.08)	2E−05	0.0006	0

cg23579062	DNAI1	27019	TRUE	0.47(0.34-0.67)	2E−05	0.0006	0
cg24921089	AMPD3	272	TRUE	0.49(0.35-0.68)	2E−05	0.0006	0
cg16989646	SLC25A15	10166	TRUE	0.48(0.35-0.68)	2E−05	0.0006	0
cg13504059	CCR7	1236	FALSE	2.05(1.48-2.85)	2E−05	0.0006	0
cg12014417	GPR109A	338442	TRUE	0.46(0.32-0.66)	2E−05	0.0006	−1
cg18538812	GIF	2694	FALSE	2.17(1.53-3.1)	2E−05	0.0006	0
cg03714916	CDKN1A	1026	FALSE	0.49(0.35-0.68)	2E−05	0.0006	0
cg16378421	ABCC5	10057	TRUE	0.47(0.33-0.66)	2E−05	0.0006	0
cg04286933	APOBEC3G	60489	FALSE	0.48(0.34-0.67)	2E−05	0.0006	−1
cg10521852	EDG4	9170	TRUE	0.47(0.33-0.67)	2E−05	0.0006	0
cg01965939	SH3TC2	79628	FALSE	0.48(0.35-0.68)	2E−05	0.0006	0
cg13316191	CDCA7L	55536	TRUE	0.44(0.3-0.64)	2E−05	0.0006	0
cg04491089	MBTPS1	8720	TRUE	2.02(1.47-2.78)	2E−05	0.0006	0
cg14898779	STK31	56164	TRUE	0.49(0.36-0.68)	2E−05	0.0006	0
cg12586262	ZNF160	90338	TRUE	2.01(1.46-2.78)	2E−05	0.0006	0
cg12391783	NSMCE1	197370	FALSE	0.46(0.32-0.65)	2E−05	0.0006	0
cg00739120	NIFIE14	10430	TRUE	0.49(0.35-0.68)	2E−05	0.0006	0
cg15662251	PAQR7	164091	FALSE	0.49(0.35-0.68)	2E−05	0.0006	0
cg19464252	FBS1	64319	FALSE	2.09(1.48-2.94)	2E−05	0.0006	0
cg11164400	PPP1R9A	55607	TRUE	0.45(0.31-0.65)	2E−05	0.0006	0
cg06736444	SRRM2	23524	TRUE	2.1(1.49-2.95)	2E−05	0.0006	0
cg21283680	SH3BP5	9467	FALSE	0.49(0.35-0.68)	2E−05	0.0006	0
cg01172735	SESN2	83667	TRUE	0.46(0.32-0.66)	2E−05	0.0006	0
cg08965143	TP53I3	9540	TRUE	0.46(0.32-0.66)	2E−05	0.0006	0
cg20993403	EPB41L1	2036	FALSE	2.14(1.5-3.04)	2E−05	0.0007	0
cg08241785	F2RL2	2151	FALSE	0.46(0.33-0.66)	2E−05	0.0007	−1
cg24456340	GNGT2	2793	TRUE	2.07(1.48-2.9)	2E−05	0.0007	0

cg10494770	IGLL1	3543	FALSE	0.49(0.35-0.68)	2E−05	0.0007	0
cg23713742	SPAG4	6676	FALSE	0.49(0.36-0.69)	2E−05	0.0007	0
cg03763616	SPIB	6689	FALSE	0.49(0.35-0.68)	2E−05	0.0007	0
cg00168785	WDSUB1	151525	TRUE	0.49(0.35-0.68)	2E−05	0.0007	0
cg14292823	TIGD5	84948	TRUE	0.47(0.33-0.67)	2E−05	0.0007	0
cg04490714	SLC6A2	6530	TRUE	0.36(0.23-0.58)	2E−05	0.0007	0
cg08510456	BSN	8927	TRUE	0.45(0.31-0.65)	2E−05	0.0007	0
cg23606023	CBFA2T3	863	FALSE	0.49(0.35-0.68)	2E−05	0.0007	0
cg11105610	LGALS3BP	3959	FALSE	2.17(1.51-3.12)	2E−05	0.0007	0
cg18413900	CYP27B1	1594	FALSE	0.48(0.34-0.67)	2E−05	0.0007	0
cg26989531	CCND2	894	TRUE	2.02(1.46-2.79)	2E−05	0.0007	0
cg03270167	RAMP1	10267	TRUE	0.49(0.35-0.68)	2E−05	0.0007	0
cg22322184	RETN	56729	FALSE	0.48(0.34-0.67)	2E−05	0.0007	0
cg08815403	HSD17B13	345275	FALSE	2.11(1.49-2.98)	2E−05	0.0007	0
cg24070847	HIST2H2BE	8349	TRUE	2(1.45-2.75)	2E−05	0.0007	0
cg11738543	SOCS2	8835	TRUE	0.44(0.3-0.65)	2E−05	0.0007	0
cg04536922	FAM13A1	10144	FALSE	0.46(0.32-0.66)	2E−05	0.0007	0
cg02310296	MMP9	4318	FALSE	0.48(0.34-0.67)	2E−05	0.0007	0
cg09554443	CD3Z	919	TRUE	1.97(1.43-2.71)	2E−05	0.0007	1
cg21633698	THY1	7070	FALSE	2(1.45-2.77)	2E−05	0.0007	0
cg07658590	SLC19A1	6573	FALSE	0.47(0.33-0.67)	2E−05	0.0007	−1
cg05615487	ACR	49	FALSE	2.03(1.45-2.83)	2E−05	0.0007	0
cg09106999	CDK2	1017	FALSE	0.49(0.35-0.68)	2E−05	0.0007	0
cg26392924	SLC23A1	9963	FALSE	0.49(0.35-0.68)	2E−05	0.0007	0
cg11584690	ZNF574	64763	FALSE	0.48(0.34-0.67)	2E−05	0.0007	0
cg10465696	ROBO4	54538	TRUE	0.48(0.34-0.68)	2E−05	0.0008	0
cg12243271	CFI	3426	FALSE	0.45(0.31-0.66)	3E−05	0.0008	0

cg02786019	TRPV6	55503	FALSE	2.1(1.48-2.97)	3E−05	0.0008	0
cg26233914	ITGAX	3687	FALSE	0.45(0.31-0.66)	3E−05	0.0008	−1
cg09427311	ANGPTL2	23452	FALSE	0.48(0.34-0.68)	3E−05	0.0008	−1
cg16854606	DAND5	199699	FALSE	2.01(1.45-2.8)	3E−05	0.0008	0
cg08290628	CORO2B	10391	FALSE	0.51(0.37-0.7)	3E−05	0.0008	0
cg19787037	SPAG11	10407	FALSE	2.06(1.47-2.9)	3E−05	0.0008	0
cg11965370	HNT	50863	TRUE	0.5(0.36-0.69)	3E−05	0.0008	0
cg12491710	LIM2	3982	TRUE	2.21(1.54-3.17)	3E−05	0.0008	0
cg07360692	FLJ20032	54790	TRUE	0.5(0.36-0.69)	3E−05	0.0008	0
cg23854009	ZNF530	348327	TRUE	0.46(0.32-0.66)	3E−05	0.0008	0
cg20530056	IKBKE	9641	FALSE	2.12(1.49-3.03)	3E−05	0.0008	0
cg19168338	CORO7	79585	TRUE	0.46(0.32-0.66)	3E−05	0.0008	−1
cg25112191	RORC	6097	FALSE	0.51(0.37-0.7)	3E−05	0.0008	0
cg07426848	S100A3	6274	FALSE	2.14(1.49-3.06)	3E−05	0.0008	0
cg06029655	SBP1	90198	FALSE	2.3(1.56-3.38)	3E−05	0.0008	0
cg07914866	IRAK3	11213	TRUE	0.5(0.36-0.69)	3E−05	0.0009	0
cg10872212	KLHDC8B	200942	TRUE	0.48(0.34-0.68)	3E−05	0.0009	0
cg17682828	FXYD7	53822	TRUE	2.22(1.53-3.22)	3E−05	0.0009	0
cg00615241	PRTN3	5657	FALSE	0.52(0.38-0.71)	3E−05	0.0009	0
cg11668844	MCF2L	23263	TRUE	0.49(0.35-0.69)	3E−05	0.0009	0
cg14861570	MMD	23531	FALSE	2.08(1.48-2.92)	3E−05	0.0009	0
cg00013618		3535	FALSE	2.07(1.46-2.93)	3E−05	0.0009	0
cg14150666	IL8RB	3579	FALSE	0.52(0.38-0.7)	3E−05	0.0009	0
cg22485810	CENTA2	55803	TRUE	0.48(0.34-0.68)	3E−05	0.0009	0
cg11484872	TNF	7124	FALSE	1.99(1.45-2.74)	3E−05	0.0009	0
cg17468997	NCF1	4687	FALSE	0.49(0.35-0.69)	3E−05	0.0009	0
cg14761227	CPA1	1357	FALSE	2.1(1.47-2.98)	3E−05	0.0009	0

cg05190718	CASQ2	845	FALSE	2.05(1.46-2.87)	3E−05	0.0009	0
cg13735974	NFYC	4802	TRUE	2.14(1.49-3.06)	3E−05	0.0009	0
cg03649060	ELOF1	84337	FALSE	2.29(1.54-3.39)	3E−05	0.0009	0
cg13299148	COL16A1	1307	FALSE	0.47(0.33-0.68)	3E−05	0.0009	0
cg27223047	FBN2	2201	TRUE	0.49(0.35-0.69)	3E−05	0.0009	0
cg14162076	CLEC4D	338339	TRUE	2.67(1.67-4.27)	3E−05	0.0009	0
cg11832722	DSC3	1825	TRUE	0.47(0.33-0.66)	3E−05	0.001	0
cg22289810	NF1	4763	TRUE	2.03(1.46-2.83)	4E−05	0.001	0
cg24354652	PTAFR	5724	FALSE	0.5(0.36-0.7)	4E−05	0.001	0
cg13531460	GPR55	9290	FALSE	2.71(1.68-4.38)	4E−05	0.001	0
cg06151964	HPN	3249	TRUE	0.5(0.36-0.69)	4E−05	0.001	0
cg25725843	ST6GAL2	84620	TRUE	0.43(0.29-0.64)	4E−05	0.001	0
cg02100848	C3orf32	51066	FALSE	2.17(1.5-3.15)	4E−05	0.001	0
cg21033855	ZNF690	146050	TRUE	0.5(0.36-0.7)	4E−05	0.001	0
cg07705835	IL17RC	84818	FALSE	0.48(0.34-0.68)	4E−05	0.001	−1
cg02569613	C10orf72	196740	TRUE	0.48(0.34-0.68)	4E−05	0.001	0
cg00513220	MOSC2	54996	TRUE	0.5(0.36-0.7)	4E−05	0.001	0
cg11277126	TRPC4AP	26133	TRUE	2.09(1.47-2.95)	4E−05	0.001	0
cg10094443	UGDH	7358	TRUE	0.49(0.35-0.69)	4E−05	0.001	0
cg11846968	PLUNC	51297	FALSE	0.52(0.38-0.71)	4E−05	0.001	0
cg15052335	LPIN2	9663	TRUE	2.07(1.46-2.92)	4E−05	0.001	0
cg09076077	FLJ33860	284756	TRUE	0.48(0.34-0.68)	4E−05	0.001	0
cg17738194	GK2	2712	TRUE	2(1.44-2.77)	4E−05	0.001	0
cg00491404	EPS8L3	79574	FALSE	2.1(1.47-3)	4E−05	0.001	0
cg20083676	EDG3	1903	TRUE	0.51(0.37-0.7)	4E−05	0.001	0
cg11835355	APOL3	80833	FALSE	0.52(0.38-0.71)	4E−05	0.001	0
cg22876908	DGKA	1606	FALSE	2(1.43-2.8)	4E−05	0.001	0

cg03600318	SFTPD	6441	FALSE	0.51(0.37-0.7)	4E−05	0.001	0
cg20018806	TCN1	6947	FALSE	0.51(0.37-0.7)	4E−05	0.001	0
cg22285621	SSH3	54961	TRUE	0.47(0.32-0.68)	4E−05	0.001	0
cg11714502	AK1	203	TRUE	0.51(0.37-0.71)	4E−05	0.001	−1
cg20790056	MGC23244	126259	FALSE	2.2(1.5-3.21)	4E−05	0.001	1
cg11271605	STEAP4	79689	TRUE	0.49(0.35-0.69)	4E−05	0.001	0
cg26267561	OXT	5020	TRUE	0.5(0.36-0.7)	4E−05	0.001	0
cg20535085	SLAMF1	6504	FALSE	2.03(1.44-2.86)	4E−05	0.001	1
cg22775000	TMEFF1	8577	TRUE	0.51(0.37-0.71)	4E−05	0.001	0
cg16046376	PC	5091	FALSE	0.51(0.37-0.71)	4E−05	0.001	0
cg23577242	MPPED2	744	FALSE	0.45(0.3-0.66)	5E−05	0.001	0
cg20287234	GPR55	9290	TRUE	0.49(0.35-0.69)	5E−05	0.001	0
cg15820955	HDAC7A	51564	FALSE	2.09(1.46-2.98)	5E−05	0.001	0
cg18940763	XBP1	7494	TRUE	0.48(0.33-0.68)	5E−05	0.001	0
cg21474838	KLRD1	3824	FALSE	2.12(1.47-3.04)	5E−05	0.001	0
cg24352688	OFD1	8481	FALSE	2.01(1.43-2.82)	5E−05	0.001	0
cg13688966	TM4SF4	7104	FALSE	2.02(1.44-2.83)	5E−05	0.001	0
cg04121983	CASKIN2	57513	TRUE	0.51(0.37-0.71)	5E−05	0.001	0
cg20191453	AMT	275	TRUE	0.48(0.34-0.69)	5E−05	0.001	0
cg06256735	MFAP5	8076	FALSE	2.36(1.56-3.56)	5E−05	0.001	0
cg22858861	CCDC5	115106	TRUE	2.12(1.48-3.05)	5E−05	0.001	0
cg20904010	SYN3	8224	FALSE	2.31(1.55-3.45)	5E−05	0.001	0
cg26304237	DNAJC6	9829	TRUE	0.48(0.33-0.68)	5E−05	0.001	0
cg12113819	THRAP2	23389	TRUE	0.5(0.36-0.7)	5E−05	0.001	−1
cg06311778	TENC1	23371	TRUE	0.51(0.36-0.71)	5E−05	0.001	0
cg23307338	MS4A12	54860	FALSE	2.07(1.46-2.95)	5E−05	0.001	0
cg13500819	PACAP	51237	FALSE	2.09(1.46-3)	5E−05	0.001	0

cg15013019	LYL1	4066	TRUE	0.5(0.36-0.7)	5E−05	0.001	0
cg00622552	ODF3L1	161753	FALSE	0.5(0.35-0.7)	5E−05	0.001	−1
cg10305797	UNQ467	388533	FALSE	2.17(1.49-3.15)	5E−05	0.001	0
cg23181133	CEACAM3	1084	FALSE	0.52(0.38-0.72)	5E−05	0.001	0
cg11473104	NUDT15	55270	TRUE	2.51(1.6-3.93)	5E−05	0.001	0
cg22321558	FLJ90024	129303	TRUE	0.49(0.34-0.69)	5E−05	0.001	0
cg15796819	ALDH3A1	218	FALSE	1.99(1.43-2.78)	5E−05	0.001	0
cg21448423	ACOT11	26027	FALSE	0.52(0.38-0.72)	5E−05	0.001	−1
cg03310469	SIX2	10736	TRUE	0.46(0.32-0.67)	5E−05	0.001	0
cg09500672	MGC52057	130574	TRUE	0.5(0.36-0.69)	5E−05	0.001	0
cg16270990	C11orf44	283171	FALSE	1.98(1.42-2.77)	5E−05	0.001	0
cg00278366	RAD9B	144715	TRUE	0.52(0.37-0.71)	5E−05	0.001	0
cg14102807	CD19	930	FALSE	2.17(1.49-3.15)	5E−05	0.001	0
cg20781967	NINJ2	4815	FALSE	1.92(1.4-2.65)	5E−05	0.001	0
cg21207436	C14orf115	55237	FALSE	1.97(1.41-2.74)	5E−05	0.001	0
cg14700707	NOTCH4	4855	FALSE	0.5(0.36-0.7)	5E−05	0.001	−1
cg12962778	ZNF385	25946	FALSE	0.53(0.39-0.72)	5E−05	0.001	0
cg26111757	C20orf185	359710	FALSE	1.96(1.41-2.72)	5E−05	0.001	0
cg11360718	TM9SF4	9777	FALSE	1.94(1.4-2.68)	5E−05	0.001	0
cg07509155	IL28RA	163702	TRUE	0.49(0.34-0.7)	5E−05	0.001	0
cg17753124	IER2	9592	FALSE	0.51(0.37-0.71)	6E−05	0.001	0
cg00331237	CSRP2	1466	TRUE	0.51(0.37-0.71)	6E−05	0.001	0
cg15582789	SLC35D2	11046	TRUE	0.52(0.38-0.72)	6E−05	0.001	0
cg21372914	CLEC4M	10332	FALSE	1.97(1.41-2.75)	6E−05	0.001	0
cg24821554	GUCY1B2	2974	FALSE	0.51(0.36-0.71)	6E−05	0.001	−1
cg01143454	C20orf141	128653	FALSE	0.52(0.37-0.72)	6E−05	0.001	0
cg04198824	USPL1	10208	TRUE	1.97(1.41-2.74)	6E−05	0.001	0

cg12491659	FLJ33641	202309	FALSE	0.52(0.37-0.71)	6E−05	0.001	0
cg00619207	DENND2D	79961	FALSE	1.93(1.4-2.68)	6E−05	0.001	0
cg00823148	CRYGB	1419	FALSE	1.99(1.42-2.79)	6E−05	0.001	0
cg13349425	C5orf20	140947	FALSE	2.11(1.47-3.03)	6E−05	0.001	0
cg26379475	SH2D1B	117157	FALSE	2.13(1.48-3.07)	6E−05	0.002	0
cg20956314	XYLB	9942	FALSE	0.43(0.28-0.65)	6E−05	0.002	0
cg13350783	KRT13	3860	FALSE	2.13(1.48-3.08)	6E−05	0.002	0
cg00554250	NR1H3	10062	FALSE	0.5(0.36-0.71)	6E−05	0.002	0
cg01120308	PICALM	8301	FALSE	0.5(0.36-0.71)	6E−05	0.002	0
cg25402049	PRDM2	7799	FALSE	0.53(0.39-0.72)	6E−05	0.002	0
cg08840010	TNFRSF9	3604	FALSE	0.5(0.36-0.71)	6E−05	0.002	0
cg17995823	CTNND1	1500	FALSE	0.48(0.33-0.69)	6E−05	0.002	0
cg22630748	INHBE	83729	FALSE	0.52(0.37-0.72)	6E−05	0.002	0
cg13760253	DNAJC5B	85479	FALSE	0.52(0.38-0.72)	6E−05	0.002	0
cg25216696	GATA4	2626	TRUE	0.47(0.33-0.68)	6E−05	0.002	0
cg08743392	GSS	2937	TRUE	0.47(0.32-0.69)	6E−05	0.002	−1
cg01560871	C10orf27	219793	FALSE	0.5(0.35-0.7)	7E−05	0.002	−1
cg01449591	BIRC1	4671	FALSE	2.01(1.42-2.85)	7E−05	0.002	0
cg23499956	S100A16	140576	FALSE	2.04(1.44-2.89)	7E−05	0.002	0
cg27347104	VWF	7450	FALSE	0.51(0.37-0.71)	7E−05	0.002	−1
cg16335762	CMTM3	123920	TRUE	2.03(1.43-2.89)	7E−05	0.002	0
cg00666746	SYDE1	85360	TRUE	0.52(0.37-0.72)	7E−05	0.002	0
cg20483374	C1QTNF5	114902	TRUE	0.53(0.39-0.72)	7E−05	0.002	0
cg17463527	SGK2	10110	FALSE	1.97(1.41-2.77)	7E−05	0.002	0
cg09350141	PPM1F	9647	TRUE	0.51(0.37-0.71)	7E−05	0.002	0
cg15779716	CDCP1	64866	TRUE	0.53(0.38-0.73)	7E−05	0.002	0
cg09791681	TMCO3	55002	TRUE	1.92(1.39-2.66)	7E−05	0.002	0

cg00850538	CRIM1	51232	TRUE	0.5(0.35-0.71)	7E−05	0.002	0
cg12412075	LIN7B	64130	TRUE	0.51(0.37-0.71)	7E−05	0.002	0
cg01172972	ZFYVE9	9372	TRUE	0.5(0.36-0.71)	7E−05	0.002	−1
cg00679556	TRIM31	11074	FALSE	2.12(1.46-3.06)	7E−05	0.002	0
cg20856834	OR12D3	81797	FALSE	1.93(1.39-2.69)	7E−05	0.002	0
cg21892409	C11orf49	79096	TRUE	1.95(1.4-2.72)	7E−05	0.002	0
cg19008649	IGFBP5	3488	TRUE	0.49(0.34-0.7)	7E−05	0.002	0
cg10315334	CCL5	6352	FALSE	1.91(1.38-2.65)	7E−05	0.002	0
cg13243219	MDS1	4197	TRUE	1.91(1.38-2.63)	7E−05	0.002	0
cg07638935	KAZALD1	81621	TRUE	0.43(0.28-0.65)	7E−05	0.002	0
cg06392589	FNDC6	152028	FALSE	0.52(0.38-0.72)	7E−05	0.002	0
cg01550148	H2AFY	9555	TRUE	0.51(0.36-0.71)	7E−05	0.002	0
cg18152830	TNFRSF13B	23495	FALSE	2.24(1.5-3.34)	8E−05	0.002	0
cg17329164	PPT2	9374	TRUE	0.53(0.39-0.73)	8E−05	0.002	0
cg22705225	PDZK3	23037	TRUE	0.54(0.39-0.73)	8E−05	0.002	0
cg13030582	MFAP4	4239	FALSE	0.51(0.37-0.72)	8E−05	0.002	0
cg10997248	FXYD2	486	FALSE	1.94(1.39-2.69)	8E−05	0.002	0
cg14951292	HMOX2	3163	TRUE	1.9(1.38-2.61)	8E−05	0.002	0
cg02794695	SLA	6503	FALSE	2.06(1.44-2.96)	8E−05	0.002	0
cg19005368	PRRG4	79056	TRUE	0.51(0.36-0.71)	8E−05	0.002	0
cg05751148	PTPRCAP	5790	FALSE	1.95(1.39-2.72)	8E−05	0.002	0
cg24862483	CD300LG	146894	TRUE	0.52(0.37-0.72)	8E−05	0.002	0
cg26227465	IFNG	3458	FALSE	1.96(1.4-2.75)	8E−05	0.002	0
cg20840847	APBA2	321	FALSE	2.11(1.46-3.06)	8E−05	0.002	0
cg03821311	HIST1H1B	3009	FALSE	0.51(0.36-0.71)	9E−05	0.002	0
cg18109798	SLCO1C1	53919	FALSE	1.9(1.38-2.61)	9E−05	0.002	0
cg14435807	LOXL1	4016	TRUE	0.49(0.34-0.7)	9E−05	0.002	−1

cg07409200	FLJ40919	144809	FALSE	0.5(0.35-0.71)	9E−05	0.002	0
cg06426831	SLC35A3	23443	TRUE	0.53(0.39-0.73)	9E−05	0.002	−1
cg24881834	ME1	4199	TRUE	0.44(0.29-0.66)	9E−05	0.002	0
cg02605634	WFDC10A	140832	FALSE	0.48(0.33-0.7)	9E−05	0.002	0
cg09990086	TACC1	6867	TRUE	0.52(0.38-0.72)	9E−05	0.002	0
cg01573562	RPH3A	22895	TRUE	0.49(0.34-0.7)	9E−05	0.002	0
cg10001720	LAPTM5	7805	FALSE	0.53(0.39-0.73)	9E−05	0.002	0
cg10737521	KIAA0676	23061	TRUE	0.53(0.38-0.73)	9E−05	0.002	0
cg14785479	SCARF2	91179	TRUE	0.46(0.31-0.68)	9E−05	0.002	0
cg23412777	PYGO1	26108	TRUE	0.52(0.38-0.72)	9E−05	0.002	0
cg15952487	CD1B	910	FALSE	1.84(1.35-2.51)	9E−05	0.002	0
cg21602520	BCL2	596	TRUE	1.94(1.39-2.7)	0.0001	0.002	0
cg04872689	PLEK	5341	FALSE	0.52(0.37-0.72)	0.0001	0.002	0
cg16068833	CD52	1043	FALSE	1.87(1.36-2.58)	0.0001	0.002	0
cg06183267	AFF3	3899	FALSE	1.98(1.4-2.8)	0.0001	0.002	0
cg13180098	RHO	6010	FALSE	1.9(1.37-2.63)	0.0001	0.002	0
cg07039362	CES7	221223	FALSE	1.99(1.4-2.83)	0.0001	0.002	0
cg19524009	NEK3	4752	FALSE	0.54(0.39-0.74)	0.0001	0.002	0
cg01036779	SH2D3A	10045	TRUE	2.38(1.56-3.63)	0.0001	0.002	0
cg26482939	GNA15	2769	TRUE	0.5(0.35-0.71)	0.0001	0.002	0
cg24320643	ADAM17	6868	TRUE	2.19(1.47-3.26)	0.0001	0.002	0
cg19210770	ACCN4	55515	TRUE	0.48(0.34-0.7)	0.0001	0.002	0
cg17207590	APH1B	83464	TRUE	0.53(0.39-0.73)	0.0001	0.002	0
cg10453365	RHCG	51458	TRUE	0.52(0.37-0.72)	0.0001	0.002	0
cg24910675	ENG	2022	TRUE	0.49(0.34-0.7)	0.0001	0.002	0
cg11476254	LOC197322	197322	TRUE	0.52(0.37-0.73)	0.0001	0.002	0
cg18493182	LYPD2	137797	FALSE	1.94(1.39-2.7)	0.0001	0.002	0

cg04301614	FLJ11017	55286	FALSE	0.4(0.25-0.63)	0.0001	0.002	0
cg07314414	SAP130	79595	TRUE	2.32(1.51-3.56)	0.0001	0.002	0
cg05822532	ELN	2006	TRUE	0.49(0.34-0.7)	0.0001	0.002	−1
cg14182690	RUNX3	864	TRUE	2.05(1.42-2.97)	0.0001	0.002	0
cg24058132	GALC	2581	TRUE	1.85(1.36-2.51)	0.0001	0.002	0
cg15322932	ALDH3B1	221	FALSE	0.5(0.35-0.71)	0.0001	0.002	−1
cg14188111	S100A13	6284	TRUE	1.97(1.39-2.78)	0.0001	0.002	0
cg07443748	CESK1	150160	FALSE	1.96(1.39-2.76)	0.0001	0.002	0
cg09300114	SLC16A5	9121	TRUE	0.51(0.36-0.72)	0.0001	0.002	0
cg15937958	UNQ473	284340	FALSE	0.54(0.39-0.74)	0.0001	0.002	0
cg02512860	CLDN15	24146	FALSE	0.55(0.4-0.74)	0.0001	0.002	0
cg10688991	DLX3	1747	TRUE	0.52(0.37-0.72)	0.0001	0.002	0
cg10787197	C6orf105	84830	FALSE	0.54(0.39-0.74)	0.0001	0.002	0
cg12949760	KCNQ1	3784	TRUE	0.54(0.4-0.74)	0.0001	0.002	−1
cg04901273	TBC1D3	84218	FALSE	2.05(1.42-2.95)	0.0001	0.002	0
cg07693270	RPL39L	116832	TRUE	1.82(1.34-2.48)	0.0001	0.002	0
cg15361231	GLRX2	51022	FALSE	0.51(0.36-0.72)	0.0001	0.003	0
cg22932215	COPS7B	64708	TRUE	1.97(1.39-2.79)	0.0001	0.003	0
cg23338993	UGT1A6	54578	FALSE	1.86(1.35-2.55)	0.0001	0.003	0
cg13060154	DAB2IP	153090	TRUE	0.53(0.38-0.73)	0.0001	0.003	0
cg07970007	GBP1	2633	FALSE	1.86(1.35-2.56)	0.0001	0.003	0
cg14366598	IL17E	64806	FALSE	1.91(1.37-2.66)	0.0001	0.003	0
cg05421688	C1orf76	148753	TRUE	0.51(0.36-0.72)	0.0001	0.003	0
cg16608348	PTPN14	5784	TRUE	0.51(0.36-0.72)	0.0001	0.003	0
cg03860768	BLK	640	FALSE	1.97(1.39-2.8)	0.0001	0.003	0
cg05666713	DKFZp434B1231	91156	FALSE	1.99(1.4-2.82)	0.0001	0.003	0
cg24678320	FLJ38451	126375	FALSE	0.5(0.36-0.71)	0.0001	0.003	0

cg10182321	STK32B	55351	TRUE	0.52(0.37-0.72)	0.0001	0.003	0
cg19635695	PDE6C	5146	FALSE	1.94(1.38-2.73)	0.0001	0.003	0
cg15005385	CCL3L1	6349	FALSE	1.93(1.38-2.7)	0.0001	0.003	0
cg09018862	SBP1	90198	FALSE	1.92(1.37-2.69)	0.0001	0.003	0
cg09606564	MFAP4	4239	FALSE	0.51(0.36-0.73)	0.0001	0.003	0
cg20789824	OLFML2A	169611	FALSE	0.55(0.4-0.75)	0.0001	0.003	0
cg06105085	C10orf104	119504	TRUE	1.83(1.34-2.5)	0.0001	0.003	0
cg01851399	PKP2	5318	TRUE	0.49(0.34-0.71)	0.0001	0.003	0
cg05715649	SYT2	127833	TRUE	0.53(0.39-0.74)	0.0001	0.003	0
cg13078388	CIRBP	1153	TRUE	1.97(1.42-2.75)	0.0001	0.003	0
cg14547335	ATP2B2	491	TRUE	1.91(1.37-2.67)	0.0001	0.003	0
cg02854288	FHIT	2272	TRUE	0.51(0.36-0.71)	0.0001	0.003	0
cg13625113	ZNF482	10773	TRUE	1.91(1.37-2.68)	0.0001	0.003	0
cg05718253	LOC55908	55908	FALSE	0.52(0.38-0.73)	0.0001	0.003	0
cg07651914	CLDN15	24146	FALSE	0.55(0.4-0.74)	0.0001	0.003	0
cg04123507	KRTHB6	3892	TRUE	0.42(0.27-0.65)	0.0001	0.003	0
cg22165175	KCNA2	3737	TRUE	0.54(0.39-0.74)	0.0001	0.003	0
cg05102817	RBM18	92400	FALSE	0.51(0.36-0.72)	0.0001	0.003	0
cg07220939	SLC22A12	116085	TRUE	0.54(0.39-0.74)	0.0001	0.003	0
cg09440340	C1orf161	126868	FALSE	2.25(1.48-3.43)	0.0001	0.003	0
cg13530946	IARS2	55699	FALSE	0.46(0.31-0.69)	0.0001	0.003	0
cg27235662	CLDN16	10686	FALSE	1.83(1.34-2.51)	0.0001	0.003	0
cg27557796	LY6G6C	80740	FALSE	2.04(1.43-2.89)	0.0001	0.003	0
cg07732037	MPHOSPH9	10198	FALSE	1.86(1.35-2.57)	0.0001	0.003	0
cg03001305	STAT5A	6776	FALSE	0.52(0.37-0.73)	0.0001	0.003	−1
cg25282780	AK3	50808	TRUE	1.95(1.38-2.75)	0.0001	0.003	0
cg24408313	BCL2	596	TRUE	0.54(0.39-0.74)	0.0001	0.003	0

cg16280667	BLR1	643	TRUE	2.02(1.4-2.91)	0.0001	0.003	0
cg24576425	GALNT5	11227	FALSE	0.53(0.38-0.74)	0.0001	0.003	0
cg13382714	AP4S1	11154	TRUE	1.83(1.34-2.5)	0.0001	0.003	0
cg12876594	NPR2	4882	TRUE	0.53(0.38-0.74)	0.0001	0.003	0
cg10599444	MMP14	4323	TRUE	0.52(0.37-0.73)	0.0001	0.003	0
cg25218351	TRIM54	57159	FALSE	1.87(1.35-2.58)	0.0001	0.003	0
cg26842024	KLF2	10365	TRUE	1.86(1.35-2.57)	0.0001	0.003	1
cg05973262	NOTCH4	4855	FALSE	0.53(0.38-0.74)	0.0001	0.003	0
cg25384595	LILRA1	11024	FALSE	0.54(0.39-0.74)	0.0001	0.003	0
cg02601403	TBC1D3C	414060	FALSE	1.95(1.38-2.75)	0.0001	0.003	0
cg01669948	KCNK16	83795	FALSE	1.91(1.37-2.68)	0.0001	0.003	0
cg05130485	CASP8	841	FALSE	2.01(1.4-2.89)	0.0001	0.003	0
cg02196805	CSF2	1437	FALSE	1.84(1.34-2.53)	0.0001	0.003	0
cg17918239	ZNF281	23528	FALSE	2.01(1.4-2.89)	0.0001	0.003	0
cg25242557	PAX6	5080	TRUE	0.51(0.36-0.72)	0.0001	0.003	0
cg04008901	CACNA2D1	781	TRUE	0.46(0.31-0.68)	0.0001	0.003	0
cg09195271	RNF186	54546	FALSE	2.03(1.41-2.94)	0.0001	0.003	1
cg10905918	RPS24	6229	TRUE	1.91(1.37-2.68)	0.0001	0.003	0
cg17237063	RBMS3	27303	FALSE	1.92(1.37-2.69)	0.0001	0.003	0
cg13828871	MYST1	84148	TRUE	2.04(1.42-2.94)	0.0002	0.003	0
cg24250393	PRKCB1	5579	TRUE	0.55(0.4-0.75)	0.0002	0.003	0
cg02449608	C19orf18	147685	FALSE	1.9(1.36-2.66)	0.0002	0.003	0
cg09531892	LRP12	29967	TRUE	0.47(0.32-0.69)	0.0002	0.003	0
cg25866075	NALP12	91662	FALSE	0.5(0.35-0.72)	0.0002	0.003	0
cg27285720	GBP4	115361	FALSE	2.33(1.5-3.64)	0.0002	0.003	0
cg12578166	KCNQ1	3784	TRUE	1.84(1.34-2.52)	0.0002	0.003	0
cg12788313	MST1	4485	FALSE	0.55(0.41-0.75)	0.0002	0.003	0

cg18818531	FOSL1	8061	TRUE	1.86(1.35-2.56)	0.0002	0.003	0
cg26335299	C16orf48	84080	TRUE	0.53(0.38-0.74)	0.0002	0.003	0
cg15923513	CD302	9936	TRUE	0.53(0.39-0.74)	0.0002	0.003	0
cg18530324	KIAA0427	9811	TRUE	0.54(0.39-0.75)	0.0002	0.003	0
cg18669381	ARHGEF19	128272	FALSE	1.87(1.35-2.59)	0.0002	0.003	0
cg04273431	PRR3	80742	TRUE	0.56(0.41-0.76)	0.0002	0.003	0
cg01309671	RP3-473B4.1	159091	TRUE	1.77(1.31-2.38)	0.0002	0.003	0
cg15426734	PARD6A	50855	TRUE	0.52(0.37-0.73)	0.0002	0.003	0
cg02813121	S100A12	6283	FALSE	1.94(1.37-2.75)	0.0002	0.003	0
cg05216141	ETV7	51513	TRUE	0.51(0.36-0.72)	0.0002	0.003	0
cg22140675	G6PC3	92579	TRUE	2.09(1.42-3.09)	0.0002	0.003	0
cg14298726	DKFZP686A10121	85865	TRUE	2(1.39-2.89)	0.0002	0.003	0
cg06645778	HSPC159	29094	TRUE	0.54(0.39-0.74)	0.0002	0.003	0
cg14269477	TRPV5	56302	FALSE	1.91(1.36-2.69)	0.0002	0.003	0
cg10523671	SLC15A2	6565	TRUE	0.51(0.36-0.73)	0.0002	0.003	0
cg08797194	UGCGL2	55757	TRUE	0.53(0.38-0.74)	0.0002	0.003	0
cg06785429	DCUN1D1	54165	FALSE	1.77(1.31-2.4)	0.0002	0.003	0
cg26306976	ITGB1BP1	9270	FALSE	0.55(0.4-0.75)	0.0002	0.003	0
cg05343453	LOC136263	136263	FALSE	0.49(0.33-0.71)	0.0002	0.003	0
cg27588902	GNMT	27232	TRUE	0.56(0.41-0.76)	0.0002	0.003	0
cg15261665	LTF	4057	TRUE	0.56(0.41-0.76)	0.0002	0.003	0
cg10213812	FOXN1	8456	FALSE	1.84(1.33-2.55)	0.0002	0.003	0
cg11719283	ZNF574	64763	FALSE	0.52(0.37-0.73)	0.0002	0.003	0
cg07031996	MTF1	4520	TRUE	0.54(0.39-0.75)	0.0002	0.003	0
cg19764555	AHNAK	79026	TRUE	0.5(0.35-0.72)	0.0002	0.003	−1
cg06394229	LGALS4	3960	FALSE	0.56(0.42-0.76)	0.0002	0.003	0
cg13931228	MPP6	51678	TRUE	0.53(0.38-0.74)	0.0002	0.003	−1

cg10645113	LOC349236	349236	FALSE	1.99(1.39-2.86)	0.0002	0.003	0
cg10681065	TFR2	7036	FALSE	0.56(0.42-0.76)	0.0002	0.004	−1
cg23219570	FGF23	8074	FALSE	0.53(0.38-0.74)	0.0002	0.004	0
cg18338293	BBS1	582	TRUE	0.53(0.39-0.74)	0.0002	0.004	0
cg15913671	TMEM105	284186	FALSE	1.86(1.34-2.57)	0.0002	0.004	0
cg13739417	IL8RB	3579	FALSE	0.43(0.28-0.67)	0.0002	0.004	0
cg12998614	KISS1R	84634	TRUE	0.54(0.4-0.75)	0.0002	0.004	0
cg15484375	SAA1	6288	FALSE	0.53(0.38-0.74)	0.0002	0.004	0
cg14722162	C5orf20	140947	FALSE	1.94(1.37-2.76)	0.0002	0.004	0
cg03019000	TEX264	51368	FALSE	1.85(1.34-2.57)	0.0002	0.004	0
cg21770617	FAM3D	131177	FALSE	1.99(1.38-2.85)	0.0002	0.004	0
cg02345317	NLGN3	54413	TRUE	0.54(0.4-0.74)	0.0002	0.004	0
cg01405107	HOXB5	3215	TRUE	0.55(0.4-0.76)	0.0002	0.004	−1
cg22585269	CHEK2	11200	TRUE	1.9(1.35-2.66)	0.0002	0.004	0
cg04870470	EXTL2	2135	FALSE	0.55(0.4-0.76)	0.0002	0.004	0
cg06244417	FCN1	2219	FALSE	0.54(0.39-0.75)	0.0002	0.004	0
cg15659828	CHID1	66005	TRUE	0.53(0.38-0.74)	0.0002	0.004	0
cg06310844	PDCD5	9141	TRUE	2.06(1.41-3.02)	0.0002	0.004	0
cg11804789	CST7	8530	FALSE	1.84(1.34-2.54)	0.0002	0.004	0
cg22268164	TRHR	7201	FALSE	1.8(1.32-2.45)	0.0002	0.004	0
cg05890019	WDFY3	23001	TRUE	0.49(0.34-0.72)	0.0002	0.004	0
cg14321743	PLA2G2D	26279	FALSE	1.84(1.33-2.54)	0.0002	0.004	0
cg04126866	C10orf99	387695	FALSE	1.9(1.35-2.68)	0.0002	0.004	0
cg21182407	BPHL	670	TRUE	0.51(0.35-0.73)	0.0002	0.004	−1
cg10451565	GPR77	27202	FALSE	0.54(0.38-0.75)	0.0002	0.004	0
cg13435381	LGR5	8549	TRUE	0.53(0.38-0.74)	0.0002	0.004	0
cg06200697	CTNND1	1500	FALSE	0.56(0.41-0.76)	0.0002	0.004	0

cg10922280	DPEP2	64174	FALSE	1.82(1.32-2.49)	0.0002	0.004	0
cg14823162	POU3F2	5454	TRUE	0.51(0.36-0.73)	0.0002	0.004	0
cg23732182	C21orf84	114038	FALSE	0.55(0.4-0.75)	0.0002	0.004	0
cg07510080	HIF1AN	55662	TRUE	1.93(1.36-2.74)	0.0002	0.004	0
cg20879959	HLA-A	3105	TRUE	0.54(0.39-0.75)	0.0002	0.004	0
cg13669740	VSIG9	201633	FALSE	1.91(1.35-2.69)	0.0002	0.004	0
cg10533434	SERPINB3	6317	FALSE	0.56(0.41-0.76)	0.0002	0.004	0
cg11435943	SERPINB12	89777	FALSE	1.88(1.35-2.61)	0.0002	0.004	0
cg02417408	PPM1M	132160	FALSE	1.86(1.34-2.59)	0.0002	0.004	0
cg06285340	CYP11A1	1583	FALSE	1.87(1.34-2.62)	0.0002	0.004	0
cg12582959	HSU79303	29903	TRUE	0.54(0.39-0.75)	0.0002	0.004	0
cg02332525	GRM7	2917	TRUE	0.55(0.4-0.75)	0.0002	0.004	0
cg15775914	CHML	1122	FALSE	1.85(1.33-2.58)	0.0002	0.004	1
cg00840516	HYAL2	8692	FALSE	0.56(0.42-0.77)	0.0002	0.004	0
cg24588599	C12orf24	29902	TRUE	1.83(1.33-2.51)	0.0002	0.004	0
cg21671476	MYL9	10398	TRUE	0.55(0.4-0.76)	0.0002	0.004	0
cg18485955	TNFRSF17	608	FALSE	0.54(0.39-0.75)	0.0002	0.004	0
cg13929328	FLJ46831	399823	TRUE	0.54(0.39-0.74)	0.0002	0.004	0
cg01593886	COL1A1	1277	TRUE	0.53(0.38-0.75)	0.0002	0.004	0
cg20126106	TAGLN3	29114	TRUE	0.54(0.39-0.75)	0.0002	0.004	0
cg08611714	KRTHA2	3882	FALSE	1.8(1.32-2.45)	0.0002	0.004	0
cg21949305	ADORA2A	135	FALSE	0.55(0.4-0.76)	0.0002	0.004	0
cg15526708	TGFBR1	7046	TRUE	0.55(0.39-0.76)	0.0002	0.004	0
cg04541607	CRYBB1	1414	FALSE	0.55(0.4-0.76)	0.0002	0.004	0
cg02431964	MARCO	8685	FALSE	0.48(0.33-0.71)	0.0002	0.004	0
cg21825364	VCY	9084	TRUE	0.51(0.36-0.73)	0.0002	0.004	0
cg12730381	YWHAG	7532	TRUE	1.96(1.39-2.77)	0.0002	0.004	0

cg04138756	SPRR3	6707	FALSE	1.95(1.37-2.78)	0.0002	0.004	0
cg15195412	CX3CL1	6376	FALSE	0.53(0.38-0.75)	0.0002	0.004	−1
cg14795968	ACADL	33	TRUE	0.55(0.4-0.75)	0.0002	0.004	0
cg20557202	SLC5A5	6528	TRUE	0.52(0.37-0.74)	0.0002	0.004	0
cg04856043	HS1BP3	64342	TRUE	0.55(0.4-0.76)	0.0002	0.004	0
cg05294455	MYL4	4635	FALSE	1.87(1.34-2.61)	0.0002	0.004	0
cg13555884	CTGLF1	119016	FALSE	0.53(0.38-0.75)	0.0002	0.004	0
cg14132995	SLC35A2	7355	TRUE	1.78(1.31-2.42)	0.0002	0.004	0
cg08603768	WNT8A	7478	FALSE	1.88(1.35-2.63)	0.0002	0.004	0
cg15278948	ZFAND2B	130617	TRUE	1.82(1.32-2.51)	0.0002	0.004	0
cg21440587	AIF1	199	FALSE	0.53(0.38-0.75)	0.0002	0.004	0
cg19005210	TREML2	79865	FALSE	0.54(0.39-0.75)	0.0002	0.004	0
cg15853125	TIAM1	7074	TRUE	1.81(1.32-2.49)	0.0002	0.004	0
cg11025793	STX10	8677	TRUE	1.88(1.34-2.64)	0.0002	0.004	0
cg17250929	S100A5	6276	FALSE	1.79(1.31-2.45)	0.0002	0.004	0
cg07435592	DCBLD2	131566	TRUE	0.55(0.4-0.76)	0.0002	0.004	0
cg21697134	FN3K	64122	TRUE	0.55(0.4-0.76)	0.0002	0.004	0
cg10784821	COQ6	51004	FALSE	0.39(0.23-0.64)	0.0002	0.004	0
cg26079320	POGK	57645	TRUE	0.5(0.34-0.73)	0.0002	0.004	0
cg24134767	HTR3A	3359	FALSE	1.86(1.33-2.59)	0.0002	0.004	0
cg24697031	EVPL	2125	FALSE	0.56(0.41-0.77)	0.0002	0.004	−1
cg20673481	KCNS3	3790	TRUE	0.53(0.38-0.74)	0.0002	0.004	0
cg06616245	GLI1	2735	FALSE	1.83(1.32-2.55)	0.0002	0.004	0
cg02385474	PCNXL2	80003	FALSE	1.83(1.32-2.53)	0.0002	0.004	0
cg12181621	HIST1H3I	8354	TRUE	1.98(1.39-2.82)	0.0002	0.004	0
cg14436426	OPRL1	4987	FALSE	0.56(0.41-0.77)	0.0002	0.004	−1
cg01909245	LSP1	4046	FALSE	1.79(1.31-2.44)	0.0002	0.004	0

cg09879797	WSB2	55884	TRUE	0.55(0.4-0.75)	0.0002	0.004	0
cg04586023	HDAC10	83933	FALSE	0.56(0.4-0.76)	0.0002	0.004	0
cg04794268	CYHR1	50626	FALSE	0.55(0.4-0.76)	0.0003	0.004	−1
cg20098659	CLEC9A	283420	TRUE	0.56(0.42-0.77)	0.0003	0.004	0
cg17264470	FGF21	26291	FALSE	1.88(1.34-2.65)	0.0003	0.004	0
cg01063813	STAT6	6778	FALSE	1.87(1.33-2.64)	0.0003	0.004	0
cg16112945	ADAMTS13	11093	FALSE	1.88(1.34-2.64)	0.0003	0.004	0
cg00077457	ZNF326	284695	TRUE	0.53(0.38-0.75)	0.0003	0.004	0
cg19145398	FKHL18	2307	FALSE	1.84(1.33-2.57)	0.0003	0.004	0
cg08005849	HGF	3082	FALSE	0.51(0.36-0.74)	0.0003	0.004	0
cg20050826	K6IRS2	140807	TRUE	0.54(0.39-0.76)	0.0003	0.004	0
cg14870271	LGALS3BP	3959	FALSE	1.83(1.32-2.52)	0.0003	0.005	0
cg22598563	P4HA2	8974	TRUE	0.56(0.41-0.77)	0.0003	0.005	0
cg16545105	CRHBP	1393	FALSE	0.56(0.41-0.77)	0.0003	0.005	0
cg23815306	TRIP13	9319	TRUE	0.52(0.37-0.74)	0.0003	0.005	0
cg00566759	DSC2	1824	TRUE	0.56(0.41-0.77)	0.0003	0.005	0
cg00095526	CACNA1S	779	FALSE	0.55(0.4-0.76)	0.0003	0.005	0
cg12739034	PTGER3	5733	TRUE	0.52(0.37-0.73)	0.0003	0.005	0
cg01723747	C16orf34	90861	TRUE	1.92(1.35-2.74)	0.0003	0.005	0
cg15057581	PTPNS1	140885	TRUE	0.52(0.37-0.74)	0.0003	0.005	0
cg23189044	KCNE3	10008	TRUE	0.54(0.38-0.75)	0.0003	0.005	0
cg24727203	PPP1R3B	79660	TRUE	0.55(0.4-0.76)	0.0003	0.005	0
cg01130192	ACO1	48	TRUE	0.55(0.4-0.76)	0.0003	0.005	0
cg15213605	SF3B3	23450	TRUE	1.92(1.35-2.74)	0.0003	0.005	0
cg23282674	IL20	50604	FALSE	0.53(0.38-0.75)	0.0003	0.005	0
cg24240626	REG3A	5068	FALSE	1.92(1.36-2.73)	0.0003	0.005	0
cg00717862	DNAJB11	51726	TRUE	1.87(1.34-2.61)	0.0003	0.005	0

cg24411312	ST14	6768	FALSE	1.85(1.33-2.57)	0.0003	0.005	0
cg03251079	HMGA2	8091	TRUE	0.42(0.26-0.66)	0.0003	0.005	0
cg03991326	RPL7	6129	TRUE	1.89(1.34-2.68)	0.0003	0.005	0
cg11879514	SLC16A6	9120	TRUE	1.9(1.34-2.69)	0.0003	0.005	0
cg27207274	MYC	4609	TRUE	1.87(1.33-2.64)	0.0003	0.005	0
cg19337279	MPL	4352	FALSE	0.56(0.41-0.77)	0.0003	0.005	0
cg04478795	SMO	6608	TRUE	0.52(0.37-0.74)	0.0003	0.005	0
cg21627181	SLC17A4	10050	FALSE	1.81(1.31-2.5)	0.0003	0.005	0
cg18081258	NDRG2	57447	TRUE	0.55(0.4-0.76)	0.0003	0.005	−1
cg11558474	TMEM2	23670	TRUE	1.98(1.36-2.87)	0.0003	0.005	0
cg01234133	COL1A1	1277	TRUE	0.46(0.3-0.71)	0.0003	0.005	0
cg01646665	TMEM79	84283	FALSE	1.77(1.3-2.42)	0.0003	0.005	0
cg17118262	CCL1	6346	FALSE	0.54(0.39-0.76)	0.0003	0.005	0
cg19391527	PNOC	5368	FALSE	1.73(1.28-2.33)	0.0003	0.005	0
cg23917399	TNFAIP8	25816	TRUE	0.54(0.39-0.76)	0.0003	0.005	0
cg02672493	TMEM22	80723	TRUE	0.56(0.4-0.77)	0.0003	0.005	0
cg21491308	TEX101	83639	FALSE	0.57(0.41-0.77)	0.0003	0.005	0
cg20648149	SYNE2	23224	TRUE	0.57(0.42-0.77)	0.0003	0.005	0
cg00431050	ELOVL3	83401	TRUE	0.55(0.4-0.76)	0.0003	0.005	0
cg27319898	FLJ32110	219578	TRUE	0.55(0.4-0.76)	0.0003	0.005	0
cg16470760	CD4	920	FALSE	1.79(1.3-2.47)	0.0003	0.005	0
cg12910797	HOXB3	3213	TRUE	0.53(0.37-0.75)	0.0003	0.005	−1
cg10559803	RALGPS2	55103	TRUE	0.55(0.4-0.77)	0.0003	0.005	0
cg14170423	RASGRP2	10235	TRUE	0.51(0.35-0.74)	0.0003	0.005	0
cg06785822	FLJ25102	348738	FALSE	0.55(0.39-0.76)	0.0003	0.005	0
cg07717632	FLJ20245	54863	TRUE	0.53(0.37-0.75)	0.0003	0.005	0
cg25511807	MMP7	4316	FALSE	0.56(0.41-0.77)	0.0003	0.005	0

cg04425624	TNF	7124	FALSE	0.55(0.4-0.76)	0.0003	0.005	−1
cg22202141	FCGR3A	2214	FALSE	2.05(1.4-3)	0.0003	0.005	0
cg23807646	SLC26A8	116369	FALSE	0.57(0.42-0.78)	0.0003	0.005	0
cg18139900	ZNF140	7699	TRUE	1.97(1.36-2.86)	0.0003	0.005	0
cg18787975	CTSB	1508	TRUE	0.55(0.4-0.77)	0.0003	0.005	0
cg23813257	IL32	9235	FALSE	1.78(1.3-2.44)	0.0003	0.005	0
cg18034859	MYLK2	85366	FALSE	0.55(0.39-0.76)	0.0003	0.005	0
cg21184174	NGFB	4803	TRUE	0.54(0.38-0.75)	0.0003	0.005	0
cg18119407	CFLAR	8837	TRUE	1.8(1.3-2.49)	0.0003	0.005	0
cg19006008	F2RL3	9002	FALSE	0.55(0.4-0.76)	0.0003	0.005	0
cg21522797	PLCG2	5336	FALSE	1.82(1.31-2.53)	0.0003	0.005	0
cg02712878	FAIM	55179	TRUE	0.58(0.43-0.78)	0.0003	0.005	0
cg26538442	FLJ21736	79984	FALSE	1.94(1.35-2.8)	0.0003	0.005	0
cg27634151	GPR83	10888	TRUE	0.47(0.31-0.7)	0.0003	0.005	0
cg22995106	COG4	25839	TRUE	1.81(1.31-2.5)	0.0003	0.005	0
cg12262564	IQGAP3	128239	TRUE	0.54(0.39-0.76)	0.0003	0.005	0
cg18107072	CGI-38	51673	TRUE	0.54(0.38-0.75)	0.0003	0.005	0
cg02719245	DICER1	23405	TRUE	2.04(1.38-3.01)	0.0003	0.005	0
cg11319389	C20orf100	84969	TRUE	1.82(1.31-2.53)	0.0003	0.005	0
cg16301617	TMC6	11322	FALSE	1.85(1.32-2.6)	0.0003	0.005	0
cg23306832	KIAA0999	23387	TRUE	1.87(1.32-2.65)	0.0003	0.005	0
cg18661868	FES	2242	FALSE	0.56(0.4-0.77)	0.0003	0.005	0
cg07267296	HMBOX1	79618	TRUE	0.54(0.38-0.76)	0.0003	0.005	0
cg21850069	TRIM41	90933	TRUE	1.77(1.3-2.42)	0.0003	0.005	0
cg23571857	BIRC4BP	54739	FALSE	0.58(0.43-0.78)	0.0003	0.005	0
cg10891879	CASZ1	54897	TRUE	1.78(1.3-2.44)	0.0003	0.005	0
cg03218374	ANGPT4	51378	FALSE	1.81(1.31-2.52)	0.0003	0.005	0

cg12091944	BARD1	580	TRUE	1.91(1.33-2.74)	0.0003	0.005	0
cg13759328	CDH13	1012	TRUE	0.4(0.24-0.66)	0.0003	0.005	0
cg17827767	LRRC21	26103	FALSE	1.88(1.33-2.66)	0.0003	0.005	0
cg22967298	PCNP	57092	TRUE	1.89(1.33-2.68)	0.0003	0.005	0
cg22131691	PDE1C	5137	TRUE	0.57(0.42-0.77)	0.0003	0.006	0
cg03693099	CEL	1056	FALSE	0.57(0.42-0.77)	0.0003	0.006	0
cg03439703	PDE3B	5140	TRUE	1.77(1.29-2.42)	0.0003	0.006	0
cg00987379	ZNF395	55893	TRUE	0.55(0.39-0.77)	0.0003	0.006	0
cg07973246	SRGAP1	57522	TRUE	0.53(0.37-0.75)	0.0003	0.006	0
cg12703269	PSTPIP1	9051	FALSE	1.78(1.29-2.45)	0.0003	0.006	0
cg14380517	BTG3	10950	TRUE	0.56(0.4-0.77)	0.0004	0.006	0
cg04726446	MGC16372	92749	TRUE	0.55(0.4-0.76)	0.0004	0.006	0
cg17850932	IL2	3558	FALSE	1.73(1.28-2.34)	0.0004	0.006	0
cg03460527	F3	2152	TRUE	0.54(0.38-0.75)	0.0004	0.006	0
cg10670077	INSR	3643	TRUE	0.54(0.38-0.76)	0.0004	0.006	0
cg16414852	SULT1B1	27284	FALSE	1.84(1.31-2.58)	0.0004	0.006	0
cg23231873	USP32	84669	TRUE	1.85(1.32-2.59)	0.0004	0.006	0
cg19372178	TMEM16G	50636	FALSE	1.8(1.3-2.48)	0.0004	0.006	0
cg08897388	LAMA4	3910	FALSE	0.54(0.38-0.76)	0.0004	0.006	0
cg24793903	SCRN2	90507	TRUE	1.75(1.28-2.38)	0.0004	0.006	0
cg08552999	ZNF644	84146	TRUE	1.79(1.31-2.46)	0.0004	0.006	0
cg22436229	PDIK1L	149420	TRUE	0.54(0.38-0.76)	0.0004	0.006	−1
cg22253036	DDX6	1656	TRUE	1.9(1.33-2.73)	0.0004	0.006	0
cg19815589	LOC220070	220070	FALSE	0.54(0.39-0.76)	0.0004	0.006	0
cg23320649	C3orf18	51161	FALSE	0.58(0.43-0.79)	0.0004	0.006	−1
cg02331561	ABCA3	21	TRUE	0.45(0.29-0.7)	0.0004	0.006	0
cg10822172	CREB5	9586	FALSE	0.58(0.42-0.78)	0.0004	0.006	−1

cg01074640	IFNA17	3451	FALSE	1.87(1.32-2.64)	0.0004	0.006	0
cg10624445	CNGB1	1258	FALSE	1.87(1.32-2.64)	0.0004	0.006	0
cg00294382	IL23A	51561	FALSE	1.98(1.35-2.89)	0.0004	0.006	0
cg07349094	AFF3	3899	TRUE	1.73(1.27-2.35)	0.0004	0.006	0
cg19356324	SLC7A6OS	84138	TRUE	0.54(0.38-0.76)	0.0004	0.006	0
cg14600885	MYOZ3	91977	FALSE	1.79(1.3-2.46)	0.0004	0.006	0
cg21264055	PRR3	80742	TRUE	0.56(0.41-0.78)	0.0004	0.006	0
cg25687894	ACLY	47	TRUE	0.55(0.39-0.77)	0.0004	0.006	0
cg16545079	PER1	5187	TRUE	0.53(0.37-0.75)	0.0004	0.006	0
cg16725130	MMP19	4327	FALSE	0.57(0.42-0.78)	0.0004	0.006	0
cg08418332	CCL19	6363	FALSE	0.59(0.44-0.79)	0.0004	0.006	0
cg00651216	HSP90AB1	3326	TRUE	0.54(0.38-0.76)	0.0004	0.006	−1
cg21161070	RACGAP1	29127	TRUE	1.87(1.32-2.65)	0.0004	0.006	0
cg24926042	KRTHB2	3888	FALSE	1.78(1.29-2.46)	0.0004	0.006	0
cg23978322	FHL5	9457	FALSE	0.56(0.41-0.77)	0.0004	0.006	0
cg02810134	TNNC2	7125	FALSE	1.82(1.31-2.54)	0.0004	0.006	0
cg00661485	FOXI1	2299	TRUE	0.52(0.36-0.74)	0.0004	0.006	0
cg02415431	IGLL1	3543	FALSE	0.56(0.4-0.78)	0.0004	0.006	0
cg16008138	RNF190	162333	TRUE	1.91(1.33-2.73)	0.0004	0.006	1
cg23749046	GPR61	83873	FALSE	1.84(1.31-2.59)	0.0004	0.006	0
cg20614736	CCDC74B	91409	FALSE	0.51(0.35-0.74)	0.0004	0.006	0
cg05839235	NPR3	4883	TRUE	0.54(0.39-0.76)	0.0004	0.006	0
cg06187947	GULP1	51454	TRUE	0.54(0.38-0.76)	0.0004	0.006	0
cg14366490	TXNL6	115861	FALSE	1.78(1.29-2.45)	0.0004	0.006	0
cg06557606	ATP6V1H	51606	TRUE	1.82(1.32-2.52)	0.0004	0.006	0
cg10506318	mimitin	91942	FALSE	0.55(0.4-0.77)	0.0004	0.006	0
cg27446185	VTCN1	79679	FALSE	1.75(1.28-2.38)	0.0004	0.006	0

cg07103493	SLC27A6	28965	TRUE	0.56(0.4-0.77)	0.0004	0.006	0
cg13265003	SLC37A1	54020	FALSE	0.58(0.43-0.78)	0.0004	0.006	0
cg06762858	PILRA	29992	FALSE	0.57(0.42-0.78)	0.0004	0.007	0
cg19713460	SYNGR1	9145	TRUE	0.57(0.42-0.78)	0.0004	0.007	0
cg27360098	ELN	2006	TRUE	0.56(0.4-0.78)	0.0004	0.007	−1
cg20267005	TYROBP	7305	FALSE	0.56(0.41-0.78)	0.0004	0.007	−1
cg22418909	SFRP1	6422	TRUE	0.53(0.38-0.75)	0.0004	0.007	1
cg05800321	LY6D	8581	TRUE	1.75(1.28-2.4)	0.0004	0.007	0
cg15686608	DAB2IP	153090	TRUE	0.53(0.37-0.75)	0.0004	0.007	0
cg12707233	WNK3	65267	TRUE	0.54(0.38-0.76)	0.0004	0.007	0
cg21301440	CYGB	114757	TRUE	0.56(0.4-0.77)	0.0004	0.007	0
cg24334983	ANXA11	311	TRUE	1.83(1.3-2.57)	0.0004	0.007	0
cg23460697	UBN1	29855	FALSE	0.49(0.32-0.73)	0.0004	0.007	0
cg21494776	ICAM4	3386	TRUE	0.55(0.4-0.77)	0.0004	0.007	0
cg23651356	RGS17	26575	TRUE	0.58(0.42-0.79)	0.0004	0.007	0
cg01663469	C3orf15	89876	FALSE	0.56(0.4-0.77)	0.0004	0.007	0
cg26309951	MORF4L2	9643	TRUE	1.78(1.28-2.46)	0.0004	0.007	0
cg15118503	AHSA2	130872	TRUE	1.84(1.31-2.57)	0.0004	0.007	0
cg03139377	SLC6A12	6539	FALSE	0.58(0.42-0.79)	0.0004	0.007	0
cg00658007	C1orf36	343035	FALSE	0.57(0.41-0.78)	0.0004	0.007	0
cg13585240	ARIH2	10425	FALSE	1.82(1.3-2.56)	0.0004	0.007	0
cg19038540	EDG1	1901	TRUE	1.81(1.29-2.52)	0.0004	0.007	0
cg12966875	SLPI	6590	FALSE	0.57(0.41-0.78)	0.0005	0.007	0
cg22510943	LMNB2	84823	TRUE	1.77(1.28-2.45)	0.0005	0.007	0
cg05559445	CDKN1C	1028	TRUE	0.58(0.42-0.79)	0.0005	0.007	0
cg11102782	ISYNA1	51477	TRUE	0.56(0.4-0.78)	0.0005	0.007	0
cg12954718	USP6	9098	FALSE	1.75(1.28-2.41)	0.0005	0.007	0

cg21581873	PLEKHA6	22874	FALSE	0.56(0.4-0.77)	0.0005	0.007	0
cg01963696	ELA2	1991	TRUE	0.58(0.43-0.79)	0.0005	0.007	0
cg14885742	IFITM3	10410	TRUE	0.57(0.42-0.78)	0.0005	0.007	0
cg14841875	ATP1A1	476	TRUE	1.95(1.34-2.83)	0.0005	0.007	0
cg01124420	EDAR	10913	FALSE	1.73(1.28-2.35)	0.0005	0.007	0
cg00815605	ACOT2	10965	TRUE	0.59(0.44-0.8)	0.0005	0.007	0
cg09279263	TADA3L	10474	FALSE	1.81(1.29-2.54)	0.0005	0.007	0
cg22988566	WFDC10B	280664	FALSE	1.89(1.32-2.7)	0.0005	0.007	0
cg23902550	TIGD5	84948	TRUE	0.57(0.42-0.78)	0.0005	0.007	0
cg24506604	LOC144501	144501	FALSE	1.84(1.3-2.61)	0.0005	0.007	0
cg07076342	MEA1	4201	TRUE	1.78(1.28-2.46)	0.0005	0.007	0
cg27067618	CYP4F3	4051	FALSE	0.57(0.42-0.79)	0.0005	0.007	0
cg03167763	UBXD3	127733	TRUE	1.79(1.29-2.47)	0.0005	0.007	0
cg20557104	B3GALT7	374907	FALSE	0.57(0.41-0.78)	0.0005	0.007	0
cg22241124	CNGA3	1261	FALSE	1.74(1.27-2.37)	0.0005	0.007	0
cg18456149	NAPB	63908	FALSE	1.79(1.29-2.48)	0.0005	0.007	0
cg14450506	DDX56	54606	TRUE	0.58(0.43-0.79)	0.0005	0.007	0
cg14386061	DOK2	9046	FALSE	1.91(1.33-2.75)	0.0005	0.007	0
cg21366535	RSHL2	83861	FALSE	2.03(1.37-3.01)	0.0005	0.007	0
cg02988947	LIMD2	80774	TRUE	1.71(1.26-2.32)	0.0005	0.007	0
cg22972055	UNC84A	23353	TRUE	1.72(1.26-2.34)	0.0005	0.007	1
cg13234848	AUTS2	26053	TRUE	1.77(1.28-2.44)	0.0005	0.007	0
cg06392241	NUDT4	11163	TRUE	0.55(0.4-0.78)	0.0005	0.007	0
cg20795401	ITGA6	3655	TRUE	1.71(1.26-2.31)	0.0005	0.007	0
cg14154330	ARHGAP27	201176	FALSE	0.58(0.43-0.79)	0.0005	0.007	0
cg00346145	RPL34	6164	TRUE	1.8(1.3-2.51)	0.0005	0.007	0
cg24887211	C14orf166B	145497	FALSE	1.82(1.3-2.54)	0.0005	0.007	0

cg07548313	CD1C	911	FALSE	1.81(1.29-2.55)	0.0005	0.007	0
cg12080675	KIAA0676	23061	TRUE	0.53(0.37-0.76)	0.0005	0.007	0
cg22933847	MRGPRF	219928	FALSE	0.57(0.41-0.79)	0.0005	0.007	−1
cg24851490	RNASE2	6036	FALSE	0.56(0.4-0.78)	0.0005	0.007	0
cg08463061	RND3	390	TRUE	1.89(1.32-2.7)	0.0005	0.007	0
cg08157579	SERHL2	253190	TRUE	1.86(1.32-2.62)	0.0005	0.007	0
cg01599709	HBEGF	1839	TRUE	0.56(0.4-0.78)	0.0005	0.007	0
cg16652259	DLX1	1745	TRUE	0.54(0.39-0.76)	0.0005	0.007	0
cg23773532	GPRC5D	55507	FALSE	1.78(1.28-2.47)	0.0005	0.007	0
cg24904765	STAM2	10254	TRUE	1.91(1.33-2.76)	0.0005	0.007	0
cg11058932	TSGA13	114960	FALSE	0.59(0.44-0.8)	0.0005	0.007	0
cg25697314	IL26	55801	FALSE	1.77(1.28-2.45)	0.0005	0.007	0
cg01172899	SLC16A14	151473	FALSE	0.57(0.41-0.79)	0.0005	0.008	0
cg09072120	PRTFDC1	56952	TRUE	0.55(0.4-0.78)	0.0005	0.008	−1
cg10833576	ANKRA2	57763	TRUE	1.81(1.3-2.51)	0.0005	0.008	0
cg21418052	B3GTL	145173	TRUE	1.73(1.27-2.38)	0.0005	0.008	0
cg22181664	CLCA1	1179	FALSE	1.83(1.31-2.57)	0.0005	0.008	0
cg02347487	NALP14	338323	FALSE	1.73(1.26-2.38)	0.0005	0.008	0
cg21504918	GPX3	2878	TRUE	0.55(0.39-0.77)	0.0005	0.008	0
cg14023451	GPLD1	2822	FALSE	0.59(0.44-0.8)	0.0005	0.008	0
cg26985289	CLSTN1	22883	TRUE	0.57(0.41-0.78)	0.0005	0.008	0
cg16744741	PRKG2	5593	FALSE	0.57(0.42-0.79)	0.0005	0.008	−1
cg08729012	CMTM6	54918	TRUE	1.8(1.29-2.53)	0.0005	0.008	0
cg21057494	CLEC3B	7123	FALSE	1.77(1.29-2.44)	0.0005	0.008	0
cg16540259	NDST2	8509	TRUE	0.59(0.44-0.8)	0.0005	0.008	0
cg11959435	IFNA1	3439	FALSE	1.75(1.27-2.4)	0.0005	0.008	0
cg07671208	BTN2A1	11120	FALSE	0.58(0.43-0.79)	0.0005	0.008	0

cg18528640	MSTO1	55154	TRUE	1.69(1.26-2.28)	0.0005	0.008	0
cg25697050	INPP5D	3635	FALSE	0.53(0.37-0.76)	0.0005	0.008	0
cg21415698	EXPH5	23086	TRUE	1.76(1.27-2.43)	0.0005	0.008	0
cg12241297	HNRPA0	10949	TRUE	1.76(1.27-2.45)	0.0005	0.008	0
cg18992688	AVPR1B	553	TRUE	0.51(0.35-0.75)	0.0005	0.008	0
cg09674215	TWIST1	7291	TRUE	0.58(0.43-0.79)	0.0005	0.008	0
cg08731300	FZD8	8325	TRUE	0.55(0.39-0.77)	0.0005	0.008	0
cg15020645	APC	324	FALSE	0.57(0.42-0.79)	0.0005	0.008	0
cg26540515	ANGPT4	51378	FALSE	0.57(0.41-0.79)	0.0006	0.008	0
cg03822159	SETD8	387893	TRUE	0.59(0.43-0.8)	0.0006	0.008	0
cg21892708	PDIA6	10130	TRUE	0.5(0.33-0.75)	0.0006	0.008	0
cg14306534	ZBTB2	57621	TRUE	1.82(1.3-2.54)	0.0006	0.008	0
cg11635563	PIG38	55068	FALSE	0.58(0.42-0.79)	0.0006	0.008	0
cg19798224	C14orf68	283600	FALSE	1.74(1.27-2.38)	0.0006	0.008	0
cg15319451	TMEM51	55092	TRUE	0.51(0.35-0.75)	0.0006	0.008	0
cg19093820	GPR156	165829	FALSE	1.78(1.29-2.47)	0.0006	0.008	0
cg01356829	IL12RB2	3595	TRUE	0.6(0.44-0.8)	0.0006	0.008	0
cg14974772	FBLN5	10516	TRUE	0.57(0.41-0.78)	0.0006	0.008	0
cg01731685	IL17R	23765	TRUE	0.56(0.4-0.78)	0.0006	0.008	0
cg21570220	SLC11A2	4891	TRUE	2.1(1.37-3.21)	0.0006	0.008	0
cg11808757	TUBGCP6	85378	TRUE	1.77(1.27-2.46)	0.0006	0.008	0
cg15552238	EMR3	84658	FALSE	0.6(0.45-0.81)	0.0006	0.008	0
cg26331247	FLJ33706	284805	FALSE	1.79(1.3-2.46)	0.0006	0.008	0
cg27563778	C4orf17	84103	FALSE	0.55(0.39-0.78)	0.0006	0.008	0
cg16142218	CHMP7	91782	FALSE	1.84(1.3-2.61)	0.0006	0.008	0
cg08804892	TRAK1	22906	FALSE	1.79(1.28-2.49)	0.0006	0.008	0
cg27470554	FCGR2A	2212	FALSE	0.56(0.41-0.78)	0.0006	0.008	0

cg04311964	LYPD2	137797	FALSE	1.75(1.28-2.4)	0.0006	0.008	0
cg26955850	OXT	5020	TRUE	0.55(0.39-0.78)	0.0006	0.008	0
cg19258882	ERBB3	2065	TRUE	0.57(0.41-0.79)	0.0006	0.008	−1
cg21614231	BAG1	573	FALSE	0.57(0.41-0.78)	0.0006	0.008	0
cg05678175	TRIM28	10155	TRUE	1.67(1.25-2.24)	0.0006	0.008	0
cg10644361	MIPOL1	145282	TRUE	0.52(0.36-0.76)	0.0006	0.008	0
cg11787522	STRA6	64220	FALSE	1.75(1.27-2.39)	0.0006	0.008	0
cg17754680	NPC1L1	29881	FALSE	1.77(1.27-2.46)	0.0006	0.008	0
cg08766742	SLC25A19	60386	TRUE	1.68(1.25-2.26)	0.0006	0.008	0
cg16749578	NR2F6	2063	FALSE	0.57(0.41-0.78)	0.0006	0.008	0
cg11003133	AIM2	9447	FALSE	1.72(1.26-2.35)	0.0006	0.008	0
cg04968426	PPP1R14D	54866	FALSE	1.8(1.29-2.5)	0.0006	0.008	0
cg09551916	CFHR2	3080	FALSE	1.71(1.25-2.33)	0.0006	0.008	0
cg06334284	PPAP2B	8613	TRUE	0.56(0.4-0.78)	0.0006	0.008	0
cg23663653		3500	TRUE	1.82(1.29-2.57)	0.0006	0.008	0
cg13548361	PSD	5662	TRUE	0.54(0.38-0.77)	0.0006	0.008	0
cg15503752	ST6GALNAC1	55808	FALSE	1.71(1.25-2.34)	0.0006	0.008	0
cg08411049	SERPINB5	5268	TRUE	1.9(1.32-2.75)	0.0006	0.008	0
cg26220350	PLTP	5360	TRUE	0.57(0.41-0.78)	0.0006	0.008	0
cg24659201	GPR39	2863	TRUE	0.58(0.42-0.79)	0.0006	0.009	0
cg13962355	BTRC	8945	TRUE	1.88(1.31-2.71)	0.0006	0.009	0
cg18370227	KRTHA3A	3883	FALSE	0.56(0.4-0.78)	0.0006	0.009	0
cg05279864	ABCB4	5244	FALSE	1.73(1.26-2.38)	0.0006	0.009	0
cg06462291	NT5DC3	51559	FALSE	0.55(0.39-0.77)	0.0006	0.009	0
cg06117855	CLEC3B	7123	FALSE	0.57(0.41-0.79)	0.0006	0.009	0
cg25764570	HLA-DRA	3122	FALSE	0.6(0.45-0.8)	0.0006	0.009	0
cg03602500	FLJ00060	90011	TRUE	1.72(1.25-2.37)	0.0006	0.009	1

cg01830294	WNT2	7472	TRUE	0.56(0.41-0.78)	0.0006	0.009	0
cg06836736	ME1	4199	TRUE	0.5(0.34-0.74)	0.0006	0.009	0
cg01305421	IGF1	3479	FALSE	0.57(0.41-0.79)	0.0006	0.009	0
cg12006284	WT1	7490	TRUE	1.69(1.25-2.28)	0.0006	0.009	0
cg01997953	CXorf21	80231	FALSE	1.99(1.34-2.96)	0.0006	0.009	0
cg20496643	RASL10B	91608	TRUE	0.59(0.44-0.8)	0.0006	0.009	0
cg06885782	KCNQ4	9132	TRUE	0.59(0.43-0.8)	0.0007	0.009	−1
cg05330360	ZPBP2	124626	TRUE	1.69(1.24-2.29)	0.0007	0.009	1
cg20864636	GARNL3	84253	FALSE	0.53(0.37-0.77)	0.0007	0.009	0
cg13015534	ST6GALNAC1	55808	FALSE	1.71(1.25-2.34)	0.0007	0.009	0
cg12356261	NES	10763	TRUE	0.56(0.4-0.78)	0.0007	0.009	0
cg03993463	KCNJ15	3772	FALSE	1.85(1.29-2.65)	0.0007	0.009	0
cg14732540	BRDT	676	TRUE	1.69(1.25-2.3)	0.0007	0.009	0
cg02479575	C19orf30	284424	TRUE	0.57(0.41-0.79)	0.0007	0.009	0
cg23988567		3502	FALSE	1.83(1.29-2.6)	0.0007	0.009	0
cg25475171	SV2B	9899	TRUE	1.78(1.29-2.46)	0.0007	0.009	0
cg18089000	GBGT1	26301	FALSE	0.58(0.42-0.8)	0.0007	0.009	0
cg06207804	ARTN	9048	TRUE	0.58(0.42-0.79)	0.0007	0.009	0
cg03972838	FLJ20232	54471	TRUE	0.57(0.41-0.79)	0.0007	0.009	−1
cg05275231	SLC25A32	81034	TRUE	1.8(1.28-2.54)	0.0007	0.009	0
cg02862362	ARG1	383	FALSE	0.59(0.43-0.8)	0.0007	0.009	0
cg24092253	YTHDF1	54915	TRUE	0.58(0.43-0.8)	0.0007	0.009	−1
cg10307548	SOD3	6649	FALSE	0.58(0.42-0.8)	0.0007	0.009	−1
cg13085976	FLJ20245	54863	FALSE	0.57(0.42-0.79)	0.0007	0.009	0
cg16983211	HOXC5	3222	TRUE	1.72(1.25-2.36)	0.0007	0.009	0
cg25421002	MMAA	166785	TRUE	1.69(1.24-2.3)	0.0007	0.009	1
cg19046959	COL8A2	1296	FALSE	0.55(0.39-0.78)	0.0007	0.009	0

cg12391921	ITGB1BP2	26548	FALSE	0.58(0.43-0.8)	0.0007	0.009	0
cg12240237	WBSCR23	80112	FALSE	1.77(1.27-2.48)	0.0007	0.009	0
cg15774495	GRB10	2887	TRUE	0.56(0.4-0.79)	0.0007	0.009	0
cg18627308	FLJ10661	55199	TRUE	0.57(0.41-0.79)	0.0007	0.009	0
cg26790059	MGC27121	408263	FALSE	1.79(1.28-2.51)	0.0007	0.01	0
cg15032239	CYFIP1	23191	TRUE	0.59(0.43-0.81)	0.0007	0.01	0
cg09207718	CYP1A2	1544	FALSE	1.91(1.31-2.79)	0.0007	0.01	0
cg16927136	RPL35A	6165	TRUE	1.7(1.25-2.32)	0.0007	0.01	0
cg01414934	SH3BGRL3	83442	FALSE	0.55(0.39-0.78)	0.0007	0.01	0
cg13473383	ZDHHC5	25921	TRUE	0.55(0.39-0.79)	0.0007	0.01	−1
cg01377755	ACP6	51205	TRUE	0.58(0.42-0.8)	0.0007	0.01	−1
cg06238491	LAIR1	3903	FALSE	0.55(0.39-0.78)	0.0007	0.01	0
cg01942127	CACNA1D	776	TRUE	0.56(0.4-0.78)	0.0007	0.01	0
cg09581098	SEPT6	23157	TRUE	1.67(1.24-2.26)	0.0007	0.01	0
cg20938359	SLC6A12	6539	FALSE	0.58(0.42-0.8)	0.0007	0.01	−1
cg27553955	KCNG3	170850	TRUE	1.76(1.26-2.45)	0.0007	0.01	1
cg22917487	CX3CR1	1524	FALSE	1.81(1.3-2.53)	0.0007	0.01	0
cg14667273	VWA1	64856	TRUE	0.56(0.4-0.78)	0.0007	0.01	0
cg18704047	PART1	25859	FALSE	1.7(1.25-2.33)	0.0007	0.01	0
cg18641050	BLOC1S2	282991	TRUE	2.05(1.34-3.13)	0.0007	0.01	0
cg26574610	VPREB3	29802	FALSE	1.78(1.27-2.5)	0.0007	0.01	0
cg24545967	SH2D3C	10044	FALSE	2.16(1.4-3.33)	0.0008	0.01	0
cg09095562	GTF3C5	9328	TRUE	0.59(0.43-0.8)	0.0008	0.01	0
cg00888479	SLC24A3	57419	TRUE	0.58(0.43-0.8)	0.0008	0.01	0
cg17215680	SIGLEC10	89790	FALSE	0.59(0.43-0.8)	0.0008	0.01	0
cg02265318	MOSC1	64757	TRUE	0.57(0.41-0.79)	0.0008	0.01	0
cg10409560	FLJ23657	152816	FALSE	1.74(1.26-2.41)	0.0008	0.01	0

cg09645888	ME3	10873	TRUE	0.54(0.37-0.77)	0.0008	0.01	0
cg06409153	ABCA5	23461	TRUE	1.81(1.28-2.56)	0.0008	0.01	0
cg17518962	GAL3ST4	79690	FALSE	0.59(0.43-0.8)	0.0008	0.01	0
cg25488021	EPB41L2	2037	FALSE	0.6(0.45-0.81)	0.0008	0.01	0
cg16935609	SRD5A1	6715	FALSE	0.6(0.44-0.81)	0.0008	0.01	0
cg25268718	PSME1	5720	FALSE	0.55(0.38-0.78)	0.0008	0.01	−1
cg23841186	SOAT2	8435	FALSE	0.6(0.44-0.81)	0.0008	0.01	−1
cg07052387	HIF1A	3091	TRUE	1.68(1.25-2.26)	0.0008	0.01	0
cg07389922	C17orf81	23587	FALSE	0.52(0.35-0.76)	0.0008	0.01	0
cg21301148	MYH6	4624	FALSE	1.68(1.24-2.25)	0.0008	0.01	0
cg04434339	ST6GAL2	84620	TRUE	0.53(0.37-0.77)	0.0008	0.01	0
cg13802966	CASP1	834	FALSE	1.7(1.25-2.32)	0.0008	0.01	0
cg08567916	SPRYD3	84926	TRUE	1.69(1.24-2.29)	0.0008	0.01	0
cg05546044	MAPK1	5594	TRUE	2.08(1.36-3.17)	0.0008	0.01	0
cg26024843	COL5A1	1289	TRUE	0.41(0.24-0.69)	0.0008	0.01	0
cg22511947	FN1	2335	TRUE	1.77(1.26-2.48)	0.0008	0.01	0
cg17819635	TCTEX1D1	200132	TRUE	0.57(0.41-0.79)	0.0008	0.01	0
cg03791917	BTK	695	FALSE	0.55(0.39-0.79)	0.0008	0.01	0
cg06000781	BST1	683	TRUE	0.57(0.41-0.79)	0.0008	0.01	0
cg08388746	C1orf83	127428	TRUE	1.71(1.24-2.34)	0.0008	0.01	0
cg11161417	SPACA3	124912	FALSE	1.68(1.24-2.28)	0.0008	0.01	0
cg24024214	BTNL8	79908	TRUE	2(1.33-3)	0.0008	0.01	0
cg03746685	TMEM126B	55863	FALSE	1.8(1.27-2.56)	0.0008	0.01	0
cg23756272	BCL2	596	TRUE	0.6(0.44-0.81)	0.0008	0.01	0
cg02873524	PAPPA	5069	TRUE	0.51(0.35-0.75)	0.0008	0.01	0
cg16670497	GSTM2	2946	TRUE	0.58(0.42-0.8)	0.0008	0.01	0
cg20339230	ST8SIA2	8128	TRUE	0.56(0.4-0.78)	0.0008	0.01	0

cg23743472	C11orf38	399967	FALSE	0.48(0.31-0.74)	0.0008	0.01	0
cg13383490	TSG101	7251	TRUE	1.67(1.23-2.26)	0.0008	0.01	0
cg13151102	KCNJ4	3761	TRUE	1.84(1.28-2.64)	0.0008	0.01	0
cg22443330	KIAA1018	22909	FALSE	0.57(0.41-0.79)	0.0008	0.01	0
cg12575181	PIGH	5283	TRUE	0.52(0.35-0.77)	0.0008	0.01	0
cg11504897	NRN1	51299	TRUE	1.72(1.25-2.37)	0.0008	0.01	0
cg21226225	TXNRD1	7296	TRUE	1.85(1.28-2.68)	0.0008	0.01	0
cg13265789	UNC5C	8633	TRUE	0.54(0.38-0.77)	0.0008	0.01	0
cg11260422	FOXA1	3169	TRUE	0.42(0.25-0.69)	0.0008	0.01	0
cg20675440	COG8	84342	FALSE	0.59(0.44-0.8)	0.0008	0.01	0
cg03469054	KIAA1944	121256	TRUE	0.57(0.42-0.79)	0.0008	0.01	0
cg08612871	GJB5	2709	FALSE	1.74(1.25-2.43)	0.0008	0.01	0
cg00459975	HMBOX1	79618	TRUE	0.59(0.43-0.8)	0.0008	0.01	0
cg12347740	MGC34647	146433	TRUE	0.57(0.4-0.79)	0.0008	0.01	0
cg18508125	FBXO16	157574	FALSE	1.68(1.24-2.28)	0.0008	0.01	0
cg09425611	CES1	1066	FALSE	1.71(1.24-2.36)	0.0008	0.01	0
cg05484458	GNB3	2784	FALSE	1.81(1.27-2.57)	0.0009	0.01	0
cg07288394	C19orf12	83636	TRUE	1.78(1.27-2.5)	0.0009	0.01	0
cg17055734	CRYGA	1418	TRUE	0.57(0.41-0.8)	0.0009	0.01	0
cg15297650	DKFZP566N034	81615	TRUE	0.58(0.42-0.8)	0.0009	0.01	−1
cg11227541	GNG5	2787	TRUE	1.68(1.23-2.3)	0.0009	0.01	0
cg17910564	VDAC3	7419	TRUE	1.82(1.28-2.58)	0.0009	0.01	0
cg25485913	NTF5	4909	FALSE	1.66(1.23-2.25)	0.0009	0.01	0
cg05869964	C5orf13	9315	FALSE	0.58(0.42-0.8)	0.0009	0.01	0
cg12431401	FLJ45909	126432	FALSE	1.75(1.25-2.45)	0.0009	0.01	0
cg26087862	ENSA	2029	TRUE	1.82(1.28-2.58)	0.0009	0.01	0
cg24496666	GIPC2	54810	TRUE	0.58(0.42-0.8)	0.0009	0.01	0

cg15301694	STRN3	29966	TRUE	0.58(0.41-0.8)	0.0009	0.01	0
cg08394377	RIMS3	9783	FALSE	0.59(0.44-0.81)	0.0009	0.01	0
cg18623836	RRM2	6241	TRUE	1.68(1.23-2.28)	0.0009	0.01	0
cg27544190	C21orf63	59271	TRUE	0.55(0.39-0.79)	0.0009	0.01	−1
cg23555120	NUAK1	9891	TRUE	0.57(0.41-0.79)	0.0009	0.01	0
cg03513363	DUSP15	128853	TRUE	0.58(0.42-0.8)	0.0009	0.01	0
cg14440934	ZDHHC1	29800	TRUE	1.76(1.25-2.46)	0.0009	0.01	0
cg21509097	LYPLA3	23659	TRUE	1.73(1.25-2.38)	0.0009	0.01	0
cg14313310	GLT25D2	23127	TRUE	0.58(0.42-0.81)	0.0009	0.01	0
cg22960284	PYGL	5836	TRUE	0.57(0.4-0.8)	0.0009	0.01	0
cg20427865	CX3CL1	6376	FALSE	0.54(0.38-0.78)	0.0009	0.01	0
cg17983307	DKFZp434B1231	91156	FALSE	1.71(1.25-2.35)	0.0009	0.01	0
cg24919884	ARHGEF16	27237	FALSE	1.74(1.26-2.42)	0.0009	0.01	0
cg23909633	IL24	11009	FALSE	1.71(1.24-2.36)	0.0009	0.01	0
cg07115820	EPX	8288	FALSE	1.7(1.24-2.33)	0.0009	0.01	0
cg24459563	CACNG1	786	TRUE	0.54(0.37-0.78)	0.0009	0.01	0
cg27529628	GAS2L3	283431	TRUE	0.57(0.41-0.79)	0.0009	0.01	0
cg18042806	MGC15875	85007	TRUE	1.68(1.23-2.3)	0.0009	0.01	0
cg19239342	REEP5	7905	TRUE	1.64(1.22-2.2)	0.0009	0.01	0
cg19111262	IGSF9	57549	FALSE	0.58(0.42-0.8)	0.0009	0.01	−1
cg08578641	DNAI1	27019	TRUE	0.57(0.41-0.8)	0.0009	0.01	0
cg13519373	IL8RA	3577	FALSE	1.83(1.28-2.62)	0.0009	0.01	0
cg17399166	CD1D	912	FALSE	1.8(1.27-2.56)	0.0009	0.01	0
cg27076812	TM7SF3	51768	TRUE	1.74(1.25-2.43)	0.0009	0.01	0
cg24784109	HIST1H3D	8351	TRUE	1.76(1.26-2.47)	0.0009	0.01	0
cg00754253	HRASLS5	117245	FALSE	0.57(0.41-0.8)	0.0009	0.01	0
cg15113803	RHO	6010	FALSE	1.7(1.24-2.33)	0.0009	0.01	0

cg17524886	CHFR	55743	TRUE	1.86(1.28-2.71)	0.0009	0.01	1
cg10523019	RHBDD1	84236	TRUE	1.7(1.23-2.34)	0.0009	0.01	1
cg25117362	C10orf77	79847	TRUE	1.7(1.24-2.35)	0.001	0.01	0
cg05501682	DKFZp686I15217	401232	TRUE	0.57(0.4-0.8)	0.001	0.01	−1
cg12079362	ATP6V0B	533	TRUE	1.8(1.26-2.56)	0.001	0.01	0
cg00955451	APBA2	321	FALSE	1.71(1.24-2.36)	0.001	0.01	0
cg25894551	FXYD2	486	FALSE	1.74(1.26-2.42)	0.001	0.01	0
cg26361928	G3BP	10146	TRUE	1.65(1.23-2.23)	0.001	0.01	0
cg03328804	UVRAG	7405	TRUE	1.76(1.26-2.47)	0.001	0.01	0
cg27524460	PTGER1	5731	FALSE	1.7(1.24-2.34)	0.001	0.01	0
cg20571908	MPHOSPH1	9585	TRUE	1.92(1.3-2.83)	0.001	0.01	0
cg19388557	GPR174	84636	FALSE	0.57(0.41-0.8)	0.001	0.01	0
cg06417225	PEBP1	5037	FALSE	1.77(1.26-2.47)	0.001	0.01	0
cg26164184	FCN2	2220	FALSE	1.75(1.26-2.43)	0.001	0.01	0
cg22638542	SEC22L3	9117	FALSE	0.57(0.41-0.8)	0.001	0.01	0
cg15056412	APBA2BP	63941	TRUE	0.57(0.41-0.8)	0.001	0.01	0
cg08659539	ZFYVE19	84936	FALSE	0.55(0.38-0.79)	0.001	0.01	0
cg02806777	PGLYRP1	8993	TRUE	0.57(0.41-0.8)	0.001	0.01	0
cg20530223	KLHL5	51088	TRUE	1.79(1.27-2.53)	0.001	0.01	0
cg21608489	POU2F2	5452	TRUE	1.69(1.24-2.3)	0.001	0.01	0
cg18006568	FLJ12056	79998	TRUE	0.58(0.42-0.8)	0.001	0.01	0
cg00237010	NINJ2	4815	FALSE	1.68(1.23-2.3)	0.001	0.01	0
cg05249393	LDLR	3949	TRUE	1.74(1.25-2.44)	0.001	0.01	0
cg19464016	PRDM1	639	TRUE	0.59(0.43-0.81)	0.001	0.01	0
cg03640944	KIAA1754	85450	FALSE	1.97(1.32-2.93)	0.001	0.01	0
cg25635352	B3GALT3	8706	TRUE	0.57(0.41-0.8)	0.001	0.01	0
cg09721427	HHEX	3087	TRUE	0.59(0.43-0.81)	0.001	0.01	0

cg05516537	TFDP1	7027	TRUE	0.59(0.43-0.81)	0.001	0.01	−1
cg26255848	FBL	2091	TRUE	1.81(1.27-2.58)	0.001	0.01	0
cg26754448	C12orf57	113246	TRUE	1.72(1.24-2.38)	0.001	0.01	0
cg25473396	MPG	4350	FALSE	0.58(0.42-0.8)	0.001	0.01	0
cg24214470	SERPINF1	5176	TRUE	0.59(0.43-0.8)	0.001	0.01	0
cg19192120	SSH3	54961	TRUE	0.57(0.41-0.8)	0.001	0.01	0
cg17468440	PPP2R1B	5519	TRUE	0.56(0.39-0.79)	0.001	0.01	0
cg26361780	CPB1	1360	FALSE	0.51(0.34-0.77)	0.001	0.01	0
cg19759064	PHKG1	5260	FALSE	0.56(0.4-0.8)	0.001	0.01	−1
cg16506346	OR1E1	8387	FALSE	0.56(0.4-0.8)	0.001	0.01	0
cg07136421	GPR89A	51463	TRUE	1.68(1.23-2.29)	0.001	0.01	0
cg08207256	DNM2	1785	TRUE	1.87(1.28-2.73)	0.001	0.01	0
cg10989326	EVI5	7813	FALSE	1.77(1.26-2.48)	0.001	0.01	0
cg22598028	ZNF660	285349	TRUE	0.57(0.41-0.79)	0.001	0.01	0
cg21517055	MGC11271	79173	TRUE	1.64(1.22-2.22)	0.001	0.01	0
cg25488206	RBM9	23543	FALSE	0.57(0.41-0.79)	0.001	0.01	0
cg10362591	SLC6A2	6530	TRUE	0.56(0.39-0.79)	0.001	0.01	0
cg25494064	TMOD1	7111	FALSE	0.59(0.43-0.81)	0.001	0.01	0
cg00135056	C3orf37	56941	TRUE	0.55(0.38-0.79)	0.001	0.01	0
cg24993443	SNRPN	6638	TRUE	0.6(0.45-0.82)	0.001	0.01	0
cg06917325	SLC22A8	9376	FALSE	1.72(1.24-2.38)	0.001	0.01	0
cg20357806	PPBP	5473	FALSE	1.66(1.22-2.26)	0.001	0.01	0
cg22552966	FLJ31196	146802	FALSE	1.98(1.31-2.99)	0.001	0.01	0
cg03247626	HINT3	135114	TRUE	1.68(1.23-2.28)	0.001	0.01	0
cg05338167	CALML4	91860	FALSE	0.59(0.44-0.81)	0.001	0.01	0
cg24959428	GBP6	163351	FALSE	0.59(0.43-0.8)	0.001	0.01	0
cg15439862	DSC3	1825	TRUE	0.59(0.44-0.81)	0.001	0.01	0

cg08007665	KCNQ1	3784	TRUE	1.7(1.23-2.34)	0.001	0.01	0
cg22415472	SLC5A7	60482	FALSE	1.69(1.23-2.32)	0.001	0.01	0
cg10090585	COVA1	10495	TRUE	1.72(1.23-2.39)	0.001	0.01	0
cg01369981	CAMKK2	10645	TRUE	0.56(0.39-0.79)	0.001	0.01	0
cg00892393	MGC35212	254528	FALSE	1.79(1.26-2.52)	0.001	0.01	0
cg21731286	TAS1R2	80834	FALSE	0.58(0.42-0.8)	0.001	0.01	0
cg22794078	C15orf44	81556	TRUE	0.59(0.42-0.81)	0.001	0.01	0
cg17729941	S100A13	6284	TRUE	1.7(1.24-2.34)	0.001	0.01	0
cg21621248	LRRTM1	347730	TRUE	0.59(0.43-0.81)	0.001	0.01	0
cg25021247	AMT	275	FALSE	0.61(0.45-0.82)	0.001	0.01	0
cg21432842	CSF3	1440	FALSE	0.59(0.43-0.81)	0.001	0.01	0
cg08624249	KIAA0889	25781	FALSE	1.77(1.26-2.5)	0.001	0.01	0
cg12936747	RBP4	5950	TRUE	0.58(0.42-0.81)	0.001	0.01	0
cg05261824	ZNF165	7718	FALSE	0.61(0.45-0.82)	0.001	0.01	0
cg11884546	ITGAX	3687	FALSE	0.58(0.42-0.81)	0.001	0.01	0
cg18055007	DDAH2	23564	TRUE	0.59(0.43-0.82)	0.001	0.01	0
cg10779183	ELA3A	10136	FALSE	1.76(1.25-2.48)	0.001	0.01	0
cg11933267	PLA2G2F	64600	FALSE	1.67(1.23-2.27)	0.001	0.01	0
cg10414058	HDAC3	8841	FALSE	0.56(0.4-0.8)	0.001	0.01	0
cg05606799	KISS1	3814	FALSE	0.59(0.44-0.81)	0.001	0.01	0
cg08261094	SFRP4	6424	TRUE	0.57(0.4-0.8)	0.001	0.01	0
cg15262516	COL4A2	1284	FALSE	1.72(1.24-2.4)	0.001	0.01	0
cg06131936	OSBPL5	114879	FALSE	1.69(1.24-2.32)	0.001	0.01	0
cg18354594	FLJ45717	388759	FALSE	0.58(0.42-0.8)	0.001	0.01	0
cg02575859	RPS4X	6191	TRUE	1.81(1.27-2.57)	0.001	0.01	0
cg07942995	ZSCAN1	284312	TRUE	0.55(0.39-0.79)	0.001	0.01	0
cg20516209	EMILIN1	11117	FALSE	0.57(0.41-0.8)	0.001	0.01	0

cg02564523	C7orf19	80228	TRUE	1.61(1.2-2.16)	0.001	0.01	0
cg15836722	IL1B	3553	FALSE	0.59(0.43-0.82)	0.001	0.01	−1
cg24471894	KIAA0020	9933	FALSE	0.48(0.31-0.76)	0.001	0.01	0
cg27181079	FLJ10292	55110	TRUE	1.88(1.29-2.74)	0.001	0.01	0
cg15873633	ZNF37A	7587	TRUE	1.72(1.24-2.38)	0.001	0.01	0
cg17328659	STUB1	10273	TRUE	0.58(0.42-0.81)	0.001	0.01	0
cg11164347	TRAPPC3	27095	TRUE	1.71(1.23-2.38)	0.001	0.01	0
cg17651255	SYF2	25949	TRUE	1.8(1.26-2.57)	0.001	0.01	0
cg11700584	RPL36AL	6166	TRUE	1.69(1.23-2.33)	0.001	0.01	0
cg10964421	TNFRSF10D	8793	TRUE	1.75(1.24-2.47)	0.001	0.01	0
cg15774283	PTGS2	5743	TRUE	0.58(0.42-0.8)	0.001	0.01	0
cg23889021	CTDSP1	58190	TRUE	1.76(1.25-2.5)	0.001	0.01	0
cg11970458	PYCARD	29108	TRUE	1.75(1.25-2.45)	0.001	0.01	0
cg16225429	TUSC2	11334	TRUE	1.88(1.28-2.76)	0.001	0.01	0
cg17699374	MGC35206	339669	FALSE	0.56(0.39-0.79)	0.001	0.01	0
cg08747889	PTK7	5754	TRUE	0.6(0.44-0.82)	0.001	0.01	0
cg01106788	KIAA1632	57724	FALSE	0.57(0.41-0.81)	0.001	0.01	0
cg03686067	C18orf14	79839	FALSE	1.68(1.23-2.31)	0.001	0.01	0
cg01607495	BAG4	9530	TRUE	0.58(0.42-0.81)	0.001	0.01	0
cg17421623	C3orf9	56983	FALSE	0.61(0.45-0.83)	0.001	0.01	−1
cg03533058	NR4A1	3164	FALSE	1.69(1.22-2.33)	0.001	0.01	0
cg07141002	H1F0	3005	TRUE	0.62(0.46-0.83)	0.001	0.01	0
cg19729279	PHF15	23338	TRUE	1.66(1.22-2.27)	0.001	0.01	0
cg25372103	DLL1	28514	TRUE	1.78(1.26-2.51)	0.001	0.01	0
cg20125091	GFI1	2672	TRUE	0.6(0.43-0.82)	0.001	0.01	0
cg09299388	PGK2	5232	FALSE	1.72(1.24-2.39)	0.001	0.01	0
cg04042106	MUS81	80198	TRUE	0.58(0.42-0.81)	0.001	0.01	−1

cg00935364	PKP3	11187	TRUE	1.67(1.22-2.28)	0.001	0.01	0
cg09874776	WFDC13	164237	FALSE	1.79(1.25-2.55)	0.001	0.01	0
cg19037167	TLR2	7097	TRUE	0.58(0.42-0.81)	0.001	0.01	0
cg24794531	TRPC1	7220	TRUE	0.58(0.42-0.81)	0.001	0.01	0
cg25040783	APPL	26060	TRUE	0.59(0.43-0.82)	0.001	0.01	0
cg00729275	C18orf16	147429	FALSE	1.65(1.22-2.23)	0.001	0.01	0
cg26014796	RNF39	80352	TRUE	0.59(0.43-0.82)	0.001	0.01	0
cg16746462	LNPEP	4012	TRUE	1.65(1.21-2.26)	0.001	0.01	0
cg17964955	RBP3	5949	FALSE	1.66(1.23-2.25)	0.001	0.01	0
cg26813908	CCDC55	84081	TRUE	1.7(1.22-2.35)	0.001	0.01	0
cg15870225	RNF41	10193	TRUE	0.58(0.42-0.81)	0.001	0.01	0
cg03604278	CIDEC	63924	FALSE	1.81(1.26-2.59)	0.001	0.01	0
cg12438034	MAGEF1	64110	TRUE	0.59(0.43-0.82)	0.001	0.01	0
cg20851245	RNF25	64320	FALSE	0.59(0.43-0.82)	0.001	0.01	0
cg12400041	DST	667	TRUE	0.6(0.43-0.82)	0.001	0.01	0
cg14400118	MMP2	4313	TRUE	0.59(0.43-0.82)	0.001	0.01	0
cg02831604	ISL2	64843	TRUE	0.57(0.4-0.81)	0.001	0.01	0
cg16792800	GPC6	10082	TRUE	0.59(0.43-0.82)	0.001	0.01	0
cg13168820	PTPRT	11122	TRUE	0.58(0.42-0.8)	0.001	0.01	0
cg09002774	SLC35B1	10237	TRUE	1.86(1.28-2.71)	0.001	0.01	0
cg12970008	OSBPL5	114879	TRUE	0.6(0.44-0.82)	0.001	0.01	0
cg02549424	AIFL	150209	TRUE	1.75(1.24-2.45)	0.001	0.01	0
cg24641737	DENND2D	79961	FALSE	0.58(0.42-0.81)	0.001	0.01	0
cg18448480	FLJ35785	283796	FALSE	0.61(0.45-0.82)	0.001	0.01	0
cg27400772	DPF1	8193	TRUE	1.71(1.23-2.37)	0.001	0.01	0
cg12832649	SPOCK	6695	TRUE	0.56(0.39-0.79)	0.001	0.01	0
cg03699566	FOLR1	2348	FALSE	1.79(1.25-2.57)	0.001	0.01	0

cg02226939	BLMH	642	TRUE	0.59(0.42-0.81)	0.001	0.01	0
cg19801560	HIST1H4G	8369	TRUE	0.44(0.27-0.73)	0.001	0.01	0
cg26531804	SPINT1	6692	TRUE	0.6(0.44-0.82)	0.001	0.01	0
cg08843314	CXCR3	2833	FALSE	1.88(1.28-2.76)	0.001	0.01	0
cg21818252	SEC24B	10427	TRUE	0.59(0.42-0.82)	0.001	0.01	0
cg16218424	CMAS	55907	TRUE	1.7(1.22-2.36)	0.001	0.01	0
cg04488521	ZNF354C	30832	TRUE	0.52(0.35-0.77)	0.001	0.01	0
cg00319692	ATP6V0D2	245972	FALSE	0.58(0.42-0.81)	0.001	0.01	0
cg05949660	MICAL1	64780	TRUE	0.61(0.45-0.83)	0.001	0.01	−1
cg20001829	TMEM25	84866	TRUE	0.54(0.38-0.79)	0.001	0.01	0
cg13313036	SLC29A2	3177	TRUE	1.67(1.22-2.29)	0.001	0.01	0
cg22161874	TRPM6	140803	FALSE	0.61(0.46-0.83)	0.001	0.02	0
cg00563926	TGFBR3	7049	TRUE	0.6(0.44-0.82)	0.001	0.02	0
cg10978355	CKMT2	1160	TRUE	0.59(0.42-0.82)	0.001	0.02	0
cg15431576	C10orf83	118812	TRUE	1.76(1.24-2.5)	0.001	0.02	0
cg25722142	CITED2	10370	TRUE	1.72(1.23-2.4)	0.001	0.02	0
cg21035142	CRYGN	155051	TRUE	1.68(1.22-2.3)	0.001	0.02	0
cg04631202	MAN1C1	57134	TRUE	0.6(0.44-0.83)	0.001	0.02	−1
cg18272264	CACNB4	785	TRUE	0.61(0.45-0.83)	0.001	0.02	0
cg26741595	HIST1H2BD	3017	TRUE	1.73(1.23-2.43)	0.001	0.02	0
cg03973663	LYN	4067	TRUE	0.59(0.43-0.82)	0.001	0.02	0
cg18711066	NFATC3	4775	TRUE	1.67(1.21-2.29)	0.001	0.02	0
cg26320696	PARVA	55742	TRUE	0.55(0.38-0.79)	0.001	0.02	0
cg19282714	PADI2	11240	TRUE	0.59(0.43-0.81)	0.001	0.02	0
cg14407437	FABP3	2170	TRUE	0.58(0.42-0.81)	0.001	0.02	0
cg04809787	CHRNB1	1140	TRUE	0.61(0.45-0.83)	0.001	0.02	0
cg09584711	HPR	3250	FALSE	0.61(0.45-0.83)	0.001	0.02	0

cg03611555	SLC25A21	89874	TRUE	0.57(0.41-0.8)	0.001	0.02	0
cg03301801	FNDC7	163479	FALSE	0.57(0.4-0.81)	0.001	0.02	0
cg19034028	CDH3	1001	TRUE	0.63(0.47-0.83)	0.001	0.02	0
cg12682367	FLJ46358	400110	FALSE	1.65(1.21-2.25)	0.001	0.02	0
cg04748704	CHRM2	1129	FALSE	1.73(1.23-2.44)	0.001	0.02	0
cg27161973	DDX27	55661	TRUE	1.7(1.23-2.36)	0.001	0.02	0
cg09039163	LOC339768	339768	TRUE	0.63(0.47-0.83)	0.001	0.02	0
cg16504798	MYO1F	4542	FALSE	0.61(0.45-0.83)	0.001	0.02	0
cg16155702	FGF21	26291	FALSE	1.7(1.22-2.36)	0.001	0.02	0
cg07607462	UBR1	197131	TRUE	1.74(1.24-2.45)	0.001	0.02	0
cg24147596	ARL14	80117	FALSE	1.67(1.21-2.29)	0.001	0.02	0
cg05607401	GMFG	9535	FALSE	1.63(1.2-2.21)	0.001	0.02	0
cg17745122	IRX3	79191	TRUE	0.58(0.42-0.8)	0.001	0.02	0
cg00629585	GFI1B	8328	FALSE	1.71(1.22-2.39)	0.001	0.02	0
cg26159905	ASB10	136371	FALSE	1.67(1.21-2.3)	0.001	0.02	0
cg14153740	TRY1	136541	FALSE	1.63(1.2-2.21)	0.001	0.02	0
cg18473117	CCDC22	28952	TRUE	0.48(0.31-0.75)	0.001	0.02	0
cg26129916	GGA3	23163	TRUE	1.65(1.21-2.24)	0.001	0.02	0
cg03030757	FANCE	2178	TRUE	0.61(0.45-0.83)	0.001	0.02	0
cg18705776	CALML3	810	FALSE	1.66(1.21-2.28)	0.001	0.02	0
cg15703690	IL6	3569	FALSE	0.58(0.41-0.81)	0.001	0.02	0
cg18117847	ZNFN1A1	10320	FALSE	1.83(1.26-2.65)	0.001	0.02	0
cg01348086	RGS14	10636	FALSE	1.68(1.22-2.31)	0.001	0.02	0
cg04488758	USP44	84101	FALSE	1.63(1.2-2.21)	0.001	0.02	0
cg02192855	HIST1H2BI	8346	TRUE	1.7(1.22-2.37)	0.001	0.02	0
cg03085312	RARA	5914	FALSE	0.61(0.45-0.83)	0.001	0.02	0
cg25495394	SAP18	10284	TRUE	1.64(1.21-2.23)	0.002	0.02	0

cg21123573	SIRT4	23409	TRUE	1.75(1.23-2.49)	0.002	0.02	0
cg16626670	CLEC4G	339390	FALSE	0.61(0.45-0.83)	0.002	0.02	0
cg04459030	GHRH	2691	FALSE	1.63(1.2-2.23)	0.002	0.02	0
cg06746171	ATOX1	475	TRUE	1.72(1.23-2.4)	0.002	0.02	0
cg22375610	APOBEC2	10930	FALSE	1.63(1.2-2.21)	0.002	0.02	0
cg26124016	RARB	5915	TRUE	0.6(0.44-0.82)	0.002	0.02	0
cg18085435	ATP8B1	5205	TRUE	1.7(1.22-2.36)	0.002	0.02	0
cg23696834	GNMT	27232	TRUE	0.57(0.4-0.81)	0.002	0.02	0
cg24751129	GNMT	27232	TRUE	0.58(0.41-0.81)	0.002	0.02	0
cg27236973	KRT17	3872	TRUE	0.58(0.42-0.81)	0.002	0.02	0
cg16272420	PNLIPRP2	5408	FALSE	1.75(1.24-2.47)	0.002	0.02	0
cg16016036	TPO	7173	TRUE	0.54(0.37-0.79)	0.002	0.02	0
cg04915566	RUNX1	861	FALSE	1.67(1.21-2.3)	0.002	0.02	0
cg25775449	LTB4R	1241	FALSE	0.59(0.43-0.82)	0.002	0.02	0
cg03875678	GZMB	3002	FALSE	1.62(1.19-2.19)	0.002	0.02	0
cg05840553	LRSAM1	90678	FALSE	0.61(0.45-0.83)	0.002	0.02	0
cg19770955	DUS4L	11062	FALSE	0.59(0.43-0.82)	0.002	0.02	0
cg16581199	TSSK1	83942	FALSE	1.68(1.21-2.32)	0.002	0.02	0
cg09426307	SEC14L3	266629	FALSE	1.72(1.23-2.42)	0.002	0.02	0
cg05253327	B3GNT1	10678	FALSE	0.6(0.43-0.83)	0.002	0.02	0
cg25250998	GSTO2	119391	TRUE	1.63(1.21-2.21)	0.002	0.02	0
cg00394658	PTPRJ	5795	TRUE	0.63(0.47-0.84)	0.002	0.02	0
cg22386774	CRYGA	1418	FALSE	1.61(1.2-2.16)	0.002	0.02	0
cg05091653	SP100	6672	FALSE	1.75(1.23-2.47)	0.002	0.02	0
cg00935388	RNASE8	122665	FALSE	0.62(0.46-0.84)	0.002	0.02	0
cg12515638	SFRP4	6424	TRUE	0.62(0.46-0.84)	0.002	0.02	0
cg07448499	CTSH	1512	TRUE	1.7(1.23-2.34)	0.002	0.02	0

cg02883161	MSI1	4440	TRUE	1.89(1.26-2.82)	0.002	0.02	0
cg27221338	KDELR2	11014	TRUE	0.6(0.43-0.83)	0.002	0.02	0
cg10591174	MYL2	4633	FALSE	1.67(1.21-2.31)	0.002	0.02	0
cg00406844	RFPL3	10738	FALSE	1.67(1.22-2.28)	0.002	0.02	0
cg03165700	ATM	472	TRUE	1.66(1.21-2.28)	0.002	0.02	0
cg27601582	ITGB1BP3	27231	FALSE	0.57(0.4-0.8)	0.002	0.02	0
cg25741452	KITLG	4254	TRUE	0.38(0.21-0.69)	0.002	0.02	0
cg10163825	C16orf25	124093	TRUE	0.62(0.46-0.83)	0.002	0.02	0
cg06621126	HSF4	3299	TRUE	0.59(0.43-0.81)	0.002	0.02	0
cg20832020	VSIG9	201633	FALSE	1.69(1.22-2.36)	0.002	0.02	0
cg25662535	ART5	116969	TRUE	0.62(0.46-0.83)	0.002	0.02	0
cg11170179	KLK10	5655	TRUE	0.59(0.42-0.82)	0.002	0.02	0
cg14076161	PRB4	5545	FALSE	1.65(1.2-2.26)	0.002	0.02	0
cg03900542	DEDD2	162989	TRUE	1.74(1.23-2.46)	0.002	0.02	0
cg01410472	CRISPLD1	83690	TRUE	0.61(0.45-0.83)	0.002	0.02	0
cg14261863	ELMO2	63916	TRUE	1.79(1.25-2.56)	0.002	0.02	0
cg22424746	VTCN1	79679	FALSE	1.64(1.2-2.23)	0.002	0.02	0
cg18905952	NRBP1	29959	TRUE	1.78(1.25-2.55)	0.002	0.02	0
cg10798171	ICA1	3382	TRUE	0.61(0.44-0.83)	0.002	0.02	0
cg08905118	RP2	6102	TRUE	1.99(1.3-3.06)	0.002	0.02	0
cg01656216	ZNF438	220929	FALSE	0.59(0.42-0.82)	0.002	0.02	0
cg02245418	ZNF364	27246	FALSE	0.58(0.41-0.82)	0.002	0.02	0
cg18843688	NUFIP1	26747	TRUE	1.65(1.21-2.26)	0.002	0.02	0
cg15384717	PRPF3	9129	TRUE	1.68(1.21-2.33)	0.002	0.02	0
cg25551168	AVP	551	FALSE	0.63(0.47-0.84)	0.002	0.02	0
cg06808983	G6PC3	92579	TRUE	1.63(1.2-2.22)	0.002	0.02	0
cg23412850	SOCS2	8835	TRUE	0.59(0.42-0.82)	0.002	0.02	0

cg13492227	FGF11	2256	TRUE	0.59(0.43-0.82)	0.002	0.02	0
cg18053607	PIB5PA	27124	FALSE	0.63(0.46-0.84)	0.002	0.02	−1
cg21197871	C8orf38	137682	TRUE	0.6(0.43-0.82)	0.002	0.02	0
cg21291985	BTBD4	140685	TRUE	0.62(0.46-0.83)	0.002	0.02	0
cg03297731	GDPD3	79153	FALSE	1.64(1.2-2.25)	0.002	0.02	0
cg19208681	RAD51C	5889	TRUE	1.61(1.2-2.15)	0.002	0.02	0
cg11255230	BCL2	596	TRUE	1.76(1.23-2.51)	0.002	0.02	0
cg08548888	MYD88	4615	TRUE	1.8(1.26-2.56)	0.002	0.02	0
cg23097681	ARHGAP24	83478	TRUE	0.59(0.43-0.81)	0.002	0.02	0
cg18780284	SPRR1B	6699	FALSE	1.64(1.21-2.23)	0.002	0.02	0
cg07543883	ANGPTL6	83854	FALSE	1.64(1.2-2.23)	0.002	0.02	0
cg21601405	CD40	958	TRUE	0.58(0.41-0.82)	0.002	0.02	0
cg10421192	CTAGE1	64693	TRUE	0.5(0.33-0.77)	0.002	0.02	0
cg11608424	LOC253012	253012	FALSE	1.8(1.25-2.59)	0.002	0.02	0
cg15422147	SERPINB5	5268	FALSE	1.65(1.2-2.26)	0.002	0.02	0
cg13288195	FBXL22	283807	FALSE	0.6(0.44-0.83)	0.002	0.02	0
cg22441882	SLC18A1	6570	FALSE	1.8(1.26-2.59)	0.002	0.02	0
cg01136458	CSMD1	64478	FALSE	1.66(1.21-2.28)	0.002	0.02	0
cg04929865	BGN	633	FALSE	1.61(1.19-2.17)	0.002	0.02	0
cg01497576	SLC24A5	283652	FALSE	1.62(1.21-2.18)	0.002	0.02	0
cg20259398	CDCA7L	55536	TRUE	1.78(1.25-2.53)	0.002	0.02	0
cg03862760	MGC10911	84262	TRUE	1.73(1.23-2.43)	0.002	0.02	0
cg09146695	LEF1	51176	TRUE	1.66(1.21-2.28)	0.002	0.02	0
cg25635500	RRP22	10633	TRUE	0.61(0.45-0.84)	0.002	0.02	0
cg05323436	LAMB2	3913	TRUE	0.62(0.45-0.84)	0.002	0.02	0
cg20430816	GENX-3414	8987	TRUE	0.6(0.43-0.82)	0.002	0.02	0
cg00571634	WDR5B	54554	TRUE	1.78(1.24-2.57)	0.002	0.02	0

cg20492401	KIAA0195	9772	TRUE	1.63(1.21-2.19)	0.002	0.02	0
cg06207460	AK3	50808	TRUE	1.8(1.24-2.61)	0.002	0.02	0
cg16396488	PLA2G1B	5319	FALSE	1.62(1.19-2.19)	0.002	0.02	0
cg15156367	ABHD7	253152	TRUE	0.58(0.41-0.81)	0.002	0.02	0
cg09425228	CCL20	6364	FALSE	1.62(1.2-2.19)	0.002	0.02	0
cg11560645		3502	FALSE	1.69(1.21-2.35)	0.002	0.02	0
cg01726767	LALBA	3906	FALSE	1.68(1.22-2.3)	0.002	0.02	0
cg10895130	BNIPL	149428	FALSE	0.62(0.45-0.84)	0.002	0.02	0
cg12891678	SPRR2D	6703	FALSE	1.62(1.19-2.2)	0.002	0.02	0
cg22010317	CXorf36	79742	FALSE	1.61(1.19-2.17)	0.002	0.02	0
cg11119596	CD96	10225	FALSE	1.81(1.24-2.64)	0.002	0.02	0
cg20308817	CLEC11A	6320	FALSE	0.6(0.43-0.83)	0.002	0.02	0
cg19822214	CNOT3	4849	TRUE	1.76(1.25-2.49)	0.002	0.02	0
cg03660451	RECQL5	9400	FALSE	0.61(0.44-0.84)	0.002	0.02	0
cg22821324	GPM6A	2823	TRUE	0.58(0.41-0.81)	0.002	0.02	0
cg13108328	PLEKHA5	54477	TRUE	1.65(1.2-2.26)	0.002	0.02	0
cg21716693	CPNE5	57699	TRUE	0.59(0.43-0.82)	0.002	0.02	0
cg16052901	VPS52	6293	FALSE	1.67(1.21-2.31)	0.002	0.02	0
cg08440425	LRRC51	220074	TRUE	1.62(1.2-2.19)	0.002	0.02	0
cg14473145	CLEC14A	161198	TRUE	0.58(0.42-0.82)	0.002	0.02	0
cg02318426	NFKBIA	4792	TRUE	1.71(1.22-2.41)	0.002	0.02	0
cg08758850	NR6A1	2649	TRUE	0.56(0.39-0.8)	0.002	0.02	0
cg04764624	RAB11FIP4	84440	TRUE	0.61(0.44-0.83)	0.002	0.02	0
cg26024531	CHAF1B	8208	TRUE	1.68(1.22-2.3)	0.002	0.02	0
cg24585690	IL9	3578	FALSE	1.66(1.2-2.28)	0.002	0.02	0
cg14859417	PTPRE	5791	TRUE	0.61(0.45-0.84)	0.002	0.02	0
cg20385229	ALKBH	8846	FALSE	1.65(1.21-2.27)	0.002	0.02	0

cg14984307	ACYP1	97	TRUE	1.66(1.21-2.29)	0.002	0.02	0
cg02409351	CART1	8092	TRUE	0.59(0.42-0.82)	0.002	0.02	0
cg22421766	SCNN1D	6339	FALSE	0.61(0.45-0.84)	0.002	0.02	0
cg12563178	PLXDC2	84898	TRUE	0.55(0.38-0.79)	0.002	0.02	0
cg03712843	STOML3	161003	FALSE	0.61(0.45-0.83)	0.002	0.02	0
cg04415689	TMEM16G	50636	FALSE	1.85(1.25-2.75)	0.002	0.02	0
cg05641882	CXCL3	2921	TRUE	0.6(0.44-0.83)	0.002	0.02	0
cg13927251	FLT4	2324	TRUE	1.67(1.21-2.31)	0.002	0.02	0
cg19091641	ZP2	7783	FALSE	0.6(0.43-0.83)	0.002	0.02	0
cg22667787	APTX	54840	TRUE	1.63(1.2-2.23)	0.002	0.02	0
cg27299588	PTPRS	5802	FALSE	1.66(1.2-2.3)	0.002	0.02	0
cg16363586	BST2	684	TRUE	0.61(0.44-0.84)	0.002	0.02	−1
cg04280397	PLA2G6	8398	TRUE	0.61(0.45-0.84)	0.002	0.02	0
cg10770524	SMA3	10571	FALSE	1.6(1.19-2.15)	0.002	0.02	0
cg24480859	SLC2A5	6518	FALSE	0.6(0.44-0.83)	0.002	0.02	0
cg03025569	TRIM16	10626	FALSE	0.63(0.47-0.84)	0.002	0.02	0
cg06742978	ACCN2	41	TRUE	0.59(0.43-0.83)	0.002	0.02	0
cg03922337	RRAS2	22800	TRUE	1.65(1.2-2.28)	0.002	0.02	0
cg13892015	PPIL5	122769	TRUE	1.68(1.21-2.34)	0.002	0.02	0
cg10056627	GNMT	27232	TRUE	0.62(0.45-0.84)	0.002	0.02	0
cg20150565	ZNFX1	57169	TRUE	1.69(1.21-2.36)	0.002	0.02	0
cg07985359	EVI5L	115704	TRUE	1.59(1.19-2.13)	0.002	0.02	0
cg21624359	FFAR3	2865	FALSE	0.63(0.47-0.84)	0.002	0.02	0
cg03876618	IGFBP7	3490	TRUE	0.36(0.19-0.69)	0.002	0.02	0
cg02813863	APOLD1	81575	FALSE	0.58(0.41-0.81)	0.002	0.02	0
cg15640375	PRG2	79948	TRUE	0.57(0.4-0.81)	0.002	0.02	0
cg25361106	TLX2	3196	TRUE	0.6(0.43-0.83)	0.002	0.02	0

cg01471384	DKK2	27123	TRUE	0.58(0.41-0.82)	0.002	0.02	0
cg09169633	MAN2A2	4122	FALSE	0.59(0.42-0.83)	0.002	0.02	0
cg00795268	C11orf55	399879	FALSE	0.59(0.43-0.83)	0.002	0.02	0
cg08040428	PSD3	23362	TRUE	0.61(0.45-0.83)	0.002	0.02	0
cg15616083	KCNQ2	3785	FALSE	0.63(0.47-0.85)	0.002	0.02	0
cg00105253	NUDT14	256281	TRUE	0.59(0.42-0.83)	0.002	0.02	−1
cg20644981	RPS3A	6189	TRUE	1.9(1.28-2.81)	0.002	0.02	0
cg02493771	KRTAP13-2	337959	FALSE	1.62(1.19-2.2)	0.002	0.02	0
cg09022993	SPATA18	132671	TRUE	0.57(0.39-0.82)	0.002	0.02	0
cg18669588	PTK9L	11344	TRUE	1.63(1.19-2.23)	0.002	0.02	0
cg12297221	ART5	116969	TRUE	0.6(0.44-0.83)	0.002	0.02	0
cg03003745	UNQ473	284340	FALSE	1.62(1.19-2.21)	0.002	0.02	0
cg06630567	AMBP	259	FALSE	0.62(0.46-0.84)	0.002	0.02	0
cg07243932	SLC30A4	7782	TRUE	1.65(1.2-2.28)	0.002	0.02	0
cg11849692	LDB1	8861	FALSE	0.61(0.45-0.83)	0.002	0.02	0
cg03459809	EPHX1	2052	FALSE	1.71(1.22-2.41)	0.002	0.02	0
cg19854301	CDH13	1012	TRUE	0.61(0.44-0.83)	0.002	0.02	0
cg18621299	EVL	51466	FALSE	1.72(1.21-2.43)	0.002	0.02	0
cg10316635	INADL	10207	TRUE	0.62(0.45-0.84)	0.002	0.02	0
cg22527415	LSM16	80153	TRUE	1.63(1.19-2.23)	0.002	0.02	0
cg04880762	VAMP1	6843	TRUE	1.67(1.2-2.31)	0.002	0.02	0
cg11769360	LRAP	64167	FALSE	0.59(0.43-0.83)	0.002	0.02	0
cg01275830	C10orf95	79946	TRUE	0.6(0.44-0.83)	0.002	0.02	0
cg11220060	KLF1	10661	FALSE	0.63(0.46-0.85)	0.002	0.02	0
cg18801806	DLNB14	338657	TRUE	1.61(1.2-2.17)	0.002	0.02	0
cg22349489	HIST1H2AH	85235	TRUE	1.66(1.2-2.3)	0.002	0.02	0
cg23178308	C21orf124	85006	TRUE	0.59(0.42-0.83)	0.002	0.02	0

cg00602891	C17orf62	79415	TRUE	1.69(1.22-2.35)	0.002	0.02	0
cg27412902	IL29	282618	FALSE	1.74(1.21-2.49)	0.002	0.02	0
cg02317907	DIRAS3	9077	TRUE	0.59(0.43-0.82)	0.002	0.02	0
cg06154597	MGC4618	84286	FALSE	1.63(1.19-2.23)	0.002	0.02	0
cg11466837	TRIM29	23650	FALSE	1.7(1.21-2.4)	0.002	0.02	0
cg14319409	GLRA1	2741	TRUE	1.64(1.19-2.26)	0.002	0.02	1
cg04833845	KCNN4	3783	FALSE	1.66(1.2-2.3)	0.002	0.02	0
cg26608667	MGC11257	84310	TRUE	0.63(0.47-0.85)	0.002	0.02	0
cg01615704	MALL	7851	TRUE	0.59(0.43-0.82)	0.002	0.02	0
cg23054883	FZD10	11211	TRUE	0.59(0.43-0.83)	0.002	0.02	0
cg05237543	MTDH	92140	TRUE	1.68(1.2-2.34)	0.002	0.02	0
cg04548378	CDKN1C	1028	TRUE	1.76(1.22-2.52)	0.002	0.02	0
cg22946150	SH3GL3	6457	TRUE	0.5(0.32-0.78)	0.002	0.02	0
cg01610488	TRPA1	8989	TRUE	0.59(0.42-0.82)	0.002	0.02	0
cg26718122	SFRS6	6431	TRUE	1.94(1.28-2.95)	0.002	0.02	0
cg17240987	WFDC3	140686	FALSE	0.6(0.44-0.83)	0.002	0.02	0
cg08064891	PTOV1	53635	TRUE	1.64(1.19-2.26)	0.002	0.02	0
cg23226134	CLCN6	1185	TRUE	1.62(1.18-2.21)	0.002	0.02	0
cg18468842	SLC13A3	64849	TRUE	0.63(0.46-0.85)	0.002	0.02	0
cg13210534	HSPB2	3316	FALSE	1.61(1.19-2.17)	0.002	0.02	0
cg05894797	PSEN2	5664	TRUE	0.6(0.43-0.84)	0.002	0.02	0
cg08569678	LY6K	54742	TRUE	0.63(0.47-0.85)	0.002	0.02	0
cg21449655	CASP9	842	TRUE	1.65(1.19-2.29)	0.002	0.02	0
cg09513026	WNT8A	7478	FALSE	0.63(0.46-0.84)	0.002	0.02	0
cg19042947	SERPINA4	5267	FALSE	1.62(1.19-2.2)	0.002	0.02	0
cg26829529	SPACA3	124912	FALSE	1.6(1.18-2.17)	0.002	0.02	0
cg05675373	KCNC4	3749	TRUE	1.76(1.22-2.55)	0.002	0.02	1

cg01739167	CHRNE	1145	FALSE	1.69(1.21-2.35)	0.002	0.02	0
cg23346960	ZFP36	7538	TRUE	1.65(1.19-2.29)	0.002	0.02	0
cg16899442	C16orf25	124093	FALSE	0.63(0.47-0.85)	0.002	0.02	0
cg05508084	ZNF667	63934	TRUE	0.57(0.39-0.82)	0.002	0.02	0
cg07356771	CHDH	55349	TRUE	0.6(0.44-0.83)	0.002	0.02	0
cg10365880	PKD2L1	9033	FALSE	1.65(1.2-2.27)	0.002	0.02	0
cg10627136	HINT1	3094	TRUE	1.68(1.2-2.35)	0.002	0.02	0
cg08017606	SH3RF2	153769	TRUE	1.67(1.2-2.34)	0.002	0.02	0
cg09874127	UBE1L	7318	FALSE	1.6(1.18-2.17)	0.002	0.02	0
cg15989091	LOXL3	84695	FALSE	0.63(0.47-0.84)	0.002	0.02	0
cg25524473	DUSP5	1847	TRUE	1.67(1.2-2.32)	0.002	0.02	0
cg16682903	ACVR1	90	FALSE	0.58(0.41-0.83)	0.002	0.02	−1
cg07685034	PCBP3	54039	FALSE	1.63(1.19-2.24)	0.002	0.02	0
cg02144933	AOX1	316	TRUE	0.59(0.42-0.83)	0.002	0.02	0
cg14611112	LCN6	158062	FALSE	0.61(0.45-0.84)	0.002	0.02	0
cg08222662	ZC3HAV1	56829	TRUE	1.72(1.22-2.44)	0.002	0.02	0
cg17749384	MPP7	143098	FALSE	0.62(0.46-0.84)	0.002	0.02	0
cg23617121	OSBPL5	114879	TRUE	1.68(1.21-2.33)	0.002	0.02	0
cg16293991	CPSF3L	54973	TRUE	1.69(1.2-2.37)	0.002	0.02	0
cg22580512	NCOR2	9612	TRUE	0.61(0.44-0.84)	0.002	0.02	−1
cg10683939	C16orf47	388289	FALSE	0.59(0.42-0.83)	0.002	0.02	0
cg09082287	DNAJC6	9829	TRUE	0.61(0.44-0.83)	0.002	0.02	0
cg21667943	CIP29	84324	TRUE	1.64(1.19-2.28)	0.002	0.02	0
cg03851112	BBS1	582	FALSE	0.6(0.43-0.84)	0.002	0.02	0
cg12435792	PDE6B	5158	TRUE	1.65(1.19-2.29)	0.002	0.02	0
cg26199493	LIN9	286826	TRUE	0.6(0.44-0.84)	0.002	0.02	0
cg09475757	NEIL3	55247	TRUE	1.66(1.19-2.31)	0.002	0.02	0

cg21572316	ABCA1	19	TRUE	0.61(0.45-0.84)	0.002	0.02	0
cg25856811	SPRR3	6707	FALSE	1.66(1.19-2.3)	0.002	0.02	0
cg06777581	RNF8	9025	TRUE	1.62(1.18-2.21)	0.002	0.02	0
cg26825412	SOX18	54345	FALSE	0.64(0.48-0.86)	0.002	0.02	0
cg15447486	GPR109B	8843	FALSE	0.63(0.46-0.85)	0.002	0.02	0
cg19237753	PTPNS1	140885	TRUE	0.61(0.44-0.84)	0.002	0.02	0
cg06589885	HFE2	148738	FALSE	1.63(1.19-2.24)	0.002	0.02	0
cg10938446	IL1RN	3557	FALSE	0.63(0.46-0.85)	0.002	0.02	0
cg03491478	MAPK8IP1	9479	TRUE	1.69(1.21-2.37)	0.002	0.02	0
cg04834572	DUSP13	51207	FALSE	0.56(0.39-0.82)	0.002	0.02	0
cg18189938	EPB41	2035	TRUE	1.64(1.19-2.28)	0.002	0.02	0
cg27532722	MMP11	4320	TRUE	0.61(0.44-0.84)	0.002	0.02	0
cg01344171	C6orf206	221421	TRUE	0.64(0.47-0.86)	0.002	0.02	0
cg05038121	FAH	2184	TRUE	1.68(1.2-2.35)	0.002	0.02	0
cg17877494	ACAA1	30	TRUE	1.63(1.19-2.23)	0.002	0.02	0
cg10704923	HIST1H1T	3010	TRUE	0.51(0.32-0.79)	0.002	0.02	0
cg05592398	LIMK1	3984	TRUE	0.61(0.44-0.84)	0.002	0.02	0
cg22499237	PDPK1	5170	TRUE	1.71(1.2-2.43)	0.002	0.02	0
cg11851098	MGC26816	164684	TRUE	0.62(0.45-0.85)	0.002	0.02	0
cg07674153	TSHR	7253	TRUE	1.59(1.18-2.16)	0.002	0.02	0
cg25741794	EFHD2	79180	TRUE	1.58(1.17-2.13)	0.002	0.02	0
cg24196046	EIF2C3	192669	TRUE	1.72(1.2-2.45)	0.002	0.02	0
cg14070162	PTRF	284119	TRUE	0.63(0.46-0.85)	0.002	0.02	−1
cg13314167	C9orf84	158401	TRUE	0.61(0.45-0.84)	0.002	0.02	0
cg25294646	OBFC1	79991	TRUE	1.66(1.2-2.32)	0.002	0.02	0
cg17805404	GPR162	27239	FALSE	0.61(0.44-0.84)	0.002	0.02	0
cg07925687	RABL3	285282	TRUE	1.6(1.18-2.17)	0.002	0.02	0

cg22518733	CCL3	6348	FALSE	0.6(0.43-0.84)	0.002	0.02	0
cg21468416	NEK6	10783	TRUE	1.58(1.18-2.11)	0.002	0.02	0
cg11011938	SEMA5A	9037	TRUE	0.61(0.44-0.83)	0.002	0.02	0
cg11243196	ADAMTS3	9508	TRUE	0.62(0.46-0.84)	0.002	0.02	0
cg11179184	C8orf76	84933	TRUE	0.61(0.45-0.84)	0.002	0.02	0
cg07099407	KLC4	89953	TRUE	0.61(0.44-0.84)	0.002	0.02	0
cg16254309	CNTNAP2	26047	TRUE	0.58(0.41-0.82)	0.002	0.02	0
cg16752583	TRPV6	55503	FALSE	1.66(1.2-2.3)	0.002	0.02	0
cg25363445	ALX4	60529	TRUE	1.63(1.18-2.24)	0.002	0.02	0
cg25892041	UGT8	7368	FALSE	0.62(0.46-0.85)	0.002	0.02	0
cg12371177	PLEKHG6	55200	TRUE	0.62(0.45-0.85)	0.002	0.02	0
cg23352695	EVI2A	2123	FALSE	1.6(1.18-2.17)	0.002	0.02	0
cg04653021	DIRC2	84925	TRUE	0.6(0.42-0.84)	0.002	0.02	0
cg01442481	RALY	22913	TRUE	1.57(1.17-2.12)	0.002	0.02	0
cg07973967	CD79B	974	TRUE	0.59(0.42-0.83)	0.002	0.02	0
cg10748867	C1orf33	51154	FALSE	0.62(0.45-0.85)	0.002	0.02	0
cg09619786	RPP25	54913	TRUE	0.62(0.46-0.85)	0.002	0.02	0
cg04582938	UAP1L1	91373	TRUE	0.63(0.46-0.85)	0.002	0.02	0
cg19586576	GJA7	10052	TRUE	1.69(1.2-2.37)	0.002	0.02	0
cg13255629	MEPE	56955	FALSE	1.75(1.21-2.53)	0.002	0.02	0
cg05306176	FVT1	2531	TRUE	1.68(1.21-2.34)	0.002	0.02	0
cg22747092	BTBD14A	138151	TRUE	0.61(0.44-0.84)	0.002	0.02	0
cg20029201	BCL9L	283149	TRUE	1.62(1.19-2.21)	0.002	0.02	0
cg16761041	C9orf10OS	158293	TRUE	1.65(1.19-2.29)	0.002	0.02	0
cg04629204	EXTL1	2134	FALSE	1.66(1.2-2.3)	0.002	0.02	0
cg21238818	GAL3ST3	89792	TRUE	0.61(0.45-0.84)	0.002	0.02	0
cg16358738	AGXT	189	FALSE	1.66(1.2-2.3)	0.002	0.02	0

cg10375110	CHRNA9	55584	FALSE	1.64(1.19-2.25)	0.002	0.02	0
cg14358743	GAS6	2621	TRUE	0.61(0.44-0.85)	0.002	0.02	0
cg21771250	FAM83F	113828	TRUE	0.62(0.45-0.85)	0.002	0.02	0
cg09419900	C10orf89	118672	FALSE	0.64(0.48-0.86)	0.003	0.02	0
cg00899641	SF3B1	23451	TRUE	1.58(1.18-2.11)	0.003	0.02	0
cg23587449	LRAT	9227	TRUE	0.57(0.39-0.82)	0.003	0.02	0
cg21509023	HBA2	3040	TRUE	0.61(0.44-0.84)	0.003	0.02	0
cg12818699	C6orf32	9750	FALSE	1.59(1.17-2.16)	0.003	0.02	0
cg24194775	NPR2	4882	TRUE	0.62(0.46-0.85)	0.003	0.02	0
cg26991946	SEMA4G	57715	FALSE	1.63(1.18-2.26)	0.003	0.02	0
cg17740399	IPF1	3651	TRUE	0.57(0.4-0.82)	0.003	0.02	0
cg23799313	PAQR7	164091	FALSE	0.63(0.46-0.86)	0.003	0.02	0
cg20016416	SPTBN2	6712	FALSE	1.62(1.19-2.21)	0.003	0.02	0
cg01644850	ZNF551	90233	TRUE	0.61(0.45-0.84)	0.003	0.02	0
cg06701500	YWHAQ	10971	TRUE	0.62(0.45-0.85)	0.003	0.02	0
cg20973210	C19orf35	374872	TRUE	0.6(0.43-0.84)	0.003	0.02	0
cg02519218	CHFR	55743	TRUE	1.63(1.18-2.26)	0.003	0.02	0
cg06351503	RDBP	7936	FALSE	1.61(1.19-2.2)	0.003	0.02	0
cg04879235	PPY	5539	FALSE	1.67(1.2-2.32)	0.003	0.02	0
cg12855851	PGC	5225	FALSE	1.65(1.19-2.29)	0.003	0.02	0
cg25283823	HIST2H2BE	8349	TRUE	1.95(1.27-3.01)	0.003	0.02	0
cg11062095	C5orf15	56951	TRUE	1.72(1.2-2.46)	0.003	0.02	0
cg23350580	TBC1D3C	414060	FALSE	1.64(1.19-2.27)	0.003	0.02	0
cg17483510	GNB4	59345	TRUE	0.59(0.42-0.83)	0.003	0.02	0
cg17036737	RBM8A	9939	TRUE	1.65(1.19-2.27)	0.003	0.02	0
cg12864853	TPBG	7162	FALSE	0.58(0.41-0.84)	0.003	0.02	0
cg11328149	LY6G6C	80740	FALSE	1.74(1.21-2.51)	0.003	0.02	0

cg19286604	MGC39545	403312	TRUE	0.55(0.37-0.81)	0.003	0.02	0
cg00145118	GNPDA1	10007	FALSE	0.62(0.46-0.85)	0.003	0.02	0
cg20469837	GALNT5	11227	FALSE	0.6(0.42-0.84)	0.003	0.02	0
cg19769182	PRRT2	112476	TRUE	0.63(0.46-0.85)	0.003	0.02	0
cg27009703	HOXA9	3205	TRUE	0.61(0.44-0.84)	0.003	0.02	0
cg03833774	ZCCHC5	203430	FALSE	0.64(0.48-0.86)	0.003	0.02	0
cg18972811	SLIT2	9353	TRUE	0.6(0.43-0.83)	0.003	0.02	0
cg18149207	RORC	6097	FALSE	1.64(1.18-2.27)	0.003	0.02	1
cg25177452	PRP2	134285	TRUE	0.6(0.43-0.83)	0.003	0.02	0
cg03682712	LOXL1	4016	TRUE	0.61(0.44-0.84)	0.003	0.02	0
cg08849126	GPRC5A	9052	TRUE	0.58(0.4-0.83)	0.003	0.02	0
cg12556134	TGIF2	60436	TRUE	0.6(0.43-0.84)	0.003	0.02	0
cg17192247	MAPRE3	22924	TRUE	0.62(0.45-0.85)	0.003	0.02	0
cg02976574	FNDC3B	64778	FALSE	0.61(0.44-0.85)	0.003	0.02	0
cg07733031	SGTB	54557	TRUE	0.58(0.41-0.83)	0.003	0.02	0
cg15249164	ATP5O	539	TRUE	1.69(1.2-2.38)	0.003	0.02	0
cg19884262	FLJ46831	399823	TRUE	0.63(0.47-0.85)	0.003	0.02	0
cg13306784	INPP5E	56623	TRUE	1.59(1.17-2.17)	0.003	0.02	0
cg09682183	UNC93A	54346	FALSE	0.62(0.46-0.85)	0.003	0.02	0
cg15344028	ICOS	29851	FALSE	1.58(1.17-2.14)	0.003	0.02	0
cg24801210	PCNP	57092	TRUE	1.9(1.24-2.9)	0.003	0.02	0
cg03782453	FLJ90575	257236	TRUE	0.62(0.46-0.85)	0.003	0.02	0
cg00687674	TMEM84	283673	FALSE	1.57(1.16-2.11)	0.003	0.02	0
cg24352530	ABCG2	9429	TRUE	0.59(0.42-0.82)	0.003	0.02	0
cg04454951	UNQ739	375567	TRUE	1.64(1.18-2.28)	0.003	0.02	0
cg20264731	POLB	5423	TRUE	1.59(1.17-2.15)	0.003	0.02	0
cg18635110	C20orf116	65992	TRUE	1.75(1.21-2.53)	0.003	0.02	0

cg05668853	RAB34	83871	TRUE	0.63(0.46-0.86)	0.003	0.02	0
cg22757447	IMPACT	55364	TRUE	0.6(0.44-0.83)	0.003	0.02	0
cg18091964	CXorf9	54440	FALSE	1.65(1.19-2.28)	0.003	0.02	0
cg08263647	LXN	56925	FALSE	0.61(0.44-0.84)	0.003	0.02	0
cg21404906	XRCC5	7520	TRUE	1.69(1.2-2.38)	0.003	0.02	0
cg03752885	DAPK3	1613	FALSE	0.61(0.44-0.85)	0.003	0.02	0
cg08860143	MYLPF	29895	FALSE	1.73(1.21-2.48)	0.003	0.02	0
cg27622610	OR1G1	8390	FALSE	1.7(1.2-2.43)	0.003	0.02	0
cg07845392	SLC25A10	1468	TRUE	0.64(0.48-0.86)	0.003	0.02	0
cg02955504	TXNL4A	10907	TRUE	0.64(0.47-0.86)	0.003	0.02	0
cg20424530	IRAK4	51135	TRUE	1.58(1.17-2.13)	0.003	0.02	0
cg11999384	GRN	2896	TRUE	1.6(1.17-2.19)	0.003	0.02	0
cg14519000	GATA5	140628	TRUE	1.68(1.19-2.35)	0.003	0.02	0
cg14450605	NCOA4	8031	TRUE	0.59(0.42-0.84)	0.003	0.02	0
cg17757055	DKFZP564O0823	25849	TRUE	0.61(0.44-0.85)	0.003	0.02	0
cg26363196	ST6GALNAC3	256435	TRUE	0.59(0.42-0.83)	0.003	0.02	0
cg01015871	MT4	84560	FALSE	1.64(1.18-2.26)	0.003	0.02	0
cg17166338	TERT	7015	TRUE	0.63(0.47-0.84)	0.003	0.02	0
cg25023829	H2BFS	54145	TRUE	0.58(0.41-0.83)	0.003	0.02	0
cg08141873	HRAS	3265	TRUE	1.61(1.18-2.2)	0.003	0.02	0
cg04454050	TREML1	340205	FALSE	0.64(0.47-0.86)	0.003	0.02	0
cg02194878	EPHA8	2046	TRUE	0.59(0.42-0.83)	0.003	0.02	0
cg08314660	PKP3	11187	TRUE	1.76(1.21-2.56)	0.003	0.02	0
cg21087043	DCX	1641	FALSE	1.68(1.19-2.37)	0.003	0.02	0
cg22843446	ASAM	79827	TRUE	0.62(0.46-0.85)	0.003	0.02	0
cg21172540	TSSK3	81629	TRUE	0.61(0.45-0.84)	0.003	0.02	0
cg20768743	CD226	10666	FALSE	1.6(1.18-2.18)	0.003	0.02	0

cg04645049	ARL6IP5	10550	TRUE	1.58(1.16-2.16)	0.003	0.02	0
cg12432709	CCDC68	80323	FALSE	0.64(0.48-0.86)	0.003	0.02	0
cg18572014	DCC	1630	TRUE	0.58(0.41-0.83)	0.003	0.02	0
cg04588079	HEBP1	50865	TRUE	0.6(0.43-0.84)	0.003	0.02	0
cg12447832	TTC15	51112	TRUE	1.56(1.16-2.1)	0.003	0.03	0
cg03575468	PDHA1	5160	TRUE	1.57(1.17-2.12)	0.003	0.03	0
cg17603184	C22orf5	25829	TRUE	1.75(1.22-2.52)	0.003	0.03	0
cg05342835	SYNC1	81493	FALSE	0.62(0.45-0.86)	0.003	0.03	−1
cg13351583	SHC3	53358	TRUE	1.71(1.2-2.45)	0.003	0.03	0
cg19836808	S100A7	6278	FALSE	1.58(1.17-2.13)	0.003	0.03	0
cg24516901	FLJ22746	79843	TRUE	0.62(0.45-0.85)	0.003	0.03	0
cg24989962	PTGDR	5729	TRUE	0.59(0.42-0.84)	0.003	0.03	0
cg02148834	TPTE	7179	TRUE	0.59(0.43-0.82)	0.003	0.03	0
cg26640549	RAD18	56852	TRUE	1.61(1.17-2.2)	0.003	0.03	0
cg14378057	KIAA0240	23506	FALSE	0.64(0.47-0.85)	0.003	0.03	0
cg17029168	NKX2-2	4821	TRUE	0.61(0.43-0.84)	0.003	0.03	0
cg00155485	THRAP2	23389	TRUE	1.62(1.18-2.24)	0.003	0.03	0
cg08697285	MYCBP2	23077	TRUE	1.75(1.21-2.54)	0.003	0.03	0
cg14336578	LETM2	137994	TRUE	1.59(1.17-2.16)	0.003	0.03	0
cg19219366	TRIM3	10612	TRUE	0.62(0.44-0.85)	0.003	0.03	−1
cg24691461	C20orf160	140706	FALSE	1.62(1.17-2.25)	0.003	0.03	0
cg08908355	HIST2H2AC	8338	TRUE	1.64(1.18-2.27)	0.003	0.03	0
cg17550582	SLC2A10	81031	TRUE	0.61(0.44-0.85)	0.003	0.03	0
cg04137128	SIN3A	25942	TRUE	1.67(1.19-2.35)	0.003	0.03	0
cg22836229	EFCAB1	79645	TRUE	0.61(0.45-0.85)	0.003	0.03	0
cg11373429	ICAM5	7087	TRUE	1.66(1.18-2.35)	0.003	0.03	0
cg16536918	AVP	551	FALSE	0.64(0.47-0.86)	0.003	0.03	0

cg03891319	ACY1	95	TRUE	0.6(0.42-0.85)	0.003	0.03	−1
cg11189837	ADAMTS1	9510	TRUE	0.63(0.46-0.86)	0.003	0.03	0
cg00948524	RNF135	84282	TRUE	0.6(0.43-0.83)	0.003	0.03	0
cg21750545	DCUN1D4	23142	TRUE	1.57(1.17-2.12)	0.003	0.03	0
cg02125271	SNURF	8926	TRUE	1.79(1.22-2.63)	0.003	0.03	0
cg02657721	SEMA3B	7869	FALSE	1.61(1.18-2.19)	0.003	0.03	0
cg15379633	RAB36	9609	TRUE	0.63(0.46-0.85)	0.003	0.03	0
cg18984151	TMEM103	54859	TRUE	1.64(1.18-2.28)	0.003	0.03	0
cg12762680	PSMB9	5698	TRUE	1.61(1.18-2.21)	0.003	0.03	0
cg19831077	LOC349136	349136	TRUE	0.63(0.47-0.85)	0.003	0.03	0
cg03634997	GAS8	2622	TRUE	0.63(0.46-0.86)	0.003	0.03	0
cg18919097	C3orf57	165679	TRUE	0.63(0.46-0.85)	0.003	0.03	0
cg10663017	DUSP23	54935	TRUE	1.64(1.18-2.27)	0.003	0.03	0
cg10037068	WASPIP	7456	FALSE	1.57(1.16-2.13)	0.003	0.03	0
cg12072803	JMJD2B	23030	TRUE	0.64(0.48-0.86)	0.003	0.03	0
cg23196831	COL14A1	7373	TRUE	0.58(0.41-0.83)	0.003	0.03	0
cg05684195	CIDEC	63924	FALSE	1.72(1.2-2.46)	0.003	0.03	0
cg17347253	MEST	4232	TRUE	0.61(0.44-0.85)	0.003	0.03	0
cg08317263	CCDC69	26112	TRUE	1.67(1.2-2.34)	0.003	0.03	0
cg03554552	PPP1R13L	10848	TRUE	0.61(0.45-0.84)	0.003	0.03	0
cg17063201	HCRTR2	3062	FALSE	0.63(0.47-0.86)	0.003	0.03	0
cg03742272	ALOX12B	242	FALSE	1.57(1.16-2.13)	0.003	0.03	0
cg11516606	TDRD7	23424	TRUE	0.64(0.47-0.86)	0.003	0.03	0
cg02157306	ELMOD2	255520	TRUE	1.58(1.16-2.14)	0.003	0.03	0
cg02057157	CNO	55330	TRUE	1.62(1.17-2.25)	0.003	0.03	0
cg14973995	TETRAN	10227	TRUE	0.63(0.46-0.86)	0.003	0.03	0
cg03986640	MIP	4284	FALSE	1.6(1.17-2.17)	0.003	0.03	0

cg10058540	DCTN2	10540	TRUE	1.64(1.18-2.27)	0.003	0.03	0
cg11902458	KCNN3	3782	FALSE	0.57(0.4-0.83)	0.003	0.03	0
cg06415153	PITPNM2	57605	FALSE	0.52(0.33-0.81)	0.003	0.03	0
cg12460541	ITPKA	3706	TRUE	1.69(1.2-2.37)	0.003	0.03	0
cg01493517	HOM-TES-103	25900	TRUE	1.63(1.19-2.24)	0.003	0.03	0
cg12343082	CA5A	763	FALSE	1.6(1.17-2.2)	0.003	0.03	0
cg04244987	NCKIPSD	51517	TRUE	1.74(1.22-2.47)	0.003	0.03	0
cg25824226	EBAG9	9166	TRUE	1.61(1.17-2.21)	0.003	0.03	0
cg19063972	SOX21	11166	TRUE	0.63(0.47-0.86)	0.003	0.03	0
cg15787039	SGNE1	6447	TRUE	0.63(0.47-0.86)	0.003	0.03	0
cg09313439	CDH2	1000	TRUE	0.6(0.44-0.83)	0.003	0.03	0
cg23488190	POLR2J2	246721	TRUE	1.58(1.17-2.15)	0.003	0.03	0
cg24121001	TTC16	158248	TRUE	1.73(1.2-2.5)	0.003	0.03	0
cg00245878	H-plk	51351	FALSE	0.6(0.42-0.84)	0.003	0.03	0
cg13840968	CIDEB	27141	TRUE	0.65(0.48-0.87)	0.003	0.03	−1
cg00043080	PLD3	23646	TRUE	0.63(0.47-0.86)	0.003	0.03	0
cg06148264	SERPINB1	1992	TRUE	0.63(0.46-0.86)	0.003	0.03	0
cg23591869	FLJ45803	399948	TRUE	0.59(0.41-0.84)	0.003	0.03	0
cg20089715	CACNB1	782	FALSE	0.59(0.42-0.84)	0.003	0.03	0
cg14360917	SP2	6668	FALSE	1.61(1.17-2.21)	0.003	0.03	0
cg09432376	APOL6	80830	FALSE	1.55(1.16-2.09)	0.003	0.03	0
cg04446579	ANKRD45	339416	TRUE	0.56(0.37-0.83)	0.003	0.03	0
cg04275881	SLAMF8	56833	FALSE	0.62(0.45-0.86)	0.003	0.03	0
cg00514895	CPZ	8532	TRUE	0.52(0.34-0.81)	0.003	0.03	0
cg13699808	PRKCBP1	23613	FALSE	1.59(1.16-2.17)	0.003	0.03	0
cg18017908	C17orf76	388341	FALSE	0.65(0.49-0.87)	0.003	0.03	0
cg07789083	GPRC5C	55890	TRUE	0.63(0.46-0.85)	0.003	0.03	0

cg05955224	CRI2	163126	TRUE	1.69(1.19-2.41)	0.003	0.03	0
cg21554249	SUOX	6821	FALSE	1.62(1.17-2.26)	0.003	0.03	0
cg24712395	CAPN12	147968	TRUE	0.58(0.4-0.84)	0.003	0.03	0
cg15456206	ZNF580	51157	TRUE	0.62(0.45-0.85)	0.003	0.03	0
cg04437590	NDUFA5	4698	TRUE	1.79(1.22-2.62)	0.003	0.03	0
cg23704703	C21orf13	150082	FALSE	0.64(0.47-0.86)	0.003	0.03	0
cg21085553	PARP8	79668	TRUE	1.6(1.17-2.19)	0.003	0.03	0
cg25667202	RPS27	6232	TRUE	1.74(1.2-2.53)	0.003	0.03	0
cg08432727	SOX11	6664	TRUE	0.59(0.42-0.83)	0.003	0.03	0
cg16777782	CDH13	1012	TRUE	0.63(0.46-0.86)	0.003	0.03	0
cg07297178	CEACAM7	1087	FALSE	1.66(1.19-2.33)	0.003	0.03	0
cg24477567	EPOR	2057	FALSE	0.61(0.44-0.85)	0.003	0.03	0
cg06784339	MKRN1	23608	TRUE	1.93(1.27-2.94)	0.003	0.03	0
cg24789424	BDH	622	FALSE	2.1(1.29-3.42)	0.003	0.03	0
cg22066521	ABCB5	340273	FALSE	0.58(0.41-0.84)	0.003	0.03	0
cg23392730	CHCHD1	118487	TRUE	1.64(1.17-2.29)	0.003	0.03	1
cg20304401	PABPC1	26986	TRUE	1.59(1.16-2.18)	0.003	0.03	0
cg25882366	HOXB2	3212	TRUE	0.62(0.46-0.85)	0.003	0.03	0
cg09182986	CAP1	10487	TRUE	1.61(1.17-2.23)	0.003	0.03	0
cg04956511	PTPN6	5777	TRUE	0.59(0.42-0.85)	0.003	0.03	0
cg03503905	TAF4	6874	TRUE	1.63(1.17-2.26)	0.003	0.03	0
cg01708236	NFKBIE	4794	TRUE	1.69(1.18-2.42)	0.003	0.03	0
cg22362636	TNIP2	79155	TRUE	0.63(0.46-0.86)	0.003	0.03	0
cg08293367	YPEL1	29799	TRUE	1.62(1.18-2.21)	0.003	0.03	0
cg12845808	PCDH12	51294	FALSE	0.64(0.48-0.87)	0.003	0.03	0
cg07786760	DCHS2	54798	TRUE	0.64(0.47-0.86)	0.003	0.03	0
cg26106720	STX12	23673	FALSE	0.59(0.41-0.84)	0.003	0.03	0

cg05221264	ELA2A	63036	FALSE	2.51(1.35-4.68)	0.003	0.03	0
cg01369413	UBQLN3	50613	FALSE	1.56(1.15-2.11)	0.003	0.03	0
cg21069922	ERAL1	26284	TRUE	1.54(1.15-2.07)	0.003	0.03	0
cg06933965	CMKLR1	1240	FALSE	0.63(0.46-0.87)	0.003	0.03	−1
cg16176379	AYTL1	54947	TRUE	0.62(0.45-0.86)	0.003	0.03	0
cg08023751	MERTK	10461	TRUE	0.66(0.49-0.88)	0.003	0.03	0
cg14802310	TUBA3	7846	TRUE	1.57(1.15-2.13)	0.003	0.03	0
cg26815021	SFRS2	6427	TRUE	1.85(1.23-2.8)	0.003	0.03	0
cg12385643	UGT1A6	54578	FALSE	1.63(1.17-2.27)	0.003	0.03	0
cg19994834	GRIA3	2892	TRUE	1.55(1.15-2.1)	0.003	0.03	0
cg23316360	EDNRB	1910	TRUE	0.58(0.41-0.83)	0.003	0.03	0
cg08054038	PVRL2	5819	TRUE	1.74(1.2-2.53)	0.003	0.03	0
cg06738602	PTGER2	5732	TRUE	0.61(0.43-0.85)	0.003	0.03	0
cg13297960	NCAM2	4685	TRUE	0.62(0.45-0.85)	0.003	0.03	0
cg00620857	ACOT4	122970	TRUE	0.63(0.46-0.86)	0.003	0.03	0
cg23813564	MAGEC2	51438	TRUE	0.64(0.48-0.86)	0.003	0.03	0
cg07440877	FLJ46358	400110	FALSE	1.59(1.16-2.19)	0.003	0.03	0
cg10586756	NUP93	9688	TRUE	0.58(0.41-0.84)	0.003	0.03	0
cg21846488	LCE4A	199834	TRUE	1.59(1.17-2.18)	0.003	0.03	0
cg16434546	SDCCAG10	10283	TRUE	1.73(1.2-2.51)	0.003	0.03	0
cg24820250	CLEC2A	387836	FALSE	0.6(0.42-0.84)	0.003	0.03	0
cg15869642	CBLN1	869	FALSE	0.61(0.44-0.86)	0.003	0.03	0
cg17142470	SORBS3	10174	TRUE	0.59(0.41-0.85)	0.004	0.03	−1
cg04531254	MGC17839	219902	TRUE	0.63(0.46-0.86)	0.004	0.03	0
cg06475327	CEACAM8	1088	FALSE	0.61(0.44-0.85)	0.004	0.03	0
cg21140292	SMARCA2	6595	TRUE	1.6(1.16-2.19)	0.004	0.03	0
cg25088874	BMPR1B	658	TRUE	0.61(0.44-0.84)	0.004	0.03	0

cg16220183	SEPT4	5414	TRUE	0.57(0.39-0.83)	0.004	0.03	0
cg14261309	C1orf163	65260	TRUE	1.57(1.16-2.13)	0.004	0.03	0
cg23984130		28905	FALSE	1.7(1.19-2.43)	0.004	0.03	0
cg24063382	MAS1L	116511	FALSE	1.63(1.17-2.27)	0.004	0.03	0
cg18342900	LOC388272	388272	TRUE	1.68(1.19-2.39)	0.004	0.03	0
cg21602160	AOC3	8639	FALSE	1.57(1.16-2.12)	0.004	0.03	0
cg16879596	CYP3A5	1577	FALSE	1.64(1.17-2.29)	0.004	0.03	0
cg22088594	FAM48A	55578	TRUE	1.63(1.17-2.26)	0.004	0.03	0
cg27033479	TMEM44	93109	TRUE	0.62(0.44-0.86)	0.004	0.03	−1
cg26499286	KRTAP17-1	83902	TRUE	1.55(1.15-2.08)	0.004	0.03	0
cg04926244	FLJ32894	144360	TRUE	0.65(0.49-0.87)	0.004	0.03	0
cg14383135	NPAS2	4862	TRUE	0.57(0.39-0.83)	0.004	0.03	0
cg18555206	SLC38A6	145389	TRUE	2.21(1.32-3.7)	0.004	0.03	0
cg06781209	FADS2	9415	TRUE	0.64(0.48-0.87)	0.004	0.03	0
cg23303074	LRAT	9227	TRUE	0.64(0.47-0.87)	0.004	0.03	0
cg09053680	UTF1	8433	TRUE	0.59(0.42-0.84)	0.004	0.03	0
cg05837075	ERO1L	30001	TRUE	1.59(1.16-2.18)	0.004	0.03	0
cg07204803	ARL13B	200894	TRUE	0.61(0.44-0.86)	0.004	0.03	0
cg13474750	SH3BP1	23616	FALSE	1.64(1.18-2.29)	0.004	0.03	0
cg08722720	AVEN	57099	TRUE	0.61(0.43-0.85)	0.004	0.03	0
cg14213992	ZNF546	339327	TRUE	0.62(0.45-0.86)	0.004	0.03	0
cg14123992	APOE	348	FALSE	0.62(0.45-0.86)	0.004	0.03	−1
cg20691580	APOC3	345	FALSE	1.6(1.16-2.19)	0.004	0.03	0
cg25355803	MARVELD1	83742	FALSE	0.63(0.46-0.87)	0.004	0.03	0
cg19234089	SART1	9092	TRUE	0.6(0.42-0.84)	0.004	0.03	0
cg19572242	IRAK1	3654	TRUE	1.63(1.17-2.26)	0.004	0.03	0
cg13859478	CANX	821	TRUE	1.55(1.15-2.09)	0.004	0.03	0

cg15312298	FAM84B	157638	TRUE	1.61(1.17-2.22)	0.004	0.03	0
cg16529592	RUNX3	864	FALSE	1.59(1.16-2.18)	0.004	0.03	0
cg23214764	PRSS23	11098	TRUE	0.57(0.39-0.84)	0.004	0.03	0
cg22767466	TRIM49	57093	FALSE	0.6(0.42-0.85)	0.004	0.03	0
cg14563260	EDG2	1902	TRUE	0.63(0.46-0.86)	0.004	0.03	0
cg19764436	GNAZ	2781	TRUE	0.63(0.46-0.86)	0.004	0.03	0
cg21388029	CREG2	200407	TRUE	0.63(0.47-0.86)	0.004	0.03	0
cg01078434	MAS1L	116511	FALSE	1.81(1.21-2.69)	0.004	0.03	0
cg25564800	KPNA1	3836	TRUE	1.61(1.18-2.21)	0.004	0.03	0
cg11340260	GP1BA	2811	FALSE	0.65(0.48-0.88)	0.004	0.03	0
cg20247048	FLJ32065	201283	TRUE	1.69(1.18-2.42)	0.004	0.03	0
cg15683743	SPPL2A	84888	TRUE	1.61(1.17-2.22)	0.004	0.03	0
cg26200585	PRX	57716	FALSE	0.61(0.43-0.85)	0.004	0.03	0
cg05131524	ADAM22	53616	TRUE	1.57(1.16-2.12)	0.004	0.03	0
cg03305230	RAI1	10743	TRUE	1.71(1.19-2.44)	0.004	0.03	0
cg23671708	TMEM88	92162	TRUE	0.62(0.45-0.85)	0.004	0.03	0
cg06192753	GADD45GIP1	90480	TRUE	1.63(1.17-2.26)	0.004	0.03	0
cg26767897	XDH	7498	FALSE	0.65(0.48-0.87)	0.004	0.03	0
cg25680829	LHX4	89884	TRUE	0.64(0.47-0.87)	0.004	0.03	0
cg01767116	FBXL22	283807	FALSE	1.58(1.16-2.15)	0.004	0.03	0
cg25368212	SSX1	6756	FALSE	0.63(0.46-0.87)	0.004	0.03	0
cg08554114	ERO1L	30001	TRUE	0.65(0.49-0.88)	0.004	0.03	0
cg03379131	ADAM15	8751	TRUE	0.63(0.46-0.87)	0.004	0.03	0
cg22960952	FLJ23657	152816	FALSE	1.57(1.15-2.15)	0.004	0.03	0
cg05439318	CYB5-M	80777	TRUE	0.57(0.39-0.83)	0.004	0.03	0
cg16463460	WT1	7490	TRUE	1.52(1.14-2.03)	0.004	0.03	0
cg17612991	C3	718	FALSE	0.63(0.47-0.86)	0.004	0.03	0

cg06957329	MRPS23	51649	TRUE	1.56(1.16-2.11)	0.004	0.03	0
cg08826863	VEGF	7422	TRUE	1.59(1.16-2.2)	0.004	0.03	0
cg12078929	SERHL	94009	TRUE	0.55(0.37-0.82)	0.004	0.03	0
cg14972271	CAPN2	824	TRUE	1.61(1.17-2.24)	0.004	0.03	0
cg21296602	JOSD3	79101	FALSE	0.63(0.46-0.87)	0.004	0.03	0
cg19889780	SPR	6697	TRUE	1.56(1.15-2.11)	0.004	0.03	0
cg14209518	NNMT	4837	FALSE	1.68(1.18-2.39)	0.004	0.03	0
cg00098162	ANKRD22	118932	FALSE	0.64(0.47-0.87)	0.004	0.03	0
cg15453943	RAB9P1	9366	FALSE	0.64(0.47-0.87)	0.004	0.03	0
cg08634464	LOC126295	126295	TRUE	1.54(1.15-2.08)	0.004	0.03	0
cg12789833	FBXO22	26263	TRUE	1.56(1.15-2.13)	0.004	0.03	0
cg00138126	TMEPAI	56937	FALSE	0.63(0.46-0.86)	0.004	0.03	0
cg13755070	FLI1	2313	TRUE	1.56(1.15-2.12)	0.004	0.03	0
cg15868302	FOXD2	2306	TRUE	0.62(0.46-0.85)	0.004	0.03	0
cg19944367	RGS3	5998	FALSE	1.63(1.18-2.26)	0.004	0.03	0
cg12343638	PCDHB12	56124	FALSE	1.6(1.16-2.2)	0.004	0.03	0
cg03245641	GPHA2	170589	FALSE	1.57(1.15-2.13)	0.004	0.03	0
cg12220493	TITF1	7080	TRUE	0.55(0.36-0.82)	0.004	0.03	0
cg20775959	ARNT2	9915	TRUE	0.63(0.46-0.87)	0.004	0.03	0
cg13618372	SPTBN1	6711	FALSE	0.64(0.46-0.87)	0.004	0.03	0
cg24677780	CA11	770	FALSE	0.64(0.47-0.86)	0.004	0.03	0
cg22620680	SEC22L2	26984	TRUE	1.62(1.18-2.23)	0.004	0.03	0
cg23112213	CDC23	8697	TRUE	1.56(1.16-2.11)	0.004	0.03	0
cg11316784	NSD1	64324	TRUE	0.62(0.45-0.86)	0.004	0.03	0
cg13052755	PVT1	5820	TRUE	0.6(0.42-0.85)	0.004	0.03	0
cg22858308	HIVEP2	3097	FALSE	1.59(1.15-2.2)	0.004	0.03	1
cg15786837	HOXB13	10481	TRUE	0.63(0.45-0.86)	0.004	0.03	0

cg13005002	RFPL3	10738	FALSE	1.58(1.15-2.17)	0.004	0.03	0
cg13462160	ATF4	468	TRUE	1.67(1.18-2.37)	0.004	0.03	0
cg21638219	PHYH2	26061	TRUE	1.57(1.15-2.16)	0.004	0.03	0
cg12317456	MMP2	4313	TRUE	0.64(0.47-0.87)	0.004	0.03	0
cg06042828	DDX28	55794	FALSE	0.63(0.46-0.86)	0.004	0.03	0
cg08124030	TM4SF1	4071	TRUE	0.66(0.49-0.87)	0.004	0.03	0
cg26232005	SMOX	54498	TRUE	1.58(1.15-2.15)	0.004	0.03	0
cg13179915	KCNK7	10089	FALSE	1.64(1.17-2.31)	0.004	0.03	0
cg12380854	LRRC46	90506	FALSE	1.57(1.15-2.12)	0.004	0.03	0
cg12205230	TPM3	7170	FALSE	0.62(0.44-0.86)	0.004	0.03	0
cg02497700	ZNF238	10472	TRUE	0.61(0.44-0.85)	0.004	0.03	0
cg10011232	FKBP8	23770	FALSE	1.62(1.17-2.24)	0.004	0.03	0
cg07026910	INPP5D	3635	FALSE	0.65(0.48-0.87)	0.004	0.03	0
cg05547500	TXNDC2	84203	FALSE	1.59(1.16-2.19)	0.004	0.03	0
cg01459162	PADI3	51702	TRUE	1.59(1.15-2.18)	0.004	0.03	0
cg16041660	PRICKLE1	144165	TRUE	0.56(0.39-0.81)	0.004	0.03	0
cg24582500	TXNL2	10539	TRUE	1.79(1.22-2.63)	0.004	0.03	0
cg19327844	APBB1	322	TRUE	1.65(1.17-2.32)	0.004	0.03	0
cg04887278	RWDD2	112611	TRUE	0.64(0.47-0.87)	0.004	0.03	0
cg04057106	C9orf48	347240	FALSE	0.66(0.49-0.88)	0.004	0.03	0
cg27462398	C1orf181	54680	FALSE	0.6(0.43-0.85)	0.004	0.03	0
cg12163490	CDH11	1009	TRUE	0.62(0.45-0.85)	0.004	0.03	0
cg14080001	SH3PX3	257364	TRUE	0.62(0.44-0.86)	0.004	0.03	0
cg06713098	IGFBP3	3486	TRUE	0.64(0.47-0.86)	0.004	0.03	0
cg13847113	MGC24975	163154	FALSE	0.63(0.47-0.86)	0.004	0.03	0
cg26596161	SLC26A7	115111	FALSE	1.83(1.2-2.8)	0.004	0.03	0
cg15185794	PADI2	11240	TRUE	0.63(0.46-0.87)	0.004	0.03	0

cg14564494	CBR3	874	FALSE	0.63(0.46-0.87)	0.004	0.03	0
cg18195628	MRPL48	51642	TRUE	1.58(1.15-2.18)	0.004	0.03	0
cg17925542	IDH2	3418	TRUE	0.62(0.44-0.86)	0.004	0.03	0
cg15170903	CROP	51747	TRUE	1.62(1.17-2.24)	0.004	0.03	0
cg07873128	OSBPL5	114879	TRUE	1.77(1.23-2.55)	0.004	0.03	0
cg15603885	ABRA	137735	FALSE	0.64(0.47-0.87)	0.004	0.03	0
cg13744194	OCLN	4950	TRUE	0.62(0.44-0.86)	0.004	0.03	0
cg19061982	POLR1B	84172	TRUE	1.57(1.15-2.15)	0.004	0.03	0
cg01769037	JARID2	3720	FALSE	2.07(1.25-3.42)	0.004	0.03	0
cg10238818	CYYR1	116159	TRUE	0.63(0.46-0.86)	0.004	0.03	0
cg26833169	CALCA	796	TRUE	1.8(1.2-2.7)	0.004	0.03	0
cg04119538	TOP1	7150	TRUE	1.62(1.17-2.25)	0.004	0.03	0
cg06585027	PIK4CB	5298	TRUE	1.56(1.15-2.11)	0.004	0.03	0
cg03852570	C10orf33	84795	FALSE	0.64(0.47-0.87)	0.004	0.03	0
cg14377593	TFPI2	7980	TRUE	0.59(0.42-0.84)	0.004	0.03	0
cg11808544	FKBP9L	360132	TRUE	1.61(1.17-2.23)	0.004	0.03	0
cg00884529	BZW2	28969	TRUE	1.58(1.16-2.15)	0.004	0.03	0
cg22019980	RNF146	81847	TRUE	1.69(1.17-2.44)	0.004	0.03	0
cg22296149	AMH	268	FALSE	0.65(0.48-0.88)	0.004	0.03	0
cg02053171	WBSCR16	81554	TRUE	1.58(1.16-2.17)	0.004	0.03	0
cg13185177	GP5	2814	FALSE	0.65(0.48-0.88)	0.004	0.03	−1
cg15433631	IRX2	153572	TRUE	0.63(0.46-0.85)	0.004	0.03	0
cg17751569	CD180	4064	FALSE	0.64(0.46-0.87)	0.004	0.03	0
cg15536242	MIP	4284	FALSE	0.64(0.47-0.87)	0.004	0.03	0
cg20052718	TWIST1	7291	TRUE	0.51(0.33-0.8)	0.004	0.03	0
cg03014628	RAB3C	115827	TRUE	0.65(0.49-0.87)	0.004	0.03	0
cg22837289	MDS028	55846	TRUE	1.57(1.15-2.15)	0.004	0.03	0

cg21096399	MCAM	4162	TRUE	0.64(0.48-0.87)	0.004	0.03	0
cg21973276	SMAD6	4091	TRUE	1.56(1.15-2.12)	0.004	0.03	0
cg14254419	AMPD2	271	TRUE	0.61(0.44-0.86)	0.004	0.03	0
cg04806409	TFF3	7033	FALSE	1.63(1.17-2.28)	0.004	0.03	0
cg21307628	URB	151887	FALSE	0.61(0.43-0.86)	0.004	0.03	0
cg02775617	ARHGAP12	94134	TRUE	1.61(1.15-2.26)	0.004	0.03	0
cg00504595	TNFRSF19	55504	FALSE	1.51(1.13-2.02)	0.004	0.03	0
cg26133068	SLC2A11	66035	TRUE	0.61(0.43-0.86)	0.004	0.03	0
cg02626929	PAQR4	124222	FALSE	0.62(0.44-0.86)	0.004	0.03	0
cg27486427	RARB	5915	TRUE	0.61(0.44-0.86)	0.004	0.03	0
cg08136806	KRT6E	286887	FALSE	1.54(1.14-2.07)	0.004	0.03	0
cg13060997	CCNA1	8900	TRUE	0.64(0.47-0.87)	0.004	0.03	0
cg01594214	SMPDL3A	10924	TRUE	1.64(1.17-2.29)	0.004	0.03	0
cg00888561	WDFY2	115825	TRUE	0.59(0.41-0.85)	0.004	0.03	0
cg23724447	BUB3	9184	TRUE	1.61(1.16-2.24)	0.004	0.03	0
cg06507244	DHX32	55760	FALSE	0.64(0.46-0.87)	0.004	0.03	0
cg27303882	PAGE2	203569	TRUE	1.52(1.13-2.03)	0.004	0.03	0
cg07404485	PON1	5444	FALSE	1.57(1.16-2.12)	0.004	0.03	0
cg21201109	ANKRD25	25959	FALSE	0.66(0.5-0.88)	0.004	0.03	0
cg13615396	RACGAP1	29127	TRUE	1.59(1.15-2.21)	0.004	0.03	0
cg19797376	TAL1	6886	TRUE	0.65(0.48-0.87)	0.004	0.03	0
cg26675382	NUP43	348995	TRUE	1.57(1.15-2.14)	0.004	0.03	0
cg18988110	ATAD4	79170	FALSE	1.55(1.15-2.08)	0.004	0.03	0
cg06975499	EZH1	2145	TRUE	1.6(1.15-2.23)	0.004	0.03	0
cg11414151	ACSL1	2180	TRUE	1.6(1.16-2.22)	0.004	0.03	0
cg08080029	CHD5	26038	TRUE	0.61(0.43-0.85)	0.004	0.03	0
cg13494498	TUBB3	10381	TRUE	1.63(1.16-2.3)	0.004	0.03	0

cg08360728	GPATC3	63906	TRUE	1.8(1.19-2.71)	0.004	0.03	0
cg04884908	CYP26B1	56603	TRUE	0.63(0.46-0.86)	0.004	0.03	0
cg15280964	RBM34	23029	TRUE	1.83(1.21-2.79)	0.004	0.03	0
cg27087809	ACSBG1	23205	FALSE	1.54(1.14-2.08)	0.004	0.03	0
cg23042138	HYPB	29072	FALSE	1.59(1.15-2.21)	0.004	0.03	0
cg20833786	MRGPRX3	117195	FALSE	0.64(0.46-0.87)	0.004	0.03	0
cg11951066	FLJ14768	84922	FALSE	0.66(0.49-0.88)	0.004	0.03	0
cg07714374	TCF4	6925	TRUE	1.64(1.16-2.33)	0.004	0.03	0
cg21039631	CD58	965	TRUE	0.65(0.48-0.88)	0.004	0.03	0
cg15530356	ZNF414	84330	TRUE	0.63(0.46-0.87)	0.004	0.03	0
cg17027046	ITPA	3704	TRUE	1.6(1.16-2.21)	0.004	0.03	0
cg15250507	C1orf106	55765	TRUE	0.63(0.46-0.86)	0.004	0.03	0
cg21111471	FLRT1	23769	FALSE	1.71(1.18-2.49)	0.005	0.03	0
cg07496902	ASB14	142686	FALSE	0.63(0.45-0.87)	0.005	0.03	0
cg14036856	MGC52423	149466	FALSE	1.55(1.14-2.11)	0.005	0.03	0
cg26753137	SIRT5	23408	TRUE	1.56(1.14-2.13)	0.005	0.03	0
cg00557354	ARHGEF7	8874	TRUE	0.62(0.45-0.86)	0.005	0.03	0
cg02124291	OR7A5	26659	FALSE	0.63(0.46-0.87)	0.005	0.03	0
cg24594997	RBP1	5947	TRUE	1.63(1.16-2.3)	0.005	0.03	0
cg25724441	KCNJ2	3759	TRUE	0.6(0.43-0.85)	0.005	0.03	0
cg16360372	SPINK1	6690	FALSE	0.59(0.4-0.85)	0.005	0.03	0
cg11270633	IL28B	282617	FALSE	1.6(1.16-2.2)	0.005	0.03	0
cg02148642	RGPD5	84220	FALSE	1.62(1.16-2.25)	0.005	0.03	0
cg10159529	IL5RA	3568	FALSE	1.57(1.14-2.16)	0.005	0.03	0
cg09985635	PCDH1	5097	TRUE	1.53(1.14-2.06)	0.005	0.03	0
cg14577211	ZNF641	121274	TRUE	1.57(1.14-2.15)	0.005	0.03	0
cg00939965	PRRG1	5638	FALSE	0.62(0.45-0.86)	0.005	0.03	0

cg18305837	COQ7	10229	TRUE	1.61(1.16-2.23)	0.005	0.03	0
cg07117700	PSMC5	5705	FALSE	0.59(0.41-0.85)	0.005	0.03	0
cg17833578	LRRC21	26103	FALSE	1.54(1.14-2.08)	0.005	0.03	0
cg01352108	KCNK4	50801	TRUE	0.64(0.48-0.87)	0.005	0.03	0
cg24703623	CLCC1	23155	TRUE	1.71(1.18-2.47)	0.005	0.03	0
cg06060661	MAGEA11	4110	FALSE	1.53(1.14-2.06)	0.005	0.03	0
cg05125838	UCN2	90226	FALSE	0.66(0.49-0.88)	0.005	0.03	0
cg09721659	TSHR	7253	TRUE	1.52(1.13-2.04)	0.005	0.03	0
cg16046465	FRMD5	84978	TRUE	0.6(0.42-0.84)	0.005	0.03	0
cg10784813	SOCS1	8651	TRUE	1.55(1.14-2.11)	0.005	0.03	0
cg12099051	SAMHD1	25939	TRUE	1.63(1.17-2.28)	0.005	0.03	0
cg07326586	UBD	10537	FALSE	1.58(1.15-2.16)	0.005	0.03	0
cg21032583	LMLN	89782	TRUE	0.63(0.46-0.87)	0.005	0.03	0
cg03406535	RBP7	116362	FALSE	0.66(0.49-0.88)	0.005	0.03	0
cg07285953	CRYBA1	1411	FALSE	0.63(0.46-0.87)	0.005	0.03	0
cg22740783	CGREF1	10669	FALSE	0.63(0.45-0.87)	0.005	0.03	0
cg20033731	CACNA1C	775	TRUE	0.6(0.41-0.86)	0.005	0.03	0
cg15774153	FGF19	9965	TRUE	0.6(0.42-0.85)	0.005	0.03	0
cg15764655	SBF1	6305	TRUE	0.65(0.48-0.88)	0.005	0.03	0
cg27257408	ACSBG1	23205	FALSE	1.6(1.16-2.2)	0.005	0.03	0
cg19430430	COL5A3	50509	TRUE	0.58(0.39-0.85)	0.005	0.03	0
cg07336230	KIF6	221458	TRUE	0.58(0.4-0.85)	0.005	0.03	0
cg16882703	PSMB6	5694	TRUE	1.59(1.15-2.21)	0.005	0.03	0
cg24597988	SELL	6402	FALSE	0.64(0.46-0.87)	0.005	0.03	0
cg09054876	PECI	10455	TRUE	1.58(1.14-2.18)	0.005	0.03	0
cg07048066	C5orf5	51306	TRUE	1.57(1.15-2.15)	0.005	0.03	0
cg10210238	CDKN2B	1030	TRUE	0.63(0.46-0.86)	0.005	0.03	0

cg21303011	THRB	7068	TRUE	0.61(0.44-0.86)	0.005	0.03	0
cg11458974	HMOX1	3162	FALSE	0.64(0.47-0.87)	0.005	0.03	0
cg26416466	MEGF11	84465	TRUE	0.59(0.4-0.85)	0.005	0.03	0
cg25557858	EXTL3	2137	TRUE	1.64(1.17-2.3)	0.005	0.03	0
cg01269795	BTN3A3	10384	FALSE	1.58(1.15-2.16)	0.005	0.03	0
cg24392574	CALML5	51806	TRUE	0.63(0.46-0.87)	0.005	0.03	0
cg24468890	HDAC1	3065	TRUE	1.7(1.18-2.46)	0.005	0.03	0
cg15507817	WNT7B	7477	TRUE	0.61(0.43-0.86)	0.005	0.04	0
cg04556854	FLJ35630	166379	TRUE	1.61(1.15-2.24)	0.005	0.04	0
cg08466883	KCTD3	51133	TRUE	0.61(0.44-0.86)	0.005	0.04	0
cg01459453	SELP	6403	FALSE	0.66(0.49-0.89)	0.005	0.04	0
cg26911787	ELK3	2004	TRUE	0.62(0.44-0.87)	0.005	0.04	0
cg19784449	WASF2	10163	TRUE	1.54(1.14-2.09)	0.005	0.04	0
cg16873863	SLC22A18	5002	FALSE	0.67(0.5-0.89)	0.005	0.04	0
cg09658342	TIA1	7072	TRUE	1.78(1.2-2.65)	0.005	0.04	0
cg06913228	UBE2J1	51465	TRUE	1.57(1.14-2.16)	0.005	0.04	0
cg24101359	GNMT	27232	TRUE	0.63(0.46-0.87)	0.005	0.04	0
cg23079782	P2RY10	27334	FALSE	1.61(1.16-2.25)	0.005	0.04	0
cg06288351	KCNS1	3787	TRUE	0.65(0.48-0.88)	0.005	0.04	−1
cg11768886	STK32B	55351	TRUE	1.57(1.15-2.14)	0.005	0.04	0
cg04796162	IGFBP3	3486	TRUE	0.59(0.41-0.85)	0.005	0.04	0
cg15783800	HAK	115701	TRUE	0.64(0.47-0.87)	0.005	0.04	0
cg06598631	ORC3L	23595	TRUE	1.56(1.15-2.1)	0.005	0.04	0
cg17525003	IKIP	121457	TRUE	1.57(1.15-2.15)	0.005	0.04	0
cg06751597	SNAP23	8773	TRUE	1.63(1.16-2.3)	0.005	0.04	0
cg18979491	LBP	3929	FALSE	1.54(1.14-2.06)	0.005	0.04	0
cg02990033	GFRA4	64096	FALSE	0.65(0.47-0.88)	0.005	0.04	0

cg00582628	RGS20	8601	FALSE	1.57(1.16-2.12)	0.005	0.04	0
cg05067973	TRIB3	57761	FALSE	0.65(0.48-0.88)	0.005	0.04	0
cg16772514	C1QL1	10882	TRUE	0.65(0.48-0.88)	0.005	0.04	0
cg16543027	PLCB2	5330	FALSE	0.59(0.4-0.86)	0.005	0.04	0
cg16896647	SYK	6850	TRUE	0.63(0.45-0.87)	0.005	0.04	0
cg16799087	PRDM7	11105	FALSE	1.59(1.15-2.18)	0.005	0.04	0
cg27379587	NMB	4828	TRUE	1.68(1.17-2.4)	0.005	0.04	0
cg09542111	GRIK1	2897	TRUE	0.6(0.42-0.85)	0.005	0.04	0
cg10276576	C11orf24	53838	TRUE	1.6(1.15-2.23)	0.005	0.04	0
cg05501584	ST8SIA2	8128	TRUE	1.56(1.14-2.14)	0.005	0.04	0
cg22012981	ACOX2	8309	FALSE	0.62(0.44-0.87)	0.005	0.04	−1
cg04891836	TNFSF14	8740	FALSE	1.53(1.13-2.07)	0.005	0.04	0
cg09536738	EFHD1	80303	TRUE	1.56(1.14-2.15)	0.005	0.04	0
cg25411725	SLC22A13	9390	FALSE	0.63(0.46-0.86)	0.005	0.04	0
cg25499099	TNK1	8711	TRUE	0.65(0.48-0.89)	0.005	0.04	0
cg10840135	CYP2D6	1565	FALSE	1.57(1.15-2.15)	0.005	0.04	0
cg19844287	MOCS2	4338	TRUE	0.58(0.4-0.84)	0.005	0.04	0
cg25107791	CLPS	1208	FALSE	1.57(1.14-2.15)	0.005	0.04	0
cg05897048	HRASLS3	11145	TRUE	1.58(1.17-2.15)	0.005	0.04	0
cg09027725	COX4I2	84701	FALSE	1.62(1.16-2.27)	0.005	0.04	0
cg24133115	PDE10A	10846	TRUE	0.59(0.41-0.84)	0.005	0.04	0
cg01782486	ZBTB7B	51043	FALSE	1.6(1.14-2.24)	0.005	0.04	0
cg03404502	GPR18	2841	FALSE	0.65(0.48-0.88)	0.005	0.04	0
cg07846220	LAMA1	284217	TRUE	0.63(0.46-0.86)	0.005	0.04	0
cg18951427	ACYP2	98	FALSE	0.48(0.29-0.81)	0.005	0.04	0
cg17520176	ZMYM4	9202	TRUE	1.57(1.14-2.15)	0.005	0.04	0
cg00024396	ELOVL5	60481	TRUE	1.68(1.17-2.42)	0.005	0.04	0

cg05554936	RAB3D	9545	TRUE	1.54(1.14-2.07)	0.005	0.04	0
cg08474603	CRP	1401	FALSE	0.62(0.44-0.87)	0.005	0.04	0
cg00563932	PTGDS	5730	FALSE	0.64(0.46-0.88)	0.005	0.04	−1
cg10150813	KIAA0746	23231	TRUE	0.64(0.47-0.87)	0.005	0.04	0
cg23001650	B4GALT2	8704	TRUE	0.64(0.46-0.88)	0.005	0.04	0
cg07973390	CARF	55602	TRUE	1.64(1.17-2.32)	0.005	0.04	0
cg01313514	WNT3A	89780	TRUE	0.6(0.43-0.85)	0.005	0.04	0
cg02154074	HTRA2	27429	TRUE	1.52(1.13-2.05)	0.005	0.04	0
cg06403553	PGK2	5232	FALSE	1.61(1.16-2.25)	0.005	0.04	0
cg20189782	MGC27121	408263	FALSE	1.6(1.16-2.22)	0.005	0.04	0
cg12204727	COMMD4	54939	TRUE	1.56(1.14-2.14)	0.005	0.04	0
cg17302852	FXR1	8087	TRUE	1.51(1.12-2.03)	0.005	0.04	0
cg04605667	SIAH2	6478	TRUE	1.66(1.16-2.37)	0.005	0.04	0
cg24924631	BCL2A1	597	TRUE	0.58(0.39-0.85)	0.005	0.04	0
cg19721889	HAND1	9421	TRUE	0.62(0.45-0.86)	0.005	0.04	0
cg05595345	ARRDC4	91947	TRUE	0.62(0.45-0.86)	0.005	0.04	0
cg01587454	WDR21C	138009	TRUE	0.59(0.41-0.85)	0.005	0.04	0
cg04623837	HCG9	10255	TRUE	0.64(0.47-0.88)	0.005	0.04	0
cg06433658	FAM63A	55793	FALSE	0.64(0.46-0.88)	0.005	0.04	0
cg17384145	HIST1H2AM	8336	TRUE	1.57(1.15-2.13)	0.005	0.04	0
cg02342494	MGC50559	254013	TRUE	1.54(1.13-2.09)	0.005	0.04	0
cg03278960	C16orf57	79650	TRUE	1.51(1.13-2)	0.005	0.04	0
cg22090592	C14orf173	64423	FALSE	0.64(0.46-0.88)	0.005	0.04	0
cg13253729	Rgr	266747	TRUE	0.65(0.48-0.87)	0.005	0.04	0
cg26523005	ZNF662	389114	FALSE	0.66(0.49-0.88)	0.005	0.04	0
cg10025443	SYK	6850	TRUE	0.64(0.47-0.88)	0.005	0.04	0
cg11051158	C2orf3	6936	TRUE	1.55(1.14-2.11)	0.005	0.04	0

cg15557833	MGC33302	256471	TRUE	1.72(1.17-2.52)	0.005	0.04	0
cg08452348	CATSPER3	347732	FALSE	0.63(0.46-0.88)	0.005	0.04	0
cg02876062	FAM107B	83641	TRUE	1.58(1.15-2.16)	0.005	0.04	0
cg02440177	ZNF702	79986	TRUE	0.64(0.47-0.87)	0.005	0.04	0
cg00468146	ID4	3400	TRUE	0.63(0.45-0.86)	0.005	0.04	0
cg19655070	HEXIM2	124790	TRUE	1.61(1.15-2.26)	0.005	0.04	0
cg12893143	UNC5A	90249	TRUE	0.62(0.45-0.86)	0.005	0.04	0
cg23508786	IFNGR2	3460	TRUE	1.53(1.13-2.06)	0.005	0.04	0
cg21429500	LAP3	51056	TRUE	1.65(1.17-2.33)	0.005	0.04	0
cg20959866	AJAP1	55966	TRUE	0.57(0.38-0.84)	0.005	0.04	0
cg26571739	VAV1	7409	TRUE	1.69(1.18-2.43)	0.005	0.04	0
cg10107671	FLJ45684	400666	TRUE	1.64(1.16-2.32)	0.005	0.04	0
cg00690280	WFDC10B	280664	FALSE	1.53(1.13-2.06)	0.005	0.04	0
cg20030671	FBXO28	23219	TRUE	1.53(1.15-2.04)	0.005	0.04	0
cg10035922	C1orf102	127700	TRUE	1.55(1.14-2.11)	0.005	0.04	0
cg08558873	ZNF214	7761	TRUE	0.58(0.39-0.84)	0.005	0.04	0
cg04790129	ITGB2	3689	FALSE	0.66(0.49-0.88)	0.005	0.04	0
cg12952132	NCR1	9437	FALSE	1.55(1.14-2.12)	0.005	0.04	0
cg17436805	MIPEP	4285	TRUE	1.6(1.15-2.23)	0.005	0.04	0
cg26845300	SNX9	51429	TRUE	0.61(0.43-0.86)	0.005	0.04	0
cg17034109	CYB561D1	284613	TRUE	0.65(0.47-0.89)	0.005	0.04	−1
cg26609631	GSH1	219409	TRUE	0.61(0.44-0.85)	0.005	0.04	0
cg16721202	SP4	6671	TRUE	1.59(1.14-2.2)	0.005	0.04	0
cg08596000	PDGFD	80310	TRUE	0.65(0.47-0.89)	0.005	0.04	−1
cg23558650	PPME1	51400	TRUE	1.6(1.15-2.23)	0.005	0.04	0
cg25737491	GABARAP	11337	TRUE	1.59(1.14-2.22)	0.005	0.04	0
cg03440846	ACSS2	55902	FALSE	0.64(0.47-0.89)	0.005	0.04	0

cg18833140	HABP2	3026	FALSE	0.65(0.48-0.89)	0.005	0.04	0
cg15379858	ChGn	55790	FALSE	0.66(0.49-0.88)	0.005	0.04	0
cg05308617	ARMC8	25852	TRUE	1.54(1.13-2.1)	0.005	0.04	0
cg09425215	CHD2	1106	TRUE	1.61(1.14-2.26)	0.005	0.04	0
cg24073051	CDV3	55573	TRUE	1.54(1.13-2.1)	0.005	0.04	0
cg22495120	FMR1NB	158521	TRUE	0.66(0.49-0.89)	0.005	0.04	0
cg04770504	DFNA5	1687	TRUE	0.65(0.48-0.87)	0.005	0.04	0
cg27038439	MSX1	4487	TRUE	0.65(0.48-0.88)	0.005	0.04	0
cg01797043	RPL3L	6123	FALSE	0.65(0.48-0.89)	0.005	0.04	−1
cg25748127	POLR2L	5441	TRUE	1.55(1.13-2.12)	0.005	0.04	0
cg13397379	OR2C3	81472	FALSE	1.57(1.14-2.17)	0.005	0.04	0
cg06277277	NR1I3	9970	FALSE	2.37(1.29-4.38)	0.005	0.04	0
cg13121699	C2orf10	91752	TRUE	0.65(0.48-0.88)	0.005	0.04	0
cg21751787	LMAN2	10960	TRUE	1.48(1.12-1.95)	0.005	0.04	0
cg13454184	TARSL1	80222	TRUE	1.56(1.14-2.14)	0.005	0.04	0
cg18003791	GABRA3	2556	FALSE	1.54(1.13-2.1)	0.005	0.04	0
cg04278905	GCN5L2	2648	TRUE	0.63(0.46-0.88)	0.005	0.04	0
cg12506373	CAD	790	TRUE	1.52(1.13-2.06)	0.005	0.04	0
cg05921207	CHRDL1	91851	TRUE	1.55(1.14-2.12)	0.006	0.04	0
cg18717447	LOC159090	159090	TRUE	1.51(1.13-2.03)	0.006	0.04	0
cg03294491	SMAD2	4087	FALSE	0.63(0.46-0.87)	0.006	0.04	0
cg07150830	NOS2A	4843	FALSE	0.64(0.46-0.88)	0.006	0.04	0
cg07073964	PRSSL1	400668	TRUE	0.67(0.51-0.89)	0.006	0.04	0
cg16256230	KIF4A	24137	TRUE	1.51(1.12-2.02)	0.006	0.04	0
cg15006396	NFKBIZ	64332	TRUE	1.59(1.13-2.22)	0.006	0.04	0
cg01103730	IL20	50604	FALSE	0.64(0.47-0.88)	0.006	0.04	0
cg05679613	MEPE	56955	FALSE	0.62(0.44-0.88)	0.006	0.04	0

cg17903544	PGAM5	192111	TRUE	0.61(0.42-0.86)	0.006	0.04	0
cg06838394	ARFGEF2	10564	TRUE	1.59(1.14-2.2)	0.006	0.04	0
cg05798972	PPARBP	5469	FALSE	1.52(1.13-2.06)	0.006	0.04	0
cg23667432	ALPP	250	TRUE	1.55(1.13-2.12)	0.006	0.04	0
cg06536578	JPH4	84502	FALSE	0.61(0.43-0.87)	0.006	0.04	0
cg26778754	SSFA2	6744	TRUE	1.57(1.14-2.17)	0.006	0.04	0
cg22034555	DHX36	170506	TRUE	1.55(1.13-2.11)	0.006	0.04	0
cg20277416	TM7SF2	7108	TRUE	1.59(1.15-2.19)	0.006	0.04	0
cg10983208	SPOCK2	9806	TRUE	1.58(1.14-2.18)	0.006	0.04	0
cg03011500	CGN	57530	TRUE	1.58(1.14-2.19)	0.006	0.04	0
cg22396755	RAP1GA1	5909	TRUE	0.66(0.49-0.89)	0.006	0.04	0
cg24664861	SPAG16	79582	TRUE	1.55(1.13-2.13)	0.006	0.04	0
cg18015044	ABCF1	23	TRUE	1.58(1.14-2.18)	0.006	0.04	0
cg13850625	FLJ11155	55314	TRUE	0.44(0.25-0.78)	0.006	0.04	0
cg13164537	CD226	10666	FALSE	1.5(1.12-2.02)	0.006	0.04	1
cg24890286	HIPK2	28996	FALSE	0.61(0.43-0.87)	0.006	0.04	0
cg13226591	MAGEB18	286514	TRUE	0.66(0.48-0.89)	0.006	0.04	0
cg01899253	FLT1	2321	TRUE	0.65(0.48-0.89)	0.006	0.04	−1
cg15736165	BNC1	646	TRUE	1.6(1.14-2.23)	0.006	0.04	0
cg00350296	CD248	57124	FALSE	0.66(0.49-0.89)	0.006	0.04	0
cg08749122	TARSL1	80222	TRUE	1.56(1.13-2.14)	0.006	0.04	0
cg09018040	VCX	26609	FALSE	0.65(0.48-0.88)	0.006	0.04	0
cg26619317	CNN3	1266	TRUE	0.65(0.48-0.88)	0.006	0.04	0
cg04387658	CD86	942	TRUE	0.65(0.48-0.88)	0.006	0.04	0
cg17367215	SUPT5H	6829	TRUE	1.53(1.13-2.06)	0.006	0.04	0
cg05700681	CCL22	6367	FALSE	1.66(1.17-2.36)	0.006	0.04	0
cg22040627	SLC13A5	284111	TRUE	0.64(0.47-0.88)	0.006	0.04	0

cg19355685	ZFAND2B	130617	TRUE	1.62(1.15-2.26)	0.006	0.04	0
cg01720520	LILRB1	10859	FALSE	1.54(1.13-2.11)	0.006	0.04	0
cg03065661	CRSP6	9440	TRUE	1.59(1.15-2.21)	0.006	0.04	0
cg27118825	RSHL1	81492	TRUE	0.6(0.42-0.86)	0.006	0.04	0
cg24164563	FOXJ1	2302	TRUE	0.64(0.46-0.88)	0.006	0.04	0
cg24453353	HIST1H4C	8364	TRUE	1.62(1.16-2.25)	0.006	0.04	0
cg17243643	RDH5	5959	FALSE	0.66(0.49-0.89)	0.006	0.04	0
cg12906740	NUDT15	55270	TRUE	1.55(1.13-2.11)	0.006	0.04	0
cg27589921	CIAPIN1	57019	FALSE	0.62(0.44-0.88)	0.006	0.04	0
cg25156443	SFRP5	6425	TRUE	1.58(1.15-2.18)	0.006	0.04	0
cg01930621	ZNF649	65251	TRUE	1.5(1.12-2)	0.006	0.04	0
cg24337809	BTG2	7832	TRUE	1.52(1.14-2.02)	0.006	0.04	0
cg12213910	RAET1E	135250	FALSE	1.55(1.13-2.12)	0.006	0.04	0
cg03608577	OR12D3	81797	FALSE	1.53(1.13-2.08)	0.006	0.04	0
cg11628487	SET	6418	TRUE	0.66(0.49-0.89)	0.006	0.04	−1
cg03343942	SLC39A5	283375	FALSE	1.57(1.14-2.16)	0.006	0.04	0
cg18694780	CMKOR1	57007	TRUE	0.65(0.47-0.89)	0.006	0.04	0
cg25230532	GALT	2592	TRUE	1.58(1.14-2.21)	0.006	0.04	0
cg09257092	ITPR2	3709	TRUE	0.65(0.48-0.88)	0.006	0.04	0
cg19552482	ZNF691	51058	TRUE	1.62(1.16-2.27)	0.006	0.04	0
cg02580195	RABL2A	11159	TRUE	1.8(1.22-2.64)	0.006	0.04	0
cg10763288	DHCR7	1717	TRUE	0.66(0.49-0.89)	0.006	0.04	0
cg02008169	ZNF267	10308	TRUE	1.61(1.15-2.27)	0.006	0.04	0
cg17100200	GUCA2B	2981	FALSE	1.59(1.14-2.23)	0.006	0.04	0
cg17338403	SLCO3A1	28232	FALSE	0.64(0.46-0.88)	0.006	0.04	0
cg17469479	CLEC9A	283420	TRUE	0.66(0.49-0.89)	0.006	0.04	0
cg21275690	WDR58	79228	FALSE	0.65(0.48-0.89)	0.006	0.04	0

cg22563815	CHRNA5	1138	TRUE	0.67(0.5-0.9)	0.006	0.04	−1
cg05681757	FGD4	121512	FALSE	0.64(0.47-0.89)	0.006	0.04	0
cg17559549	MAPK7	5598	TRUE	1.6(1.15-2.23)	0.006	0.04	0
cg00881086	NPHP3	27031	TRUE	1.52(1.13-2.05)	0.006	0.04	0
cg11403598	COG8	84342	FALSE	1.54(1.13-2.09)	0.006	0.04	0
cg15376097	EVA1	10205	FALSE	0.66(0.49-0.89)	0.006	0.04	0
cg03026462	FOXA1	3169	TRUE	0.64(0.47-0.88)	0.006	0.04	0
cg26220985	DPT	1805	FALSE	1.58(1.13-2.21)	0.006	0.04	0
cg05774801	SFRP2	6423	TRUE	1.54(1.13-2.09)	0.006	0.04	0
cg13003163	ANP32D	23519	TRUE	0.59(0.41-0.85)	0.006	0.04	0
cg10100220	LNX2	222484	FALSE	0.63(0.45-0.88)	0.006	0.04	0
cg23391006	OXTR	5021	TRUE	0.64(0.46-0.88)	0.006	0.04	0
cg13159566	CCDC59	29080	TRUE	0.64(0.47-0.89)	0.006	0.04	−1
cg04826883	CA12	771	TRUE	1.64(1.16-2.31)	0.006	0.04	0
cg08251399	EHD3	30845	TRUE	0.65(0.48-0.88)	0.006	0.04	0
cg15062535	ZNF610	162963	FALSE	1.58(1.14-2.21)	0.006	0.04	0
cg07664027	RPL13A	23521	TRUE	0.61(0.43-0.87)	0.006	0.04	0
cg26259865	LOC124220	124220	FALSE	0.64(0.46-0.89)	0.006	0.04	0
cg23326197	CYP3A4	1576	FALSE	1.49(1.12-1.98)	0.006	0.04	0
cg15559700	UGT1A9	54600	FALSE	0.64(0.47-0.88)	0.006	0.04	0
cg11203041	MGST1	4257	FALSE	0.66(0.49-0.89)	0.006	0.04	0
cg11158729	CNNM4	26504	TRUE	0.62(0.44-0.88)	0.006	0.04	0
cg23765993	SPINLW1	57119	FALSE	1.52(1.12-2.05)	0.006	0.04	0
cg21164303	TMED3	23423	TRUE	0.66(0.49-0.89)	0.006	0.04	0
cg07843027	LOC124491	124491	TRUE	1.54(1.13-2.11)	0.006	0.04	0
cg02930996	B4GALT6	9331	TRUE	0.61(0.44-0.86)	0.006	0.04	0
cg09371142	DCUN1D3	123879	TRUE	1.58(1.14-2.19)	0.006	0.04	0

cg05112986	SUPT3H	8464	TRUE	1.55(1.13-2.14)	0.006	0.04	0
cg04418492	CYP7B1	9420	TRUE	0.64(0.47-0.88)	0.006	0.04	0
cg05826823	CIZ1	25792	FALSE	0.67(0.5-0.89)	0.006	0.04	0
cg09421562	MPO	4353	FALSE	0.66(0.49-0.89)	0.006	0.04	0
cg05521696	SLC2A14	144195	TRUE	0.6(0.42-0.86)	0.006	0.04	0
cg11745019	ANGPT1	284	TRUE	1.63(1.14-2.33)	0.006	0.04	0
cg07897701	ABP1	26	FALSE	0.67(0.5-0.89)	0.006	0.04	0
cg00746130	BAT5	7920	TRUE	1.51(1.12-2.04)	0.006	0.04	0
cg05512756	UGT3A1	133688	FALSE	1.54(1.13-2.1)	0.006	0.04	0
cg24287460	CCDC48	79825	TRUE	1.56(1.14-2.14)	0.006	0.04	0
cg13287780	WRB	7485	TRUE	0.63(0.45-0.88)	0.006	0.04	0
cg12400881	PPL	5493	TRUE	0.67(0.5-0.89)	0.006	0.04	0
cg02164386	HIGD1B	51751	FALSE	0.64(0.46-0.88)	0.006	0.04	0
cg25946952	KIAA0773	9715	TRUE	1.73(1.16-2.56)	0.006	0.04	0
cg19423196	MAT1A	4143	FALSE	1.51(1.12-2.03)	0.006	0.04	0
cg08285151	HDAC9	9734	FALSE	1.55(1.13-2.13)	0.006	0.04	0
cg20240860	PHACS	84680	TRUE	1.56(1.15-2.12)	0.006	0.04	0
cg10633491	ZBTB7A	51341	TRUE	1.52(1.12-2.06)	0.006	0.04	0
cg23504707	PPM1A	5494	FALSE	0.65(0.47-0.88)	0.006	0.04	0
cg15696627	MSX1	4487	TRUE	0.59(0.41-0.86)	0.006	0.04	0
cg27377213	PPP1R16B	26051	TRUE	1.49(1.12-2)	0.006	0.04	0
cg26293512	TEPP	374739	FALSE	1.57(1.13-2.17)	0.006	0.04	0
cg01109219	RASGRP3	25780	TRUE	1.62(1.16-2.26)	0.006	0.04	0
cg19286986	GPR162	27239	TRUE	1.62(1.15-2.27)	0.006	0.04	0
cg02425732	RRBP1	6238	TRUE	0.61(0.43-0.87)	0.006	0.04	0
cg20122476	PITPNA	5306	TRUE	1.56(1.15-2.11)	0.006	0.04	0
cg25141995	VDAC1	7416	TRUE	0.66(0.49-0.89)	0.006	0.04	0

cg04268405	CHST3	9469	TRUE	0.67(0.5-0.9)	0.006	0.04	0
cg08817120	DKFZP686A01247	22998	TRUE	0.65(0.47-0.88)	0.006	0.04	0
cg26847866	SCARA3	51435	FALSE	1.54(1.14-2.07)	0.006	0.04	0
cg06667406	AASS	10157	FALSE	0.64(0.47-0.88)	0.006	0.04	0
cg16019273	EIF2C4	192670	TRUE	0.64(0.46-0.88)	0.006	0.04	0
cg07753644	P2RY11	5032	FALSE	0.65(0.47-0.89)	0.006	0.04	0
cg10198932	C21orf129	150135	FALSE	1.53(1.13-2.06)	0.006	0.04	0
cg05764376	THAP10	56906	TRUE	0.53(0.34-0.83)	0.006	0.04	0
cg04640886	C19orf30	284424	TRUE	0.63(0.45-0.87)	0.006	0.04	0
cg20537629	MAGI2	9863	TRUE	0.63(0.45-0.88)	0.006	0.04	0
cg19688503	CAPN6	827	FALSE	1.56(1.14-2.14)	0.006	0.04	0
cg04999691	C7orf29	113763	FALSE	0.65(0.48-0.88)	0.006	0.04	0
cg14701962	C1orf111	284680	FALSE	1.52(1.12-2.06)	0.006	0.04	0
cg05341878	RIMS2	9699	FALSE	1.67(1.16-2.42)	0.006	0.04	0
cg22892904	CBX2	84733	TRUE	0.64(0.46-0.89)	0.006	0.04	−1
cg12526025	LARGE	9215	TRUE	0.66(0.49-0.89)	0.006	0.04	0
cg16521836	KIAA1407	57577	TRUE	1.49(1.12-1.98)	0.006	0.04	0
cg25827666	NTRK1	4914	FALSE	1.56(1.13-2.16)	0.006	0.04	0
cg25372195	DCD	117159	FALSE	1.54(1.13-2.1)	0.006	0.04	0
cg09354331	RAI17	57178	FALSE	1.58(1.14-2.19)	0.006	0.04	0
cg25126052	INT4	92105	TRUE	1.51(1.12-2.05)	0.006	0.04	0
cg00657095	PPARD	5467	TRUE	1.5(1.12-2)	0.006	0.04	0
cg07846167	FBLIM1	54751	TRUE	0.64(0.47-0.88)	0.006	0.04	0
cg02699167	FBXL2	25827	TRUE	0.61(0.43-0.86)	0.006	0.04	0
cg14046365	NMI	9111	TRUE	1.81(1.19-2.75)	0.006	0.04	0
cg08820801	FBXO17	115290	TRUE	0.66(0.49-0.89)	0.006	0.04	0
cg17563769	GTF2A2	2958	TRUE	1.7(1.16-2.5)	0.006	0.04	0

cg22908581	CD4	920	FALSE	1.53(1.13-2.07)	0.006	0.04	0
cg02175308	SORT1	6272	TRUE	0.64(0.46-0.88)	0.006	0.04	0
cg23843812	FLJ43582	389649	FALSE	0.63(0.45-0.88)	0.006	0.04	0
cg25076881	OR10J1	26476	FALSE	1.53(1.12-2.08)	0.006	0.04	0
cg17003970	CHFR	55743	TRUE	0.68(0.51-0.89)	0.006	0.04	0
cg18742893	BOC	91653	TRUE	0.65(0.47-0.88)	0.006	0.04	0
cg19949550	ASB2	51676	FALSE	1.55(1.12-2.13)	0.006	0.04	0
cg16293656	FGF17	8822	FALSE	0.63(0.45-0.88)	0.006	0.04	0
cg27592112	GABRG3	2567	FALSE	0.67(0.5-0.9)	0.006	0.04	0
cg06874144	CAB39	51719	TRUE	0.66(0.48-0.89)	0.006	0.04	0
cg03399971	INHBC	3626	FALSE	0.67(0.5-0.9)	0.006	0.04	0
cg01361446	IL2RG	3561	FALSE	1.49(1.11-2)	0.006	0.04	0
cg05556210	ATBF1	463	TRUE	0.66(0.48-0.89)	0.006	0.04	0
cg18470891	FOXM1	2305	TRUE	0.66(0.48-0.89)	0.006	0.04	−1
cg26132665	C6orf165	154313	TRUE	1.56(1.12-2.16)	0.006	0.04	0
cg23213217	DEGS1	8560	TRUE	1.54(1.12-2.11)	0.006	0.04	0
cg17687282	A4GNT	51146	FALSE	1.67(1.17-2.38)	0.007	0.04	0
cg21964481	SLC34A3	142680	FALSE	1.54(1.13-2.1)	0.007	0.04	0
cg04544498	MAGEE1	57692	FALSE	1.52(1.13-2.04)	0.007	0.04	0
cg06905862	TPP1	1200	FALSE	1.52(1.12-2.06)	0.007	0.04	0
cg01227519	SPANXD	64648	FALSE	0.66(0.49-0.88)	0.007	0.04	0
cg24428913	WAS	7454	FALSE	0.66(0.49-0.9)	0.007	0.04	0
cg20843052	KIAA1841	84542	TRUE	0.66(0.48-0.89)	0.007	0.04	0
cg18292394	MAT1A	4143	FALSE	1.5(1.12-2)	0.007	0.04	0
cg08812504	QSCN6	5768	TRUE	1.58(1.14-2.2)	0.007	0.04	0
cg21902544	CBLN2	147381	TRUE	0.66(0.48-0.89)	0.007	0.04	0
cg19776453	CEACAM1	634	FALSE	0.66(0.49-0.89)	0.007	0.04	0

cg20870362	CCIN	881	FALSE	0.65(0.47-0.89)	0.007	0.04	0
cg17558126	RASSF5	83593	TRUE	0.63(0.45-0.88)	0.007	0.04	0
cg25423111	TSSK4	283629	FALSE	0.65(0.47-0.89)	0.007	0.04	0
cg04886198	ALG3	10195	TRUE	1.54(1.12-2.12)	0.007	0.04	0
cg03842617	NOS3	4846	FALSE	1.66(1.15-2.4)	0.007	0.04	0
cg04749372	INMT	11185	FALSE	1.59(1.14-2.23)	0.007	0.04	0
cg10984852	ECHS1	1892	TRUE	1.53(1.12-2.1)	0.007	0.04	0
cg24428042	ECHDC3	79746	TRUE	0.63(0.45-0.88)	0.007	0.04	0
cg21200380	C1QBP	708	TRUE	1.54(1.13-2.11)	0.007	0.04	0
cg09752703	BCL2	596	TRUE	1.66(1.15-2.38)	0.007	0.04	0
cg09819033	CREBL2	1389	TRUE	1.49(1.12-2)	0.007	0.04	0
cg15916061	SLC17A4	10050	FALSE	1.54(1.12-2.12)	0.007	0.04	0
cg26160573	PTPN18	26469	TRUE	0.67(0.51-0.9)	0.007	0.04	0
cg04510262	PTPN2	5771	TRUE	1.57(1.13-2.18)	0.007	0.04	0
cg02868338	EXOSC4	54512	TRUE	0.65(0.47-0.89)	0.007	0.04	0
cg03498559	UGT2B4	7363	FALSE	0.63(0.45-0.89)	0.007	0.04	0
cg19703826	ZNF213	7760	TRUE	1.54(1.13-2.11)	0.007	0.04	0
cg13372488	KCNT2	343450	FALSE	1.62(1.14-2.3)	0.007	0.04	0
cg23022999	FLJ45909	126432	FALSE	1.61(1.14-2.29)	0.007	0.04	0
cg10909324	EHMT2	10919	TRUE	1.55(1.13-2.13)	0.007	0.04	0
cg21302727	TACC2	10579	FALSE	0.61(0.43-0.87)	0.007	0.04	0
cg20425293	MRPL42	28977	TRUE	1.54(1.13-2.11)	0.007	0.04	0
cg21053323	SUMO3	6612	TRUE	0.66(0.49-0.88)	0.007	0.04	0
cg20950277	TNIP3	79931	FALSE	0.63(0.44-0.89)	0.007	0.04	0
cg04544154	ELF4	2000	TRUE	0.64(0.47-0.89)	0.007	0.04	0
cg25483839	RAD23B	5887	TRUE	1.56(1.14-2.13)	0.007	0.04	0
cg21553596	FAM3B	54097	TRUE	0.64(0.47-0.87)	0.007	0.04	0

cg02398725	ZF	58487	TRUE	1.57(1.13-2.17)	0.007	0.04	0
cg22658979	MMP13	4322	FALSE	0.65(0.47-0.88)	0.007	0.04	0
cg02719634	SLC22A18AS	5003	FALSE	0.64(0.47-0.87)	0.007	0.04	0
cg03076871	STX16	8675	TRUE	1.52(1.12-2.06)	0.007	0.04	0
cg22464423	IGSF4C	199731	TRUE	1.51(1.11-2.03)	0.007	0.04	0
cg25608041	TBC1D1	23216	TRUE	0.65(0.47-0.89)	0.007	0.04	0
cg20787196	ITPR2	3709	TRUE	0.61(0.43-0.86)	0.007	0.04	0
cg23517677	POLR2G	5436	FALSE	0.59(0.4-0.86)	0.007	0.04	0
cg06043190	EML4	27436	TRUE	1.62(1.13-2.3)	0.007	0.04	0
cg25926679	ZNF512	84450	TRUE	1.52(1.12-2.07)	0.007	0.04	0
cg18589858	SLCO2B1	11309	FALSE	0.63(0.44-0.88)	0.007	0.04	0
cg00893987	RPL7L1	285855	TRUE	1.58(1.13-2.21)	0.007	0.04	0
cg15645309	BATF	10538	FALSE	1.54(1.12-2.13)	0.007	0.04	0
cg19393006	TSC1	7248	FALSE	1.5(1.11-2.03)	0.007	0.04	0
cg04514981	C18orf37	125476	TRUE	1.53(1.12-2.1)	0.007	0.04	0
cg23509869	LST1	7940	FALSE	1.54(1.12-2.13)	0.007	0.04	0
cg20769102	POLR2K	5440	TRUE	1.55(1.12-2.15)	0.007	0.04	0
cg17179881	BIK	638	TRUE	1.55(1.12-2.15)	0.007	0.04	0
cg16606638	GALNT4	8693	TRUE	0.66(0.49-0.9)	0.007	0.04	0
cg22340747	GATM	2628	FALSE	0.66(0.49-0.89)	0.007	0.04	0
cg25165199	RCBTB2	1102	FALSE	0.68(0.51-0.91)	0.007	0.04	−1
cg06456031	FLJ11000	55281	FALSE	1.62(1.13-2.31)	0.007	0.04	0
cg13149307	PLXDC2	84898	TRUE	0.64(0.46-0.89)	0.007	0.04	0
cg08693206	AP1B1	162	TRUE	1.5(1.12-2.03)	0.007	0.04	0
cg20985014	WISP3	8838	FALSE	1.56(1.13-2.17)	0.007	0.04	0
cg22730042	U2AF1	7307	TRUE	0.62(0.44-0.87)	0.007	0.04	0
cg21561142	DEK	7913	TRUE	1.52(1.12-2.06)	0.007	0.04	0

cg20950011	CIDEA	1149	TRUE	0.66(0.49-0.89)	0.007	0.04	0
cg07799947	NPAS2	4862	TRUE	1.56(1.13-2.16)	0.007	0.04	0
cg17001430	KIF25	3834	FALSE	1.66(1.14-2.41)	0.007	0.04	0
cg23877831	MRPL51	51258	TRUE	1.55(1.13-2.12)	0.007	0.04	0
cg07551659	CGNL1	84952	TRUE	0.65(0.48-0.89)	0.007	0.04	0
cg15305343	NSUN4	387338	TRUE	1.48(1.12-1.97)	0.007	0.04	0
cg07034561	TSGA2	89765	TRUE	1.53(1.13-2.06)	0.007	0.04	0
cg07595113	MYBL1	4603	TRUE	1.57(1.13-2.17)	0.007	0.04	0
cg13993218	INS	3630	FALSE	1.59(1.15-2.2)	0.007	0.04	0
cg11846956	KLK10	5655	TRUE	0.61(0.43-0.87)	0.007	0.04	0
cg03135127	TRMT1	55621	TRUE	1.52(1.11-2.07)	0.007	0.04	0
cg02804166	PHF6	84295	TRUE	1.51(1.12-2.05)	0.007	0.04	0
cg11835197	TFAP2E	339488	TRUE	0.64(0.46-0.89)	0.007	0.04	0
cg05418129	C9orf26	90865	FALSE	0.63(0.45-0.89)	0.007	0.04	0
cg14520511	KRIT1	889	TRUE	1.59(1.13-2.23)	0.007	0.04	0
cg02204046	MYCN	4613	TRUE	0.66(0.49-0.89)	0.007	0.04	0
cg21233722	DOCK2	1794	TRUE	1.66(1.15-2.39)	0.007	0.04	0
cg02962602	CCDC57	284001	TRUE	1.49(1.11-2)	0.007	0.05	0
cg17571291	BLVRA	644	TRUE	0.67(0.51-0.9)	0.007	0.05	0
cg01871963	LCN2	3934	FALSE	1.56(1.13-2.15)	0.007	0.05	0
cg18239753	KHDRBS2	202559	TRUE	0.66(0.49-0.89)	0.007	0.05	0
cg01683883	CMTM2	146225	TRUE	0.59(0.41-0.87)	0.007	0.05	0
cg07778029	HOXA9	3205	TRUE	0.67(0.5-0.9)	0.007	0.05	0
cg05215575	FLJ25410	124404	FALSE	1.49(1.11-1.99)	0.007	0.05	0
cg15600835	raptor	57521	TRUE	1.52(1.11-2.08)	0.007	0.05	0
cg24889744	C20orf28	25876	TRUE	0.63(0.45-0.89)	0.007	0.05	0
cg02564061	C12orf25	84070	FALSE	0.66(0.49-0.9)	0.007	0.05	0

cg03562120	WISP2	8839	TRUE	0.63(0.45-0.88)	0.007	0.05	0
cg17022479	FAF1	11124	TRUE	1.58(1.13-2.22)	0.007	0.05	0
cg19279346	LILRB2	10288	FALSE	0.67(0.5-0.9)	0.007	0.05	0
cg15051063		5088	FALSE	1.5(1.11-2.02)	0.007	0.05	0
cg22621695	DYRK2	8445	TRUE	1.69(1.15-2.48)	0.007	0.05	0
cg18047970	GADL1	339896	FALSE	0.66(0.48-0.9)	0.007	0.05	0
cg21256649	PRKAR1A	5573	FALSE	0.66(0.49-0.9)	0.007	0.05	−1
cg11094938	ATP2A1	487	FALSE	1.54(1.12-2.12)	0.007	0.05	0
cg22122449	ACR	49	FALSE	1.65(1.16-2.35)	0.007	0.05	0
cg08983259	INSL5	10022	TRUE	0.62(0.44-0.89)	0.007	0.05	0
cg08737421	MAX	4149	TRUE	1.5(1.11-2.02)	0.007	0.05	0
cg12497564	RBP1	5947	TRUE	0.62(0.44-0.87)	0.007	0.05	0
cg08965235	LTBP3	4054	TRUE	0.67(0.5-0.89)	0.007	0.05	0
cg14568338	IGF1R	3480	TRUE	1.57(1.12-2.2)	0.007	0.05	0
cg26521448	ZC3H7A	29066	TRUE	1.61(1.13-2.3)	0.007	0.05	0
cg03117949	USP16	10600	TRUE	1.54(1.12-2.12)	0.007	0.05	0
cg11308840	LOC401498	401498	TRUE	0.63(0.46-0.88)	0.007	0.05	0
cg03620376	CDC42EP5	148170	FALSE	0.64(0.46-0.89)	0.007	0.05	0
cg14587868	TGM1	7051	FALSE	1.54(1.12-2.11)	0.007	0.05	0
cg14727512	TSSK2	23617	TRUE	0.64(0.46-0.89)	0.007	0.05	0
cg10417559	LMO6	4007	FALSE	0.62(0.45-0.87)	0.007	0.05	0
cg16812893	KRTAP15-1	254950	FALSE	1.55(1.12-2.16)	0.007	0.05	0
cg24724583	PLXDC1	57125	FALSE	0.68(0.51-0.9)	0.007	0.05	0
cg27037648	CASKIN2	57513	TRUE	0.65(0.47-0.89)	0.007	0.05	0
cg00729875	PALMD	54873	FALSE	1.5(1.11-2.02)	0.007	0.05	0
cg23867494	TNFRSF4	7293	FALSE	1.49(1.11-2)	0.007	0.05	0
cg26608032	MKL1	57591	TRUE	1.52(1.14-2.05)	0.007	0.05	0

cg03473518	GJB6	10804	TRUE	0.63(0.45-0.88)	0.007	0.05	0
cg07341907	UNC13B	10497	TRUE	1.61(1.13-2.28)	0.007	0.05	0
cg13784792	JUP	3728	TRUE	0.66(0.48-0.9)	0.007	0.05	0
cg03148461	BRAF	673	TRUE	1.52(1.11-2.07)	0.008	0.05	0
cg13599007	FABP6	2172	FALSE	0.59(0.4-0.86)	0.008	0.05	0
cg26711820	MYF6	4618	TRUE	1.53(1.11-2.11)	0.008	0.05	0
cg02990612	GATA4	2626	TRUE	1.51(1.11-2.06)	0.008	0.05	0
cg04432009	LYL1	4066	TRUE	1.52(1.12-2.06)	0.008	0.05	0
cg07246225	ZFHX1B	9839	TRUE	1.61(1.14-2.29)	0.008	0.05	0
cg23540745	HIST1H4G	8369	TRUE	0.58(0.39-0.87)	0.008	0.05	0
cg22752533	SLC12A5	57468	TRUE	0.66(0.49-0.89)	0.008	0.05	0
cg10065130	COG5	10466	TRUE	1.47(1.11-1.96)	0.008	0.05	0
cg25374813	SLC23A1	9963	FALSE	0.67(0.5-0.9)	0.008	0.05	0
cg02246665	HCFC2	29915	TRUE	1.54(1.12-2.11)	0.008	0.05	0
cg24987706	GPA33	10223	FALSE	0.63(0.44-0.89)	0.008	0.05	0
cg04675937	CDKN2B	1030	TRUE	0.64(0.46-0.88)	0.008	0.05	0
cg20376899	EDN1	1906	TRUE	1.52(1.12-2.08)	0.008	0.05	0
cg03388193	HPSE2	60495	TRUE	1.55(1.12-2.14)	0.008	0.05	0
cg10124491	MGC29891	126626	TRUE	1.91(1.19-3.08)	0.008	0.05	0
cg24642820	NUP210	23225	TRUE	1.47(1.1-1.97)	0.008	0.05	0
cg16666160	NR5A1	2516	TRUE	1.55(1.13-2.13)	0.008	0.05	0
cg00060882	EIF2S3	1968	TRUE	1.51(1.11-2.06)	0.008	0.05	0
cg20663831	GIMAP2	26157	FALSE	1.68(1.13-2.48)	0.008	0.05	0
cg06493386	TRPA1	8989	TRUE	0.62(0.44-0.88)	0.008	0.05	0
cg17524624	GSTK1	373156	TRUE	1.51(1.11-2.05)	0.008	0.05	0
cg22717127	CBWD2	150472	TRUE	0.62(0.44-0.87)	0.008	0.05	0
cg25194415	KIAA0232	9778	TRUE	1.62(1.13-2.33)	0.008	0.05	0

cg21407055	ART1	417	FALSE	1.58(1.13-2.22)	0.008	0.05	0
cg22792432	ROCK1	6093	TRUE	1.52(1.12-2.06)	0.008	0.05	0
cg26145103	MGC17839	219902	TRUE	0.63(0.44-0.89)	0.008	0.05	0
cg09352789	XPNPEP1	7511	TRUE	1.53(1.13-2.07)	0.008	0.05	0
cg15272684	KIAA0319L	79932	FALSE	0.63(0.44-0.88)	0.008	0.05	0
cg06214007	GBP6	163351	FALSE	1.51(1.11-2.05)	0.008	0.05	0
cg24895052	MPP3	4356	FALSE	0.63(0.45-0.88)	0.008	0.05	0
cg16872560	PPP1R9A	55607	TRUE	0.65(0.47-0.89)	0.008	0.05	0
cg16842214	KBTBD5	131377	TRUE	1.48(1.1-1.98)	0.008	0.05	0
cg13160888	NIPSNAP3B	55335	TRUE	1.49(1.1-2.01)	0.008	0.05	0
cg19734228	DAPK1	1612	TRUE	0.62(0.45-0.88)	0.008	0.05	0
cg21665774	KIAA0355	9710	FALSE	0.66(0.48-0.9)	0.008	0.05	0
cg26845278	SPATA21	374955	FALSE	1.52(1.11-2.08)	0.008	0.05	0
cg25475443	GALNAC4S-6ST	51363	FALSE	1.55(1.12-2.15)	0.008	0.05	0
cg11492403	AARSD1	80755	TRUE	0.64(0.46-0.89)	0.008	0.05	0
cg03417317	C10orf77	79847	TRUE	1.64(1.13-2.38)	0.008	0.05	0
cg02367951	HIST1H2AK	8330	TRUE	1.53(1.12-2.09)	0.008	0.05	0
cg23978557	SMPD2	6610	TRUE	0.52(0.33-0.83)	0.008	0.05	0
cg02309273	INPP5B	3633	FALSE	0.65(0.47-0.9)	0.008	0.05	0
cg20668607	DNAJC11	55735	TRUE	0.67(0.5-0.9)	0.008	0.05	0
cg01255349	PPFIBP1	8496	TRUE	1.73(1.16-2.58)	0.008	0.05	0
cg00030047	C1orf188	148646	TRUE	0.66(0.49-0.9)	0.008	0.05	0
cg19481686	CDKN2B	1030	TRUE	0.67(0.49-0.9)	0.008	0.05	0
cg01566170	CAPN2	824	TRUE	1.49(1.11-2.01)	0.008	0.05	0
cg04968473	CYP1A2	1544	FALSE	1.56(1.12-2.15)	0.008	0.05	0
cg18440048	ZNF70	7621	TRUE	0.66(0.48-0.9)	0.008	0.05	0
cg23182299	LHX5	64211	TRUE	0.64(0.46-0.89)	0.008	0.05	0

cg17644208	ADCY3	109	TRUE	1.52(1.12-2.08)	0.008	0.05	0
cg15712267	DNAJB1	3337	TRUE	0.67(0.5-0.9)	0.008	0.05	0
cg05475904	BCL10	8915	FALSE	0.67(0.5-0.9)	0.008	0.05	0
cg26709950	RRAD	6236	TRUE	1.59(1.12-2.25)	0.008	0.05	0
cg13017345	MXI1	4601	TRUE	0.68(0.5-0.91)	0.008	0.05	0
cg01939428	UBE3C	9690	TRUE	1.48(1.11-1.98)	0.008	0.05	0
cg10773309	BAI2	576	TRUE	1.56(1.12-2.16)	0.008	0.05	0
cg18670235	PLOD3	8985	TRUE	0.68(0.51-0.91)	0.008	0.05	0
cg13199589	FAM80A	284716	TRUE	0.66(0.48-0.9)	0.008	0.05	0
cg25565479	PANX1	24145	TRUE	1.54(1.12-2.11)	0.008	0.05	0
cg16620731	ZNF583	147949	TRUE	2.24(1.24-4.05)	0.008	0.05	0
cg03754063	KEAP1	9817	TRUE	0.64(0.46-0.89)	0.008	0.05	0
cg05871136	UNC5B	219699	TRUE	0.64(0.46-0.9)	0.008	0.05	0
cg11935147	PDE4DIP	9659	TRUE	0.65(0.48-0.88)	0.008	0.05	0
cg19949137	HTATSF1	27336	TRUE	1.65(1.14-2.38)	0.008	0.05	0
cg09411366	PEPD	5184	FALSE	1.48(1.11-1.98)	0.008	0.05	0
cg02774160	GGT1	2678	FALSE	0.63(0.45-0.89)	0.008	0.05	0
cg21246431	MGC40579	256356	TRUE	1.47(1.1-1.95)	0.008	0.05	0
cg17280624	PGRMC2	10424	TRUE	1.53(1.11-2.11)	0.008	0.05	0
cg27440834	SNX4	8723	FALSE	0.68(0.51-0.91)	0.008	0.05	−1
cg20017147	TEX101	83639	FALSE	0.67(0.49-0.9)	0.008	0.05	0
cg23037403	ZNF454	285676	TRUE	0.64(0.47-0.89)	0.008	0.05	0
cg06226384	CACNG5	27091	FALSE	1.52(1.12-2.06)	0.008	0.05	0
cg07549194	TIP39	113091	FALSE	1.49(1.1-2)	0.008	0.05	0
cg03837750	LRRC44	127255	TRUE	1.58(1.12-2.23)	0.008	0.05	0
cg04725234	TMEM38B	55151	TRUE	0.65(0.47-0.9)	0.008	0.05	0
cg05181279	RIG	10530	FALSE	1.51(1.1-2.07)	0.008	0.05	0

cg07423906	C17orf56	146705	TRUE	0.65(0.47-0.9)	0.008	0.05	0
cg09805010	THRB	7068	TRUE	0.65(0.48-0.89)	0.008	0.05	0
cg09450020	STEAP2	261729	TRUE	0.66(0.49-0.9)	0.008	0.05	0
cg12506971	AADAT	51166	TRUE	0.61(0.42-0.87)	0.008	0.05	0
cg01427567	TMC4	147798	FALSE	1.49(1.12-1.98)	0.008	0.05	0
cg26059153	C20orf133	140733	TRUE	0.61(0.42-0.87)	0.008	0.05	0
cg19982860	IFNA21	3452	FALSE	1.54(1.12-2.11)	0.008	0.05	0
cg18027946	C21orf2	755	TRUE	1.56(1.12-2.16)	0.008	0.05	0
cg10904672	FCGR1A	2209	FALSE	1.49(1.11-2.02)	0.008	0.05	0
cg01169778	GBGT1	26301	TRUE	0.67(0.49-0.9)	0.008	0.05	0
cg15780361	ALS2CR11	151254	TRUE	0.69(0.52-0.91)	0.008	0.05	0
cg19988449	BNC1	646	TRUE	0.61(0.42-0.87)	0.008	0.05	0
cg22335340	PTPN6	5777	FALSE	1.52(1.11-2.08)	0.008	0.05	0
cg14602164	FBXO8	26269	TRUE	1.51(1.11-2.06)	0.008	0.05	0
cg02523617	HIP2	3093	TRUE	1.6(1.12-2.28)	0.008	0.05	0
