# Supplementary material for: An Epigenetic Signature in Peripheral Blood Predicts Active Ovarian Cancer
Source: PLoS One. 2009 Dec 18;4(12):e8274. doi: 10.1371/journal.pone.0008274 (PMC2793425; doi:10.1371/journal.pone.0008274)
Supplement: Table S5 — Discriminatory CpGs between postreatment samples without active disease and healthy controls. (0.04 MB PDF) [file pone.0008274.s011.pdf]

IlmnID	Symbol	Gene_ID	CPG_ISLAND	OR (95% CI)	P-val	Q-val	CA-diagnostic CpG?
cg10986043	TCAP	8557	FALSE	4.36(2.55-7.43)	7E−08	0.002	NO
cg06653796	LIME1	54923	TRUE	3.39(2.06-5.58)	2E−06	0.019	YES
cg19287277	RKHD3	84206	TRUE	0.09(0.03-0.28)	4E−05	0.314	NO
cg15096140	MYO1B	4430	TRUE	0.41(0.26-0.63)	8E−05	0.361	NO
cg11481351	FNDC7	163479	FALSE	0.43(0.29-0.66)	0.0001	0.361	NO
cg02679745	FUT7	2529	FALSE	0.24(0.12-0.5)	0.0001	0.361	YES
cg01254505	BST2	684	FALSE	2.2(1.47-3.31)	0.0001	0.361	NO
cg18105315	MANEAL	149175	TRUE	0.35(0.2-0.6)	0.0001	0.361	NO
cg11471401	KRT6A	3853	FALSE	2.4(1.53-3.76)	0.0001	0.361	NO
cg00973286	TNFRSF1A	7132	FALSE	1.99(1.39-2.84)	0.0002	0.361	NO
cg05472874	SULT4A1	25830	TRUE	3.85(1.9-7.81)	0.0002	0.361	NO
cg18676803	MYNN	55892	TRUE	0.45(0.29-0.68)	0.0002	0.361	NO
cg13379236	EGF	1950	FALSE	2.06(1.4-3.03)	0.0002	0.361	NO
cg05135288	RHOT2	89941	FALSE	2.29(1.47-3.56)	0.0002	0.361	NO
cg07732037	MPHOSPH9	10198	FALSE	2.19(1.44-3.33)	0.0002	0.361	YES
cg14520448	PQBP1	10084	TRUE	2.54(1.54-4.18)	0.0002	0.361	NO
cg04032871	QPCTL	54814	TRUE	1.99(1.37-2.9)	0.0003	0.37	NO
cg10313633	TP53I11	9537	TRUE	0.48(0.33-0.72)	0.0003	0.37	NO
cg03547797	GAS2	2620	FALSE	2.05(1.39-3.04)	0.0003	0.37	NO
cg22189286	HSPB8	26353	FALSE	2.08(1.39-3.11)	0.0004	0.37	NO
cg21787291	TCBA1	154215	TRUE	0.15(0.05-0.43)	0.0004	0.37	NO
cg16046376	PC	5091	FALSE	3.09(1.66-5.76)	0.0004	0.37	YES
cg12937807	LPHN1	22859	TRUE	2.39(1.47-3.87)	0.0004	0.37	NO
cg02245418	ZNF364	27246	FALSE	0.41(0.25-0.67)	0.0004	0.37	YES
cg26431343	HSPC117	51493	FALSE	0.51(0.35-0.74)	0.0004	0.37	NO
cg05600717	FLJ13639	79758	TRUE	2.7(1.55-4.7)	0.0005	0.37	NO

cg19965810	KCNH7	90134	TRUE	3.04(1.63-5.66)	0.0005	0.37	NO
cg10929387	ITIH4	3700	FALSE	2.11(1.39-3.22)	0.0005	0.37	NO
cg23995778	CGB5	93659	TRUE	0.49(0.32-0.73)	0.0005	0.37	NO
cg10694152	SLC15A1	6564	TRUE	2.3(1.44-3.69)	0.0005	0.37	NO
cg11529128	LHFPL5	222662	TRUE	0.41(0.25-0.68)	0.0005	0.37	NO
cg15279364	TMEM130	222865	TRUE	0.43(0.27-0.69)	0.0005	0.37	NO
cg10467098	Bles03	83638	FALSE	2.06(1.37-3.11)	0.0005	0.37	NO
cg23303408	POU4F3	5459	TRUE	2.82(1.57-5.1)	0.0006	0.377	NO
cg16142218	CHMP7	91782	FALSE	0.37(0.21-0.65)	0.0006	0.377	YES
cg19202384	PYCR1	5831	TRUE	0.46(0.3-0.72)	0.0006	0.377	NO
cg16890093	PSMB8	5696	FALSE	0.42(0.25-0.69)	0.0007	0.377	NO
cg07211259	PDCD1LG2	80380	FALSE	1.89(1.31-2.73)	0.0007	0.377	NO
cg18780401	OTUD5	55593	TRUE	2.66(1.51-4.69)	0.0007	0.377	NO
cg05004940	C20orf195	79025	TRUE	1.86(1.3-2.68)	0.0007	0.377	NO
cg26718122	SFRS6	6431	TRUE	3.15(1.62-6.14)	0.0007	0.377	YES
cg18788940	HTATIP2	10553	TRUE	0.52(0.35-0.76)	0.0008	0.377	NO
cg11799561	SMR3A	26952	FALSE	0.43(0.26-0.7)	0.0008	0.377	NO
cg15690721	ZNF85	7639	FALSE	0.46(0.29-0.73)	0.0008	0.377	NO
cg18113270	MMP3	4314	FALSE	0.54(0.38-0.77)	0.0008	0.377	NO
cg26777475	PCOLCE	5118	FALSE	2.27(1.41-3.66)	0.0008	0.377	NO
cg25288155	PLEKHB1	58473	FALSE	0.51(0.34-0.75)	0.0008	0.377	NO
cg12243271	CFI	3426	FALSE	0.41(0.25-0.69)	0.0008	0.377	YES
cg18056600	ZMYND15	84225	TRUE	0.46(0.29-0.72)	0.0008	0.377	NO
cg26746469	KIAA0406	9675	FALSE	0.3(0.15-0.61)	0.0008	0.377	NO
cg19714296	CYP4A11	1579	FALSE	0.56(0.4-0.79)	0.0008	0.377	NO
cg18638581	HK2	3099	FALSE	0.19(0.07-0.5)	0.0009	0.377	YES
cg00406844	RFPL3	10738	FALSE	2.42(1.43-4.07)	0.0009	0.377	YES

cg24082826	ITPR2	3709	TRUE	0.52(0.36-0.77)	0.0009	0.377	NO
cg12400041	DST	667	TRUE	0.49(0.32-0.75)	0.0009	0.377	YES
cg12619509	DNASE1L2	1775	TRUE	2.33(1.41-3.84)	0.0009	0.377	NO
cg25070480	PPHLN1	51535	TRUE	0.42(0.25-0.7)	0.001	0.377	NO
cg10881306	CRIM1	51232	TRUE	1.85(1.28-2.66)	0.001	0.377	NO
cg19286986	GPR162	27239	TRUE	2.08(1.34-3.2)	0.001	0.377	YES
cg07550362	TAC1	6863	TRUE	0.27(0.12-0.59)	0.001	0.377	NO
cg20979799	RFXDC1	222546	TRUE	2.05(1.33-3.15)	0.001	0.377	NO
cg15674997	GRM6	2916	TRUE	1.9(1.29-2.8)	0.001	0.377	NO
cg05679613	MEPE	56955	FALSE	0.41(0.24-0.7)	0.001	0.377	YES
cg11368509	SUV420H2	84787	TRUE	2.19(1.37-3.51)	0.001	0.377	NO
cg24063382	MAS1L	116511	FALSE	2.13(1.35-3.35)	0.001	0.377	YES
cg22475430	ATN1	1822	FALSE	0.53(0.36-0.78)	0.001	0.377	NO
cg18204685	BTD	686	FALSE	0.44(0.27-0.72)	0.001	0.377	NO
cg05380910	STOML3	161003	FALSE	2.45(1.43-4.2)	0.001	0.377	NO
cg00108454	C1QA	712	FALSE	0.53(0.37-0.78)	0.001	0.377	NO
cg13906416	PVR	5817	TRUE	0.24(0.1-0.57)	0.001	0.377	NO
cg23698956	FAM3A	60343	TRUE	1.94(1.3-2.9)	0.001	0.377	NO
cg01078434	MAS1L	116511	FALSE	2.51(1.44-4.38)	0.001	0.377	YES
cg24731756	ILK	3611	TRUE	1.76(1.25-2.47)	0.001	0.377	NO
cg19573166	SLC22A17	51310	FALSE	1.89(1.29-2.78)	0.001	0.377	NO
cg02053171	WBSCR16	81554	TRUE	1.81(1.26-2.59)	0.001	0.377	YES
cg25410053	ZIC3	7547	TRUE	0.54(0.37-0.79)	0.001	0.38	NO
cg21481775	NKX3-1	4824	TRUE	0.31(0.15-0.63)	0.001	0.38	NO
cg05523047	VLDLR	7436	TRUE	0.22(0.09-0.55)	0.001	0.38	NO
cg01137532	ZNF365	22891	TRUE	0.27(0.12-0.6)	0.001	0.38	NO
cg11911951	C16orf28	65259	TRUE	0.51(0.34-0.77)	0.001	0.39	NO

cg11879514	SLC16A6	9120	TRUE	2.04(1.32-3.17)	0.001	0.39	YES
cg06059810	RUFY3	22902	FALSE	0.46(0.29-0.74)	0.001	0.39	NO
cg13383490	TSG101	7251	TRUE	2.48(1.42-4.33)	0.001	0.39	YES
cg08821669	COX6A1	1337	TRUE	2.36(1.39-4)	0.001	0.39	NO
